# Synthetic approaches for novel 3-heteroaryl-4-hydroxy-1-methylquinoline-2(1*H*)one: spectroscopic characterization, molecular docking and DFT investigations†

**DOI:** 10.1039/d5ra00325c

**Published:** 2025-02-28

**Authors:** Mai A. Mostafa, Magdy A. Ibrahim, S. S. Ibrahim, Nada Mohamed, Al-Shimaa Badran

**Affiliations:** a Department of Chemistry, Faculty of Education, Ain Shams University Roxy Cairo 11711 Egypt badran.shimaa@yahoo.com +2 022581243 +2 01011444940

## Abstract

Ring opening and recyclization reactions with 4-hydroxy-6-methyl-3-nitro-2*H*-pyrano[3,2-*c*]quinoline-2,5(6*H*)-dione (1) was examined towards some carbon nucleophilic reagents. Treatment of key precursor 1 with cyanoacetamide, malononitrile dimer and 1*H*-benzimidazol-2-ylacetonitrile afforded pyridines 2, 3 and pyrido[1,2-*a*]benzimidazole 4, respectively. Reaction of compound 1 with 5-amino-2,4-dihydro-3*H*-pyrazol-3-one and 5-amino-3-methyl-1*H*-pyrazole furnished pyrazolo[3,4-*b*]pyridine derivatives 5 and 6. Further, pyrido[2,3-*d*]pyrimidines 7–9 were synthesized from recyclization of compound 1 with some 6-aminouracil derivatives. Compounds 2–4 demonstrated favorable efficacy in HepG-2 liver cancer cells comparable to reference drug (cisplatin), exhibiting IC_50_ values of 8.79–17.78 μM L^−1^. The optimized geometrical configurations of the synthesized compounds were determined, using DFT calculations conducted at the B3LYP/6-311++G(d,p) level, for computing various parameters including molecular electrostatic potential (MEP), global reactivity indices, frontier molecular orbital (FMO) analysis, and nonlinear optical (NLO) characteristics. The obtained results revealed that, compound 3 exhibits the smallest energy gap (Δ*E* = 2.783 eV), while compound 9 presents the largest energy gap (Δ*E* = 3.995 eV). Also, compound 9 with a hardness value (*η*) of 1.998 eV demonstrated greater hardness and stability compared to other compounds, whereas compound 3 with a softness value (*S*) of 0.719 eV^−1^, is comparatively softer and more reactive. The experimental infrared (IR) and nuclear magnetic resonance (NMR) spectra of the current compounds were compared with the simulated spectra derived from DFT computations, revealing a good agreement. SwissADME analyses suggested that all prepared compounds adhere to the Lipinski, Ghose, and Veber principles fitting to drug likeness. Utilizing the topoisomerase IIβ protein (PDB ID: 4G0U) as a receptor, molecular docking analyses were conducted to investigate the binding interactions of the synthesized compounds and correlated with their anticancer efficacy. Further, MEP surfaces of the studied compounds were analyzed to ascertain the reactive sites appropriated to electrophilic and nucleophilic attacks. The theoretical investigation suggests that the synthesized compounds possess potential applicability for future NLO applications.

## Abstract 2

Ring opening and recyclization reactions with 4-hydroxy-6-methyl-3-nitro-2*H*-pyrano[3,2-*c*]quinoline-2,5(6*H*)-dione (1) was examined towards some carbon nucleophilic reagents.

## Graphical abstract

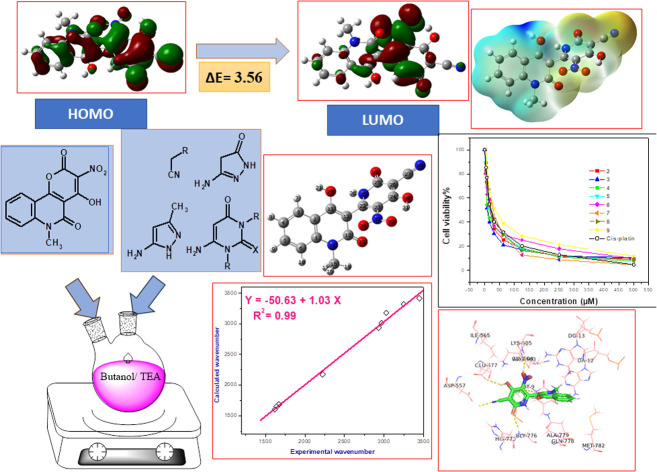

## 1. Introduction

Nitrogen-containing heterocyclic compounds, particularly quinolines, are of significant interest in medicinal and pharmaceutical chemistry due to their diverse biological activities.^1–3^ Basic building component of many natural alkaloids is the quinoline ring structure and widely employed as bioactive scaffolds.^4,5^ Additionally, they are commonly utilized as colorants and dyes in a wide range of commercial industrial field.^6^ The majority of bioactive quinolines, both synthetic and natural, are crucial for the creation of novel pharmacological drugs such as insecticidal,^7^ anthelmintic,^8^ antimicrobial,^9^ anti-inflammatory,^10^ anti-viral,^11^ antioxidant,^3^ antimalarial,^12^ antihypertensive,^13^ antitumor,^14^ antiproliferative,^15^ and antibiotic agents.^16^ Luminescent, fluorescent, thin films, optoelectronic, solvatochromic, optical properties, photodiode photovoltaic fabrication as well as corrosion inhibitors and other applications were recently examined for some quinolines.^17–19^ Pyrano[3,2-*c*]quinoline derivatives are active substrates due to the availability of α-pyrone moiety and were employed as flexible intermediates for building a range of both heteroannulated pyrano[3,2-*c*]quinolines and 3-heteroarylquinolines; through reactions with a variety of carbon and nitrogen nucleophiles.^20–23^ Density Functional Theory (DFT) is one of modeling techniques that is notable for its capacity to precisely compute the physicochemical characteristics of molecules at a cheap computing cost.^24,25^ DFT technique is employed to optimize geometrical configurations of organic compounds and identify nucleophilic and electrophilic sites.^26^ This method makes it easier to fully comprehend how the molecule behaves as an electrophile or nucleophile.^27,28^ DFT is utilized to compute Frontier Molecular Orbitals (FMO), which characterize the electronic characteristic and provide quantum reactivity descriptors. Further, it enables the simulation of infrared red (IR) and NMR spectra.^29^

In this study, various heterocyclic compounds bearing quinoline moiety were synthesized from 4-hydroxy-6-methyl-3-nitro-2*H*-pyrano[3,2-*c*]quinoline-2,5(6*H*)-dione (1) with some carbon nucleophilic reagents. The current compounds were optimized using the DFT/B3LYP/6-311++(d,p) basis set and ascertain their global reactivity descriptors. To investigate the structural properties of these molecules, infrared spectroscopy (IR) and nuclear magnetic resonance (NMR) spectroscopy were computed and compared with experimental results. Bioavailability of synthesized molecules was investigated by ADME analysis and evaluated as potential inhibitors for the topoisomerase IIβ protein (PDB ID: 4G0U). Additionally, MEP maps and the nonlinear optical (NLO) characteristics were calculated for the studied molecules.

## 2. Experimental

Melting points were measured using the digital Stuart SMP3 device. Using KBr discs, infrared spectra were obtained by FTIR Nicolet IS10 spectrophotometer (cm^−1^). Mercury-400BB (400 MHz) was utilized to measure the ^1^H NMR (400 MHz) and ^13^C NMR (100 MHz) spectra. The solvent used was DMSO-d_6_, and the internal standard was TMS (*δ*). The GC-2010 Shimadzu Gas Chromatography Instrument mass spectrometer (70 eV) was used to obtain mass spectra. A PerkinElmer CHN-2400 analyzer was used to conduct elemental microanalyses. 4-Hydroxy-6-methyl-3-nitro-2*H*-pyrano[3,2-*c*]quinoline-2,5(6*H*)-dione (1) was synthesized according to literature.^30^

### 2.1. Synthesis and characterization of compounds

#### 2.1.1. 4-Hydroxy-6-(4-hydroxy-1-methyl-2-oxo-1,2-dihydroquinolin-3-yl)-5-nitro-2-oxo-1,2-dihydropyridine-3-carbonitrile (2)

**Scheme 1:**
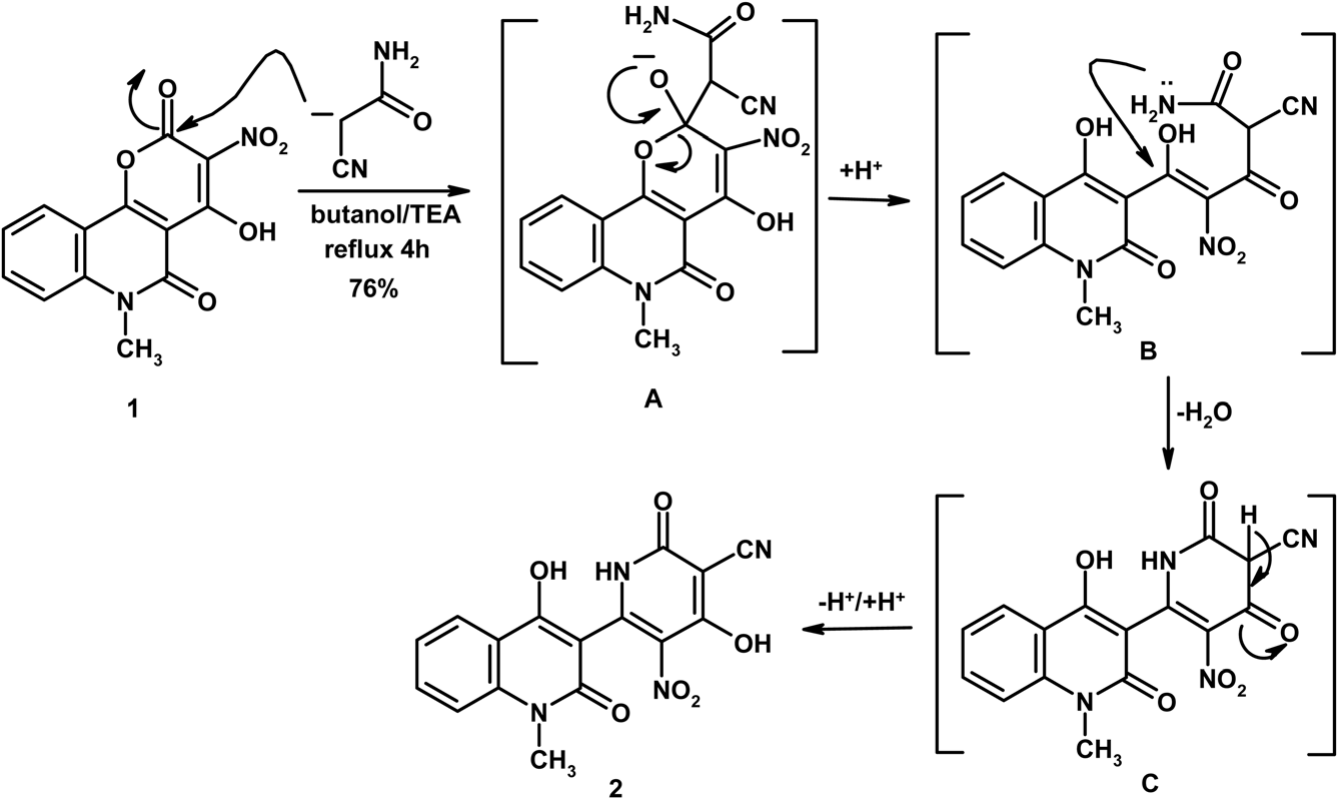
Reaction of precursor 1 with cyanoacetamide.

As shown in Scheme 1, a mixture of 4-hydroxy-6-methyl-3-nitro-2*H*-pyrano[3,2-*c*]quinoline-2,5(6*H*)-dione (1) (0.58 g, 2 mmol) and cyanoacetamide (0.17 g, 2 mmol) in butanol (10 mL) containing TEA (0.1 mL) was heated under reflux for 4 h. The solid obtained after cooling was filtered and crystallized from AcOH to give compound 2 as yellow crystals, yield (0.54 g, 76%), m.p. > 300 °C. IR (KBr, cm^−1^): 3445 (OH), 3248 (NH), 3028 (CH_arom_), 2966, 2931 (CH_aliph_), 2224 (C≡N), 1667 (C=O_pyridone_), 1639 (C=O_quinolone_) and 1617 (C=C). ^1^H NMR (DMSO-d_6_, *δ*, 400 MHz): 3.68 (s, 3H, CH_3_), 7.40 (t, 1H, *J* = 8.0 Hz, H-6), 7.62 (d, 1H, *J* = 8.4 Hz, H-8), 7.81 (t, 1H, *J* = 8.4 Hz, H-7), 8.11 (d, 1H, *J* = 8.0 Hz, H-5), 11.34 (bs, 1H, NH exchangeable with D_2_O), 12.32 (bs, 1H, OH exchangeable with D_2_O), 12.61 (bs, 1H, OH exchangeable with D_2_O). Mass spectrum, *m*/*z* (*I*_r_ %): 354 (M^+^, 100), 326 (42), 310 (29), 280 (49), 259 (55), 237 (21), 213 (40), 175 (53), 159 (26), 133 (12), 105 (18), 93 (40), 77 (32), 64 (11). Anal. calcd. for C_16_H_10_N_4_O_6_ (354.27); C, 54.24; H, 2.85; N, 15.81%. Found: C, 53.96; H, 2.74; N, 15.63%.

#### 2.1.2. 3-Cyano-4-hydroxy-6-(4-hydroxy-1-methyl-2-oxo-1,2-dihydroquinolin-3-yl)-5-nitropyridin-2(1*H*)-ylidene]propanedinitrile (3)

**Scheme 2:**
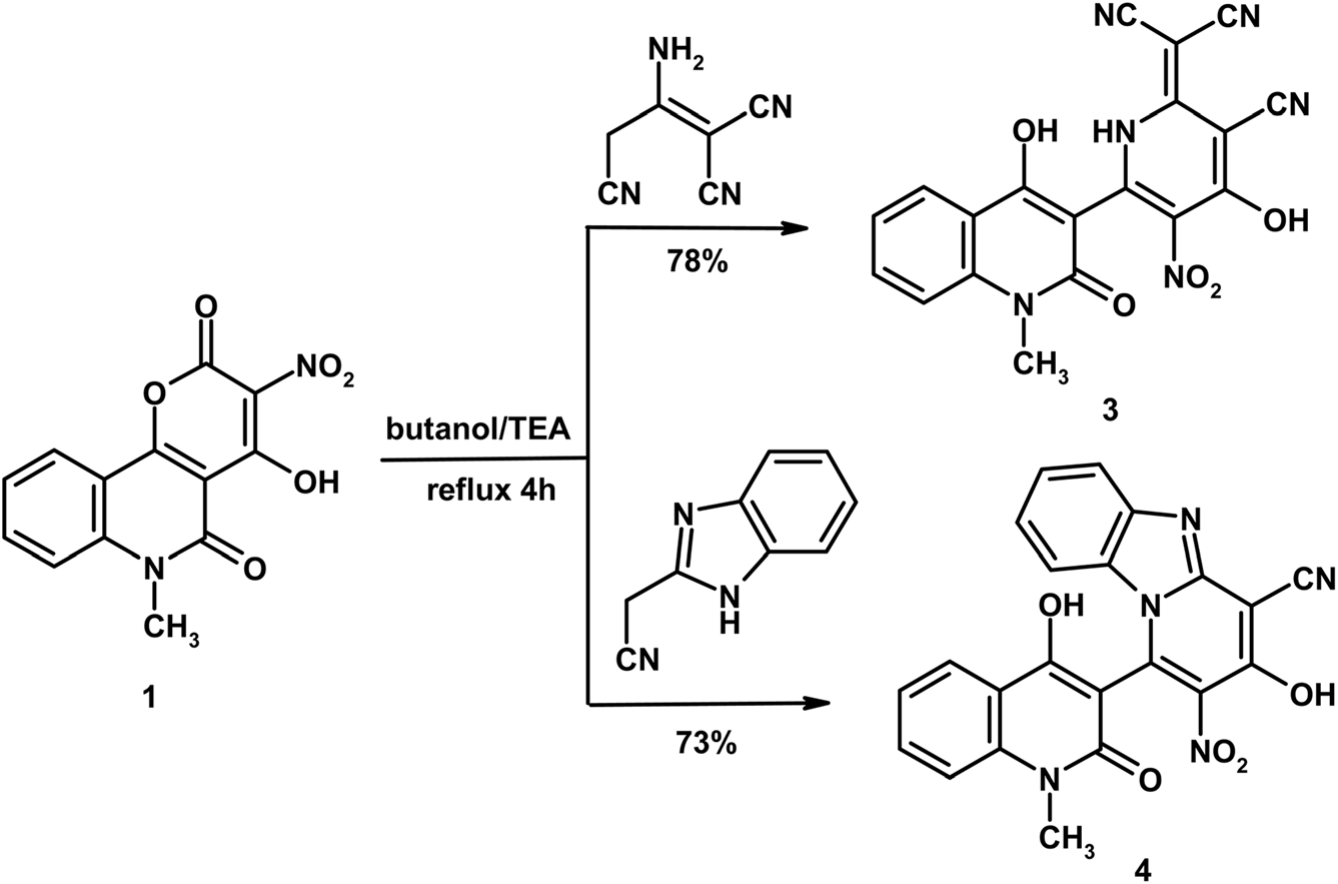
Formation of pyridine 3 and pyrido[1,2-*a*]benzimidazole 4.

As shown in Scheme 2, a mixture of compound 1 (0.58 g, 2 mmol) and 2-aminoprop-1-ene-1,1,3-tricarbonitrile (0.27 g, 2 mmol) in butanol (10 mL) containing TEA (0.1 mL) was heated under reflux for 4 h. The solid obtained after cooling was filtered and crystallized from DMF/H_2_O to give compound 3 as orange-yellow crystals, yield (0.63 g, 78%), m.p. 256–257 °C. IR (KBr, cm^−1^): 3440 (OH), 3281 (NH), 3014 (CH_arom_), 2982, 2936 (CH_aliph_), 2201, 2194, 2174 (3C≡N), 1651 (C=O_quinolone_), 1594 (C=C). ^1^H NMR (DMSO-d_6_, *δ*, 400 MHz): 3.54 (s, 3H, CH_3_), 7.13 (t, 1H, *J* = 7.6 Hz, H-6), 7.33 (d, 1H, *J* = 7.6 Hz, H-8), 7.54 (t, 1H, *J* = 7.6 Hz, H-7), 8.14 (d, 1H, *J* = 7.6 Hz, H-5), 11.46 (bs, 1H, NH exchangeable with D_2_O), 12.39 (bs, 1H, OH exchangeable with D_2_O), 12.79 (bs, 1H, OH exchangeable with D_2_O). Anal. calcd. for C_19_H_10_N_6_O_5_ (402.32); C, 56.72; H, 2.51; N, 20.89%. Found: 56.49; H, 2.32; N, 20.83%.

#### 2.1.3. 3-Hydroxy-1-(4-hydroxy-1-methyl-2-oxo-1,2-dihydroquinolin-3-yl)-2-nitropyrido[1,2-*a*]benzimidazole-4-carbonitrile (4)

As shown in Scheme 2, a mixture of compound 1 (0.58 g, 2 mmol) and 1*H*-benzimidazol-2-ylacetonitrile (0.31 g, 2 mmol) in butanol (10 mL) containing TEA (0.1 mL) was heated under reflux for 4 h. The solid obtained during heating was filtered and crystallized from DMF/EtOH to give compound 4 as yellow crystals, yield (0.62 g, 73%), m.p. > 300 °C. IR (KBr, cm^−1^): 3445 (OH), 3042 (CH_arom_), 2981, 2943 (CH_aliph_), 2211 (C≡N), 1647 (C=O_quinolone_), 1610 (C=N), 1566 (C=C). ^1^H NMR (DMSO-d_6_, *δ*, 400 MHz): 3.62 (s, 3H, CH_3_), 7.23 (t, 1H, *J* = 7.2 Hz, H-6), 7.26–7.34 (m, 2H, 2Ar–H), 7.39 (d, 1H, *J* = 7.2 Hz, H-8), 7.62–7.69 (m, 2H, 2Ar–H), 8.04 (t, 1H, *J* = 8.0 Hz, H-7), 8.21 (d, 1H, *J* = 8.4 Hz, H-5), 12.59 (bs, 1H, OH exchangeable with D_2_O), 13.31 (bs, 1H, OH exchangeable with D_2_O). Mass spectrum, *m*/*z* (*I*_r_ %): 427 (M^+^, 66), 381 (100), 150 (18), 306 (33), 252 (26), 208 (28), 174 (56), 159 (13), 144 (46), 132 (19), 117 (12), 90 (53), 77 (42), 64 (16). Anal. calcd. for C_22_H_13_N_5_O_5_ (427.37); C, 61.83; H, 3.07; N, 16.39%. Found: C, 61.75; H, 3.02; N, 16.23%.

#### 2.1.4. 4-Hydroxy-3-(4-hydroxy-5-nitro-3-oxo-2,3-dihydro-1*H*-pyrazolo[3,4-*b*]pyridin-6-yl)-1-methylquinolin-2(1*H*)-one (5)

**Scheme 3:**
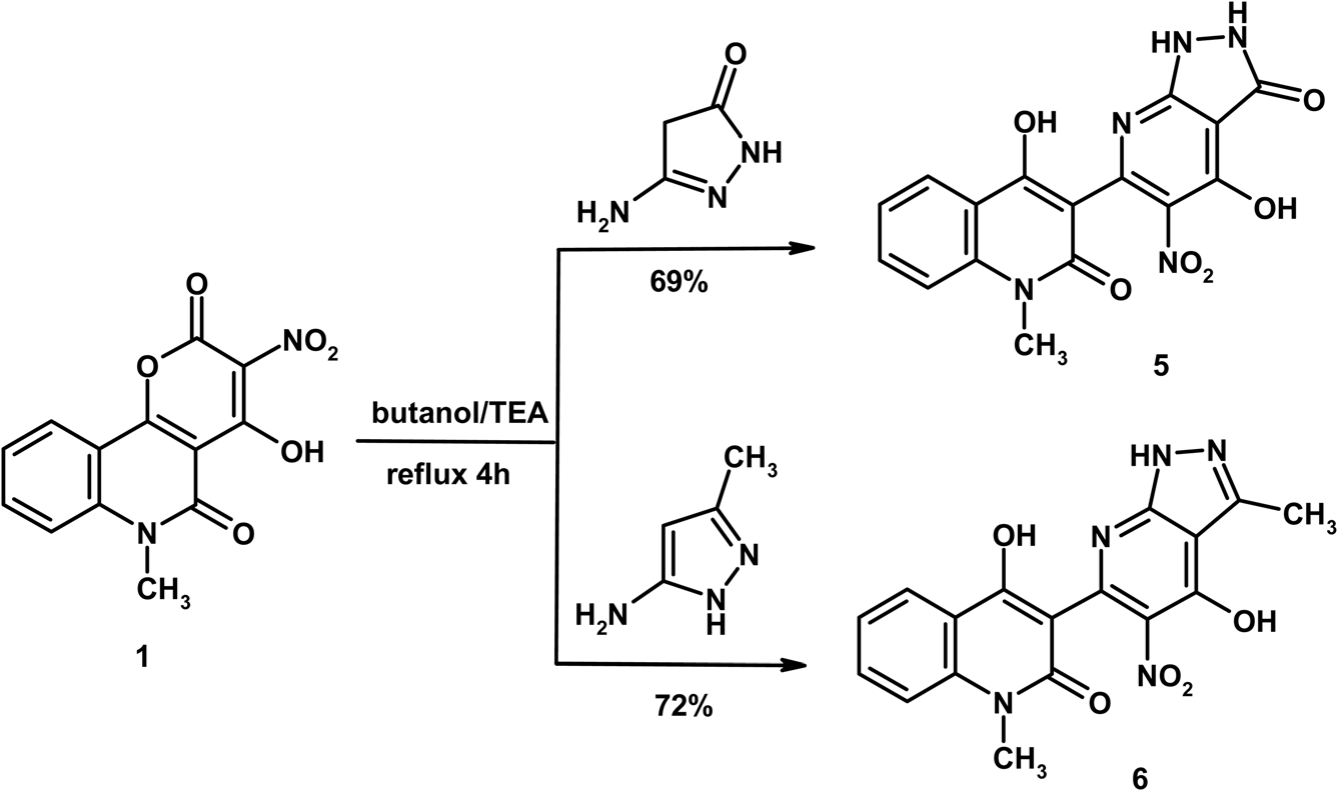
Formation of pyrazolo[3,4-*b*]pyridine derivatives 5 and 6.

As shown in Scheme 3, a mixture of compound 1 (0.58 g, 2 mmol) and 5-amino-2,4-dihydro-3*H*-pyrazol-3-one (0.20 g, 2 mmol) in butanol (10 mL) containing TEA (0.1 mL) was heated under reflux for 4 h. The solid obtained during heating was filtered and crystallized from DMF/H_2_O to give compound 5 as yellow crystals, yield (0.51 g, 69%), m.p. > 300 °C. IR (KBr, cm^−1^): 3446 (OH), 3202, 3179 (2NH), 3055 (CH_arom_), 2966, 2935 (CH_aliph_), 1678 (C=O_pyrazonone_), 1657 (C=O_quinolone_), 1614 (C=N) and 1574 (C=C). ^1^H NMR (DMSO-d_6_, *δ*, 400 MHz): 3.62 (s, 3H, CH_3_), 7.24 (t, 1H, *J* = 8.0 Hz, H-6), 7.45 (d, 1H, *J* = 8.0 Hz, H-8), 7.64 (t, 1H, *J* = 8.0 Hz, H-7), 8.21 (d, 1H, *J* = 8.0 Hz, H-5), 12.18 (bs, 1H, NH_pyrazole_ exchangeable with D_2_O), 12.58 (bs, 1H, NH_pyrazole_ exchangeable with D_2_O), 13.12 (bs, 1H, OH exchangeable with D_2_O), 13.67 (bs, 1H, OH exchangeable with D_2_O). Anal. calcd. for C_16_H_11_N_5_O_6_ (369.29); C, 52.04; H, 3.00; N, 18.96%. Found: 51.86; H, 2.93; N, 18.96%.

#### 2.1.5. 4-Hydroxy-3-(4-hydroxy-3-methyl-5-nitro-1*H*-pyrazolo[3,4-*b*]pyridin-6-yl)-1-methylquinolin-2(1*H*)-one (6)

As shown in Scheme 3, a mixture of compound 1 (0.58 g, 2 mmol) and 5-amino-3-methyl-1*H*-pyrazole (0.20 g, 2 mmol) in butanol (10 mL) containing TEA (0.1 mL) was heated under reflux for 4 h. The solid obtained during heating was filtered and crystallized from AcOH to give compound 6 as yellow crystals, yield (0.53 g, 72%), m.p. 276–277 °C. IR (KBr, cm^−1^): 3406 (OH), 3332 (NH), 3046 (CH_arom_), 2955, 2927 (CH_aliph_), 1645 (C=O_quinolone_), 1616 (C=N), 1575 (C=C). ^1^H NMR (DMSO-d_6_, *δ*, 400 MHz): 2.25 (s, 3H, CH_3pyrazole_), 3.64 (s, 3H, CH_3_), 7.42 (t, 1H, *J* = 7.2 Hz, H-6), 7.67 (d, 1H, *J* = 7.2 Hz, H-8), 7.85 (t, 1H, *J* = 7.2 Hz, H-7), 8.13 (d, 1H, *J* = 7.2 Hz, H-5), 11.28 (bs, 1H, NH_pyrazole_ exchangeable with D_2_O), 12.33 (bs, 1H, OH exchangeable with D_2_O), 12.61 (bs, 1H, OH exchangeable with D_2_O). Mass spectrum, *m*/*z* (*I*_r_ %): 367 (M^+^, 100), 337 (38), 292 (31), 251 (26), 209 (23), 195 (38), 175 (44), 159 (14), 133 (12), 105 (28), 91 (57), 77 (41), 64 (20). Anal. calcd. for C_17_H_13_N_5_O_5_ (367.32); C, 55.59; H, 3.57; N, 19.07%. Found: C, 55.46; H, 3.51; N, 18.93%.

#### 2.1.6. 5-Hydroxy-7-(4-hydroxy-1-methyl-2-oxo-1,2-dihydroquinolin-3-yl)-6-nitropyrido[2,3-*d*] pyrimidine-2,4(1*H*,3*H*)-dione (7)

**Scheme 4:**
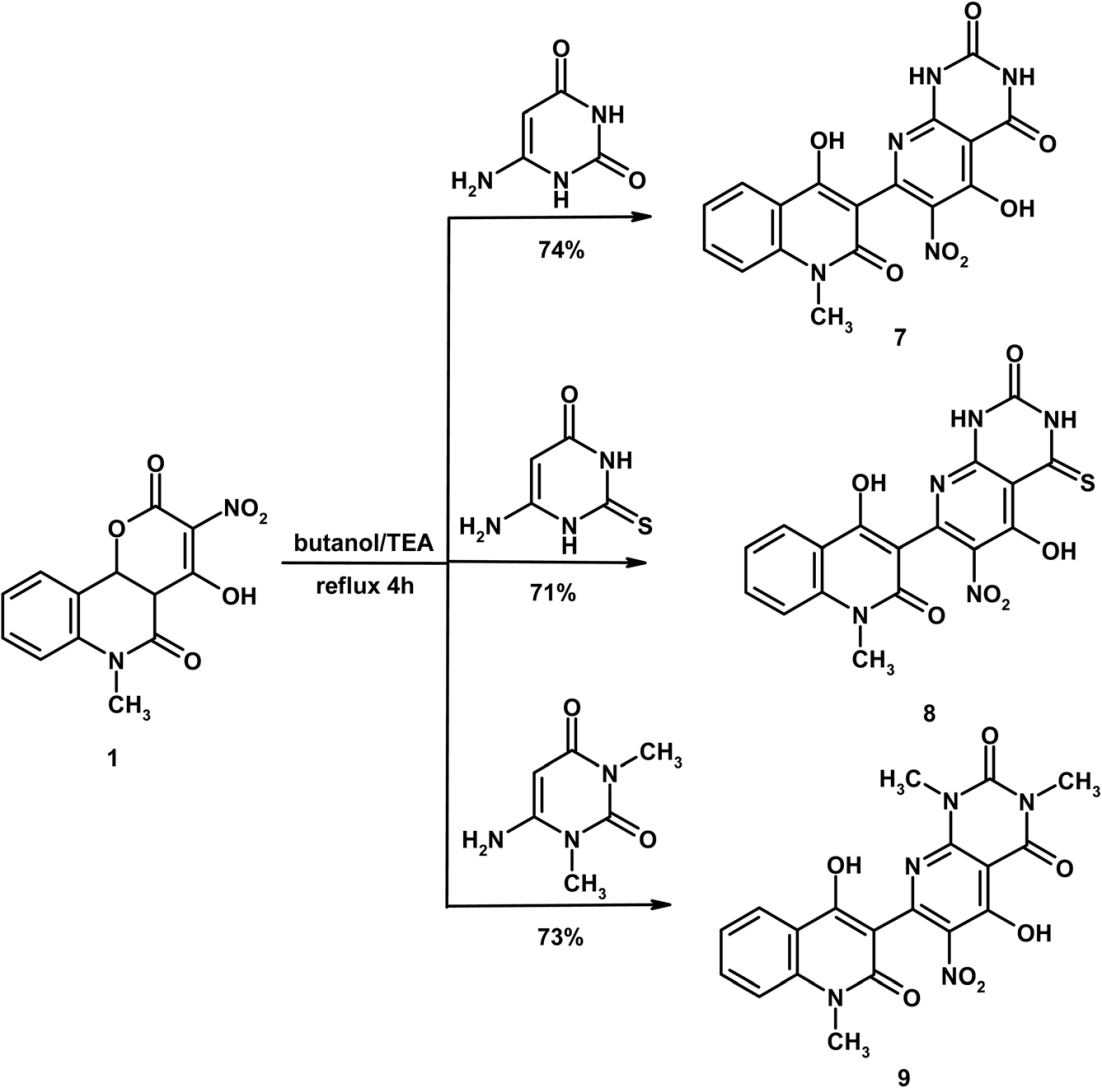
Formation of pyrido[2,3-*d*]pyrimidines 7–9.

As shown in Scheme 4, a mixture of compound 3 (0.58 g, 2 mmol) and 6-aminopyrimidine-2,4(1*H*,3*H*)-dione (0.25 g, 2 mmol) in butanol (10 mL) containing TEA (0.1 mL) was heated under reflux for 4 h. The solid obtained during heating was filtered and crystallized from DMF to give compound 7 as yellow crystals, yield (0.59 g, 75%), m.p. > 300 °C. IR (KBr, cm^−1^): 3445 (OH), 3242, 3218 (2NH), 3045 (CH_arom._), 2983, 2948 (CH_aliph._), 1685 (C=O_pyrimidine_), 1667 (C=O_pyrimidine_), 1648 (C=O_quinolone_), 1619 (C=N), 1567 (C=C). ^1^H NMR (DMSO-d_6_, *δ*, 400 MHz): 3.53 (s, 3H, CH_3_), 7.23 (t, 1H, *J* = 7.2 Hz, H-6), 7.46 (d, 1H, *J* = 7.2 Hz, H-8), 7.62 (t, 1H, *J* = 7.2 Hz, H-7), 8.15 (d, 1H, *J* = 7.2 Hz, H-5), 10.09 (bs, 2H, 2NH_pyrimidine_ exchangeable with D_2_O), 12.78 (bs, 1H, OH exchangeable with D_2_O), 13.39 (bs, 1H, OH exchangeable with D_2_O). Mass spectrum, *m*/*z* (*I*_r_ %): 397 (M^+^, 100), 369 (69), 326 (38), 298 (27), 253 (30), 223 (43), 195 (24), 174 (39), 150 (28), 133 (31), 104 (17), 92 (57), 77 (33), 64 (16). Anal. calcd. for C_17_H_11_N_5_O_7_ (397.30); C, 51.39; H, 2.79; N, 17.63%. Found: C, 51.18; H, 2.52; N, 17.58%.

#### 2.1.7. 5-Hydroxy-7-(4-hydroxy-1-methyl-2-oxo-1,2-dihydroquinolin-3-yl)-6-nitro-4-thioxo-3,4-dihydropyrido[2,3-*d*]pyrimidin-2(1*H*)-one (8)

As shown in Scheme 4, a mixture of compound 3 (0.58 g, 2 mmol) and 6-amino-2-thioxo-2,3-dihydropyrimidin-4(1*H*)-one (0.29 g, 2 mmol) in butanol (10 mL) containing TEA (0.1 mL) was heated under reflux for 4 h. The solid obtained after cooling was filtered and crystallized from DMF/H_2_O to give compound 8 as yellow crystals, yield (0.59 g, 71%), m.p. 289–290 °C. IR (KBr, cm^−1^): 3438 (OH), 3372, 3262 (2NH), 3074 (CH_arom_), 2989, 2928 (CH_aliph_), 1677 (C=O_pyrimidine_), 1651 (C=O_quinolone_), 1613 (C=N), 1571 (C=C). ^1^H NMR (DMSO-d_6_, *δ*, 400 MHz): 3.61 (s, 3H, CH_3_), 7.37 (t, 1H, *J* = 7.6 Hz, H-6), 7.60 (d, 1H, *J* = 7.6 Hz, H-8), 7.78 (t, 1H, *J* = 7.6 Hz, H-7), 8.07 (d, 1H, *J* = 7.6 Hz, H-5), 10.32 (bs, 1H, NH_pyrimidine_ exchangeable with D_2_O), 10.99 (bs, 1H, NH_pyrimidine_ exchangeable with D_2_O), 12.11 (bs, 1H, OH exchangeable with D_2_O), 12.87 (bs, 1H, OH exchangeable with D_2_O). Anal. calcd. for C_17_H_11_N_5_O_6_S (413.36); C, 49.40; H, 2.68; N, 16.94; S, 7.76%. Found: C, 49.23; H, 2.46; N, 16.72; S, 7.65%.

#### 2.1.8. 5-Hydroxy-7-(4-hydroxy-1-methyl-2-oxo-1,2-dihydroquinolin-3-yl)-1,3-dimethyl-6-nitropyrido[2,3-*d*]pyrimidine-2,4(1*H*,3*H*)-dione (9)

As shown in Scheme 4, a mixture of compound 3 (0.58 g, 2 mmol) and 6-amino-1,3-dimethylpyrimidine-2,4(1*H*,3*H*)-dione (0.31 g, 2 mmol) in butanol (10 mL) containing TEA (0.1 mL) was heated under reflux for 4 h. The solid obtained during heating was filtered and crystallized from DMF/H_2_O to give compound 9 as yellow crystals, yield (0.62 g, 73%), m.p. > 300 °C. IR (KBr, cm^−1^): 3446 (OH), 3084 (CH_arom_), 2992, 2953, 2864 (CH_aliph_), 1687 (2C=O_pyrimidine_), 1646 (C=O_quinolone_), 1614 (C=N) and 1577 (C=C). ^1^H NMR (DMSO-d_6_, *δ*, 400 MHz): 3.16 (s, 3H, CH_3_), 3.47 (s, 3H, CH_3_), 3.63 (s, 3H, CH_3_), 7.39 (t, 1H, *J* = 8.0 Hz, H-6), 7.59 (d, 1H, *J* = 7.6 Hz, H-8), 7.99–8.11 (m, 2H, H-7 and H-5), 12.72 (bs, 1H, OH exchangeable with D_2_O), 13.26 (bs, 1H, OH exchangeable with D_2_O). Anal. calcd. for C_19_H_15_N_5_O_7_ (425.35); C, 53.65; H, 3.55; N, 16.46%. Found: C, 53.39; H, 3.46; N, 16.35%.

### 2.2. Computational methods

The GAUSSIAN 09 W program was used to do the computational chemistry calculations for the produced compounds using DFT with B3LYP level at standard 6-311G++(d,p). This method is appropriate for assessing the stability and reactivity of molecules similar to those studied in the present work.^31–33^ Gauss View 5.0 was used to make visual presentations.^34,35^ During the geometry optimization process, there were no restrictions on symmetry. Molecule electrostatic potential (MEP), and optimized geometries associated with MOs energies were among the outcomes of quantum chemistry computations. Moreover, using the 6-311++G(d,p) basis set and the Gauge-Invariant Atomic Orbital (GIAO) technique,^36^ the chemical shift calculations of ^1^H and ^13^C NMR were performed at the B3LYP level as well as these results were compared with experimental values. The polarizability, the first hyperpolarizability, and electronic dipole moment were all characterized at the same level of the DFT. The NLO characteristics of the synthesized compounds are calculated using quantum chemistry. The NLO computations were performed in gas phase at level B3LYP/6-311++G(d,p).^37^

### 2.3. Biological evaluation

#### 2.3.1. Antitumor activity

The antitumor activity of the synthesized compounds was tested against HepG2 cells using the published procedure of evaluating the effect of the test samples on cell morphology and viability.^38–40^

#### 2.3.2. ADME analysis

Traditionally, drug development has included expensive and high-risk stages, such as cellular, animal, and human clinical trials. Therefore, there is a growing trend toward utilizing computer-assisted evaluations to assess the effectiveness of potent compounds and their ability to interact with biological targets. One of these programm is SwissADME (http://www.swissadme.ch)^41^ which is specifically designed to assess ADME parameters for drug candidates. Where, ADME (absorption, distribution, metabolism, and excretion) studies was utilized to predict the physicochemical descriptors, and drug-likeness of all the compounds.^42^ Selected Lipinski,^43^ Ghose,^44^ and Veber,^45^ criteria were employed to forecast the compounds' drug-likeness behavior.

#### 2.3.3. Molecular docking study

The studied compounds underwent molecular docking simulations using AutoDock Vina^46^ to explore their binding interactions with the crystal structure of Human topoisomerase II beta in complex with DNA and amacrine (PDB ID: 4G0U).^47^ The three-dimensional structure of this protein complex was obtained from the Protein Data Bank (PDB) (https://www.rcsb.org/structure/4G0U). Prior to docking, the receptor structure (PDB ID: 4G0U) was prepared by removing excess water molecules, ligands, and heteroatoms, followed by the addition of polar hydrogen atoms and Kollman charges.^48^ The synthesized compounds were prepared as previously reported.^49^

These ligands were then converted to PDBQT format. A grid box was defined for the 4G0U receptor with coordinates (*X* = 32.127, *Y* = 30.108, *Z* = 10.345) and dimensions (*X* = 14.687 Å, *Y* = 12.254 Å, *Z* = 22.254 Å). The binding affinities of the compounds were subsequently evaluated based on their binding energies (kcal mol^−1^).^50^ This computational study offers a comprehensive analysis of the interactions between the ligands and the 4G0U receptor.

## 3. Results and discussion

### 3.1. Characterization of the synthesized compounds

The starting substrate 1 possess an electron deficient α-pyrone ring which structure is expected to generate potentially interesting interactions with nucleophilic reagents. To the best of our knowledge, substrate 1 did not reacted previously with carbon nucleophiles. Consequently, we are motivated to investigate its chemical behavior towards some carbon nucleophiles.

Reaction of 4-hydroxy-6-methyl-3-nitro-2*H*-pyrano[3,2-*c*]quinoline-2,5(6*H*)-dione (1) with cyanoacetamide, in boiling butanol containing TEA, afforded the novel pyridine-3-carbonitrile derivative 2 (Scheme 1).^51,52^ The reaction initially proceeds through deprotonation of cyanoacetamide followed by nucleophilic attack at C-2 position of compound 1 (intermediate A) with subsequent α-pyrone ring opening (intermediate B), cylocondensation (intermediate C) and proton transfer. The mass spectrum for compound 2 exhibited the parent ion peak, as the base peak, at *m*/*z* 354, which is coincident well with the suggested molecular formula C_16_H_10_N_4_O_6_ (354.27). Its IR spectrum presented characteristic absorption bands at *ν* 3445 (OH), 3248 (NH), 2224 (C≡N), 1667 (C=O_pyridone_) and 1639 cm^−1^ (C=O_quinolone_). The ^1^H NMR spectrum presented definite singlet in the upfield region due to NCH_3_ protons at *δ* 3.68, in addition to four aromatic protons at *δ* 7.40 (triplet, H-6), 7.62 (doublet, H-8), 7.81 (triplet, H-7) and 8.11 (doublet, H-5). The spectrum also revealed D_2_O-exchangeable signals in the downfield region at *δ* 11.34, 12.32 and 12.61 due to NH and 2OH protons, respectively.

Similar to the previous mechanism, reaction of compound 1 with 2-aminoprop-1-ene-1,1,3-tricarbonitrile and 1*H*-benzimidazol-2-ylacetonitrile, in refluxing butanol/TEA, furnished 4-hydroxyquinolinone linked pyridine-3-carbonitrile 3 and pyrido[1,2-*a*]benzimidazole-4-carbonitrile 4, respectively (Scheme 2). The IR spectrum of compound 3 presented distinctive absorption bands due to 3C≡N functions at *ν* 2201, 2194 and 2174, in addition to C=O_quinolone_ at *ν* 1651 cm^−1^. While the IR spectrum of compound 4 present definite absorption bands due to C≡N, C=O_quinolone_ and C=N at *ν* 2211, 1647 and 1610 cm^−1^. Structure 4 was further established through mass spectrum which presented the molecular ion peak at *m*/*z* 427 which matches well with the assigned molecular formula C_22_H_13_N_5_O_5_ (427.37).

Then, the reactivity of compound 1 was examined towards some pyrazoles including activated nucleophilic centers such as 5-amino-2,4-dihydro-3*H*-pyrazol-3-one and 5-amino-3-methyl-1*H*-pyrazole giving the corresponding 4-hydroxy-1-methylquinolin-2(1*H*)-one tethered pyrazolo[3,4-*b*]pyridines 5 and 6 (Scheme 3). Specific absorption bands were seen in the IR spectrum of compound 5 at *ν* 3202, 3179 (2NH), 1678 (C=O_pyrazonone_) and 1657 cm^−1^ (C=O_quinolone_), while the spectrum of compound 6 displayed absorption bands at *ν* 3332 (NH) and 1645 cm^−1^ (C=O_quinolone_). Structure 6 was also confirmed by mass spectrum which recorded the parent ion peak at *m*/*z* 367 that coincided well with the proposed formula weight C_17_H_13_N_5_O_5_ (367.32). The ^1^H NMR spectrum of compound 6 showed distinctive singlet signals in the aliphatic region at *δ* 2.25 (CH_3pyrazole_) and 3.64 (CH_3quinoline_), as well as three D_2_O-vanished signals at *δ* 11.28 (NH_pyrazole_), 12.33 (OH) and 12.61 (OH).

After that, the behavior of compound 1 was investigated towards some uracils namely 6-aminouracil, 6-aminothiouracil and 6-amino-1,3-dimethyluracil producing the novel 4-hydroxy 1-methylquinolin-2(1*H*)-one tethered pyrido[2,3-*d*]pyrimidines 7–9, respectively (Scheme 4). The mass spectrum of compound 7 displayed the molecular ion peak, as the base peak, at *m*/*z* 397 supporting the identity of the suggested molecular formula C_17_H_11_N_5_O_7_ (397.30). Its ^1^H NMR spectrum displayed the NCH_3_ protons as typical singlet at *δ* 3.53, meanwhile the aromatic protons appeared the expected splitting patterns as triplet (*δ* 7.23), doublet (*δ* 7.46), triplet (*δ* 7.62) and doublet (*δ* 8.15). Three exchangeable signals were recorded at *δ* 10.09 (2NH_pyrimidine_), 12.78 (OH) and 13.39 (OH).

### 3.2. Theoretical studies

#### 3.2.1. Frontier molecular orbital energies and chemical reactivity

**Fig. 1:**
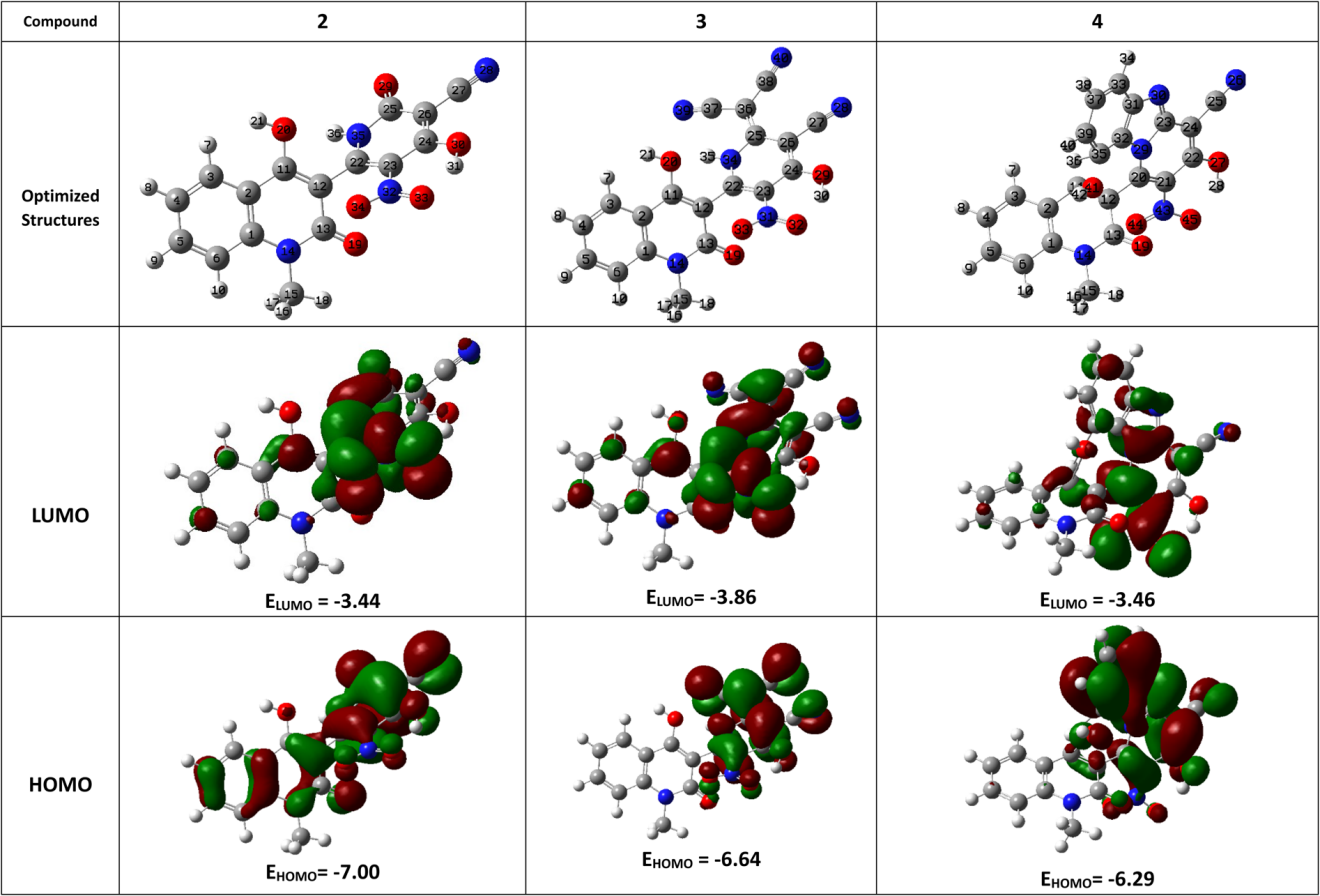
Molecular modeling and the electron density of HOMO and LUMO of compounds 2–4.

Frontier molecular orbitals (FMOs) have a significant impact on the compound's optical, electrical, reactivity, and chemical stability.^53,54^ Compound optimization was carried out with the at the DFT/B3LYP/311++G(d,p) level of theory. Fig. 1, S1 and S2† display the HOMO, LUMO, optimal structures of the produced compounds.^55^

The lowest unoccupied molecular orbital (LUMO) and the highest occupied molecular orbital (HOMO) make up these FMOs. The ability of a compound to give electrons is shown by its *E*_HOMO_ value, whereas a component's *E*_LUMO_ value shows its capacity to receive electrons (Table 1).

**Table 1:** Calculated chemical reactivity descriptors of compounds 1–9

Compound	*E*_T_ (au)	*E*_HOMO_ (eV)	*E*_LUMO_ (eV)	IP (eV)	EA (eV)	Δ*E* (eV)	*χ* (eV)	*μ* (eV)	*η* (eV)	*S* (eV^−1^)	*ω* (eV)	*ε* (eV^−1^)	Δ*N*
1	−1061.27	−7.042	−3.418	7.042	3.418	3.624	5.230	−5.230	1.812	0.552	7.547	0.133	2.886
2	−1286.35	−7.003	−3.437	7.003	3.437	3.566	5.220	−5.220	1.783	0.561	7.642	0.131	2.928
3	−1434.92	−6.647	−3.864	6.647	3.864	2.783	5.255	−5.255	1.391	0.719	9.925	0.101	3.777
4	−1496.35	−6.286	−3.457	6.286	3.457	2.829	4.871	−4.871	1.414	0.707	8.390	0.119	3.444
5	−1341.71	−6.563	−3.008	6.563	3.008	3.555	4.785	−4.785	1.778	0.563	6.440	0.155	2.692
6	−1305.80	−6.392	−2.822	6.392	2.822	3.570	4.607	−4.607	1.785	0.560	5.944	0.168	2.581
7	−1455.15	−6.625	−2.716	6.625	2.716	3.909	4.671	−4.671	1.955	0.512	5.581	0.179	2.389
8	−1778.11	−6.646	−3.071	6.646	3.071	3.575	4.859	−4.859	1.788	0.559	6.603	0.151	2.718
9	−1533.79	−6.550	−2.554	6.550	2.554	3.995	4.552	−4.552	1.998	0.501	5.186	0.193	2.279

Energy gap (Δ*E*, eV) is a measure of a molecule's chemical stability and reactivity; a larger Δ*E* suggests higher stability. The compound 9 has a Δ*E* of 3.995 eV, which is the largest value. This implies that the compound 9 is more stable than other compounds.

Additionally, the quantum chemical parameters as chemical potential (*μ*), electronegativity (*χ*), chemical hardness (*η*), softness (*S*), electrophilicity (*ω*), nucleophilicity (*ε*), and additional electronic charge (Δ*N*_max_) were calculated using the following equations^56–58^

1 *I* = −*E*_HOMO_

2 *Y* = −*E*_LUMO_

3
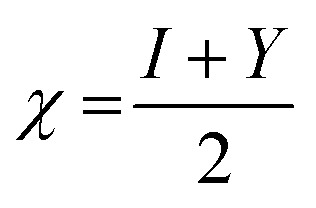
4
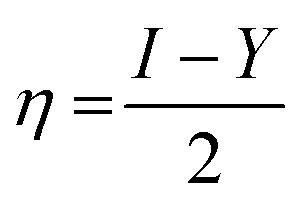
5
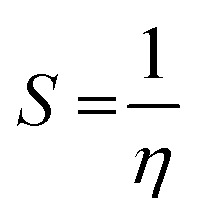
6
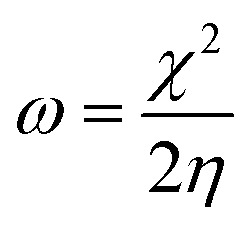
7
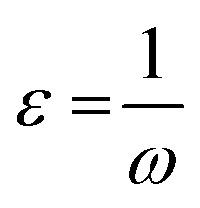

8 *μ* = −*χ*

9
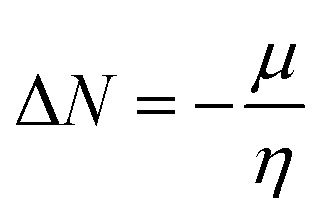


The energy required to remove an electron from a component is indicated by the ionization potential (*I*, eV).^50^ The energy shift that occurs when an electron is added to a component is measured by electron affinity (*Y*, eV). In addition, chemical resistance to shifts in electron distribution is measured by hardness (*η*, eV), whereas chemical softness (*S*, eV^−1^) is the opposite of hardness (*η*).^59^ Compound 9 has a hardness value (*η*) of 1.998 eV which is the hardest compound, hence the less reactive one and comparatively hard. On the other hand, compound 3 has a softness value (*S*) of 0.719 eV ^−1^ and therefore the more reactive and softer than the others. A molecule's propensity to draw electrons is indicated by its electronegativity (*χ*, eV).^60^ Compared to other compounds, compound 3 has a greater *χ* of 5.255 eV. Further, the tendency of electrons to escape from a molecule is indicated by chemical potentials (*μ*, eV). The prepared compounds have a negative chemical potential, indicating their stability. The ability of a molecule to accept electrons is measured by its electrophilicity index (*ω*, eV). Compound 3 exhibits a stronger electrophile than the others, as evidenced by its *ω* value of 9.925 eV. A component's ability to donate electrons is known as its nucleophilicity (*ε*, eV^−1^).^61^ Compared to other compounds, compound 7 has a greater *ε* of 0.179 eV^−1^, so the strongest nucleophile.

#### 3.2.2. ^1^H NMR and ^13^C NMR spectroscopy

One promising approach to study the structures of organic molecules is to combine quantum computational chemistry techniques with nuclear magnetic resonance (NMR) spectroscopy. The GIAO method with B3LYP/6-11++G basis set has also been used to calculate theoretically the chemical shift of ^1^H and ^13^C NMR of the produced compounds.^62,63^Tables 2 and 3 exhibit the theoretical and experimental chemical shifts for the ^1^H NMR spectral results, and Fig. S12, S14, S17, S19, S22, S25, S27 and S29† display the ^1^H NMR spectra in DMSO.

**Table 2:** Calculated and experimental ^1^H NMR chemical shifts of compounds 2–5 on B3LYP/6-311 G(d,p) basis set

Compound 2			Compound 3			Compound 4			Compound 5		
Atoms	Calculated	Experimental	Atoms	Calculated	Experimental	Atoms	Calculated	Experimental	Atoms	Calculated	Experimental
17-H	1.58	3.68	17-H	1.63	3.54	17-H	3.16	3.62	16-H	2.92	3.62
18-H	2.76	3.68	18-H	2.77	3.54	16-H	3.18	3.62	17-H	2.94	3.62
16-H	3.41	3.68	16-H	3.49	3.54	18-H	3.45	3.62	18-H	3.80	3.62
8-H	7.57	7.4	8-H	7.51	7.13	40-H	6.53	7.23	8-H	7.62	7.24
7-H	7.61	7.62	7-H	7.54	7.33	34-H	7.48	7.26	10-H	7.63	7.45
9-H	7.927	7.81	9-H	7.91	7.54	8-H	7.49	7.34	7-H	7.76	7.64
10-H	8.18	8.11	10-H	8.13	8.14	38-H	7.58	7.39	9-H	8.04	8.21
36-H	10.11	11.34	35-H	9.85	11.46	36-H	7.68	7.62	37-H	11.58	12.18
31-H	13.29	12.32	30-H	12.99	12.39	10-H	7.74	7.69	35-H	11.69	12.58
21-H	13.57	12.61	21-H	13.05	12.79	7-H	7.74	8.04	28-H	12.78	13.12
						9-H	8.09	8.21	21-H	13.01	13.67
						42-H	12.52	12.59			
						28-H	13.44	13.31			

**Table 3:** Calculated and experimental ^1^H NMR chemical shifts of compounds 6–9 on B3LYP/6-311++G(d,p) basis set

Compound 6			Compound 7			Compound 8			Compound 9		
Atoms	Calculated	Experimental	Atoms	Calculated	Experimental	Atoms	Calculated	Experimental	Atoms	Calculated	Experimental
39-H	2.69	2.25	16-H	2.95	3.53	16-H	2.95	3.61	40-H	2.88	3.16
37-H	2.92	2.25	17-H	2.97	3.53	17-H	2.96	3.61	17-H	2.94	3.63
16-H	2.93	3.64	18-H	3.90	3.53	18-H	4.04	3.61	16-H	2.96	3.63
17-H	2.94	3.64	8-H	7.53	7.23	10-H	7.56	7.37	42-H	2.98	3.16
38-H	2.95	2.25	10-H	7.55	7.46	8-H	7.57	7.6	44-H	3.193	3.47
18-H	5.08	3.64	7-H	7.83	7.62	7-H	7.80	7.78	45-H	3.19	3.47
8-H	7.63	7.42	9-H	7.92	8.15	9-H	7.94	8.07	46-H	3.45	3.47
10-H	7.64	7.67	38-H	10.43	10.09	35-H	10.48	10.32	41-H	3.49	3.16
7-H	7.78	7.85	35-H	10.46	10.09	38-H	11.05	10.99	18-H	3.91	3.63
9-H	8.04	8.13	27-H	12.09	12.78	21-H	12.31	12.11	8-H	7.51	7.39
35-H	10.43	11.28	21-H	13.08	13.39	27-H	13.63	12.87	10-H	7.52	7.59
21-H	12.92	12.33							7-H	7.83	7.99
28-H	13.20	12.61							9-H	7.86	8.11
									21-H	12.08	12.72
									27-H	12.92	13.26

In compound 2, the experimental ^1^H NMR chemical shift of the two OH groups were found at *δ* 12.32 and 12.61 ppm, while the computed signals were determined at *δ* 13.29 and 13.57 ppm. The ^1^H NMR signals of benzene ring of compound 3 were recorded experimentally in the range *δ* 7.13–8.14 ppm, whereas theoretically calculated at *δ* 7.51–8.13 ppm. The ^1^H NMR chemical shift of OH protons of compound 4 were observed experimentally at *δ* 12.59 and *δ* 13.31 ppm, while computed at *δ* 12.52 and 13.44 ppm.

In compound 5, the two singlet signals for NH protons were calculated at *δ* 11.58 and 11.69 ppm, while the signals in the experimental spectrum were identified at *δ* 12.18 and 12.58 ppm. The calculated chemical shifts of methyl protons were computed at *δ* 2.69, 2.92 and 2.95 ppm; showing good agreement with the experimental value at *δ* 2.25 ppm (singlet).

Two signals of NH groups were detected at *δ* 10.44 and 10.46 for compound 7 as well as at *δ* 10.49 and 11.05 ppm for compound 8. These signals were identified experimentally at *δ* 10.09 and 10.09 for compound 7, and at *δ* 10.32 and 10.99 ppm for compound 8. Additionally, ^1^H NMR chemical shift of the two N–Me groups of pyrimidine ring in compound 9 were recorded experimentally at *δ* 3.16 and 3.47 ppm, while their theoretically values were observed in the range *δ* 2.88–3.49 ppm.

**Fig. 2:**
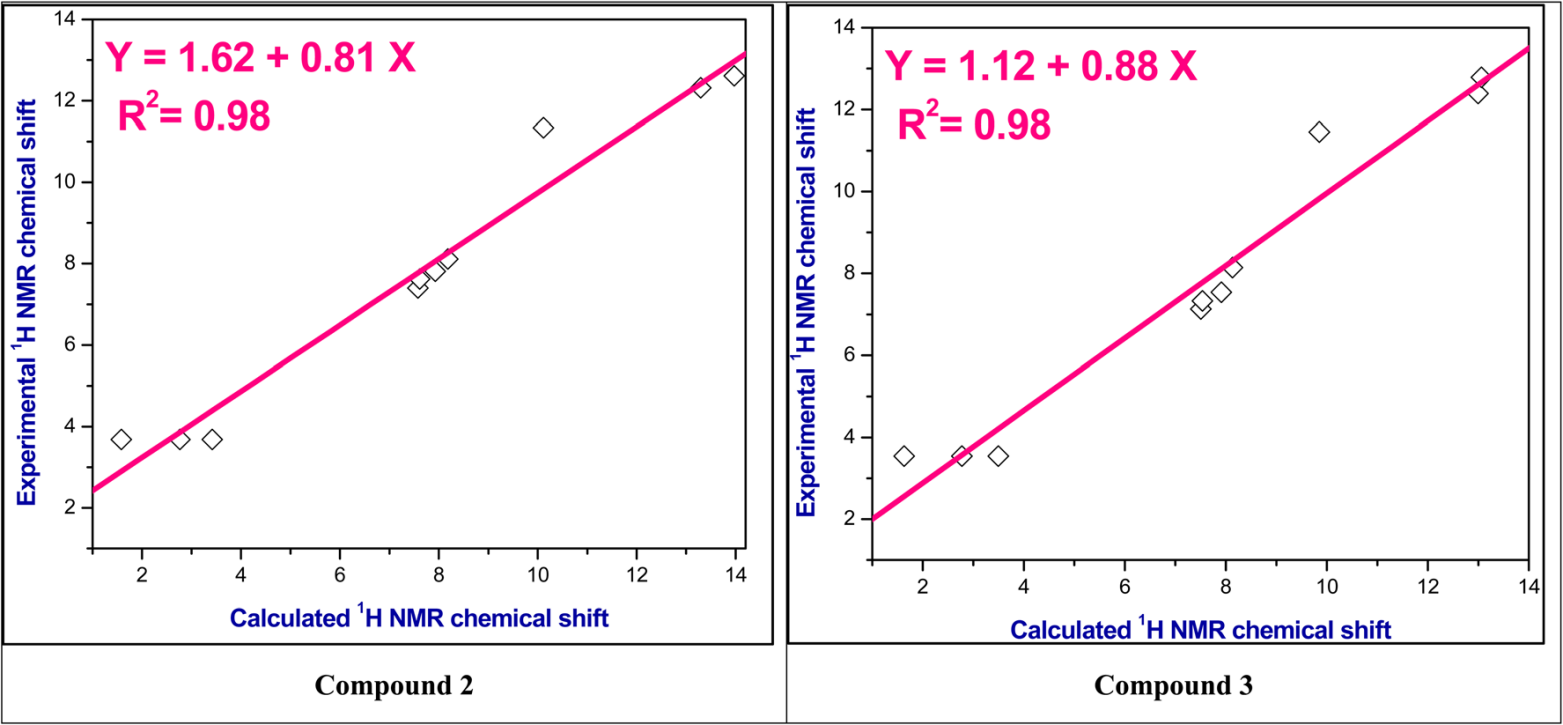
The correlation relationships of the experimental *versus* calculated ^1^H NMR chemical shifts of compounds 2 and 3.

The experimental ^1^H NMR chemical shifts were plotted with the theoretical ^1^H NMR chemical shifts and correlation coefficient (*R*^2^) is found in the range 0.98–0.99 as shown in Fig. 2 and S3–S5.† Thus, these computed values are good agreement with experimental values.

#### 3.2.3. FT-IR vibrational analysis

This analysis holds important significance as it allows for the precise identification of molecules through their unique infrared absorption patterns, enabling researchers to determine the chemical composition of substances with high accuracy.^64^ DFT calculations on harmonic frequencies revealed excellent vibrational frequencies for organic compounds.^65^ The theoretical harmonic frequencies were calculated using B3LYP/6-311++G(d,p) and then adjusted by a classical factor (0.961) to rectify the theoretical error. Tables 4–6 display the theoretical and actual infrared vibrational frequencies of compounds 2–9. The FT-IR spectra (experimental and calculated) are shown in Fig. S10, S13, S15, S18, S20, S23, S26 and S28.†

**Table 4:** Experimental and theoretical wavenumbers and corresponding vibrational assignments of the studied compounds 2–4 at the B3LYP/6-311++G (d,p)

Compound 2			Compound 3			Compound 4		
*υ*_exp._ (cm^−1^)	*υ*_the._ (cm^−1^)	Assignment	*υ*_exp._ (cm^−1^)	*υ*_the._ (cm^−1^)	Assignment	*υ*_exp._ (cm^−1^)	*υ*_the._ (cm^−1^)	Assignment
3445	3414	OH	3440	3477	OH	3445	3420	OH
3248	3321	NH	3281	3362	NH	3042	3173	CH_aromatic_
3028	3178	CH_aromatic_	3014	3061	CH_aromatic_	2981, 2943	3100, 2985	CH_aliphatic_
2966, 2931	3012, 2932	CH_aliphatic_	2982, 2936	3009, 2973	CH_aliphatic_	2211	2218	C≡N
2224	2173	C≡N	2201, 2194, 2174	2214, 2181, 2162	C≡N	1647	1672	C=O_quinolone_
1667	1685	C=O_pyridone_	1651	1656	C=O_quinolone_	1610	1604	C=N
1639	1646	C=O_quinolone_	1594	1596	C=C	1566	1563	C=C
1617	1602	C=C						

**Table 5:** Experimental and theoretical wavenumbers and corresponding vibrational assignments of the studied compounds 5–7 at the B3LYP/6-311++G (d,p)

Compound 5			Compound 6			Compound 7		
*υ*_exp._ (cm^−1^)	*υ*_the._ (cm^−1^)	Assignment	*υ*_exp._ (cm^−1^)	*υ*_the._ (cm^−1^)	Assignment	*υ*_exp._ (cm^−1^)	*υ*_the._ (cm^−1^)	Assignment
3446	3428	OH	3406	3442	OH	3445	3430	OH
3202, 3179	3347, 3282	NH	3332	3297	NH	3242, 3218	3344, 3280	NH
3055	3064	CH_aromatic_	3046	3125	CH_aromatic_	3045	3129	CH_aromatic_
2966, 2935	3036, 2911	CH_aliphatic_	2955, 2927	3032, 2977	CH_aliphatic_	2983, 2948	3023, 2983	CH_aliphatic_
1678	1685	C=O_pyrazolone_	1645	1660	C=O_quinolone_	1685, 1667	1690, 1669	C=O_pyrimidine_
1657	1646	C=O_quinolone_	1616	1606	C=N	1648	1649	C=O_quinolone_
1614	1603	C=N	1575	1592	C=C	1619	1605	C=N
1574	1595	C=C				1567	1583	C=C

**Table 6:** Experimental and theoretical wavenumbers and corresponding vibrational assignments of the studied compounds 8 and 9 at the B3LYP/6-311++G (d,p)

Compound 8			Compound 9		
*υ*_exp._ (cm^−1^)	*υ*_the._ (cm^−1^)	Assignment	*υ*_exp._ (cm^−1^)	*υ*_the._ (cm^−1^)	Assignment
3438	3442	OH	3446	3390	OH
3372, 3262	3365, 3338	NH	3084	3120	CH_aromatic_
3074	3104	CH_aromatic_	2992, 2953, 2864	3067, 3039, 2984	CH_aliphatic_
2989, 2928	3032, 2994	CH_aliphatic_	1687	1690, 1678	C=O_pyrimidine_
1677	1670	C=O_pyrimidine_	1646	1650	C=O_quinolone_
1651	1641	C=O_quinolone_	1614	1622	C=N
1613	1614	C=N	1577	1595	C=C
1571	1576	C=C			

In compound 2, the OH and NH stretching vibrations were recorded experimentally at *ν* 3445 and 3248 cm^−1^, which found theoretically at *ν* 3414 and 3321 cm^−1^, respectively. In addition, the experimental IR spectrum of compound 3 showed three C≡N stretching modes at *ν* 2201, 2194 and 2174 cm^−1^, while the theoretical values were observed at *ν* 2214, 2181 and 2162 cm^−1^. Moreover, the measured values for C≡N and C=O_quinolone_ stretching modes in compound 4 were seen at *ν* 2211 and 1647 cm^−1^, which matched with the theoretical values at *ν* 2218 and 1672 cm^−1^.

Further, in compound 5, the measured result for the stretching vibrations of C=O_pyrazole_ and C=O_quinolone_ were seen at *ν* 1678 and 1657 cm^−1^, whereas the calculated values observed at *ν* 1685 and 1646 cm^−1^, respectively. In compound 6, the experimental findings of the stretching modes showed C=O_quinolone_ at *ν* 1645 cm^−1^, which is consistent with the theoretical value at *ν* 1660 cm^−1^.

Additionally, the stretching vibrations of 2C=O_pyrimidine_ and C=O_quinolone_ in compounds 7 and 9 were found at *ν* 1685/1687, 1667/1687 and 1648/1646 cm^−1^, respectively, which were theoretically predicted at *ν* 1690/1690, 1669/1678 and 1649/1650 cm^−1^. The theoretical stretching values for C=O_pyrimidine_ and C=O_quinolone_ in compound 8 were seen at *ν* 1670 and 1641 cm^−1^, whereas these values were observed at *ν* 1677 and 1651 cm^−1^, respectively.

**Fig. 3:**
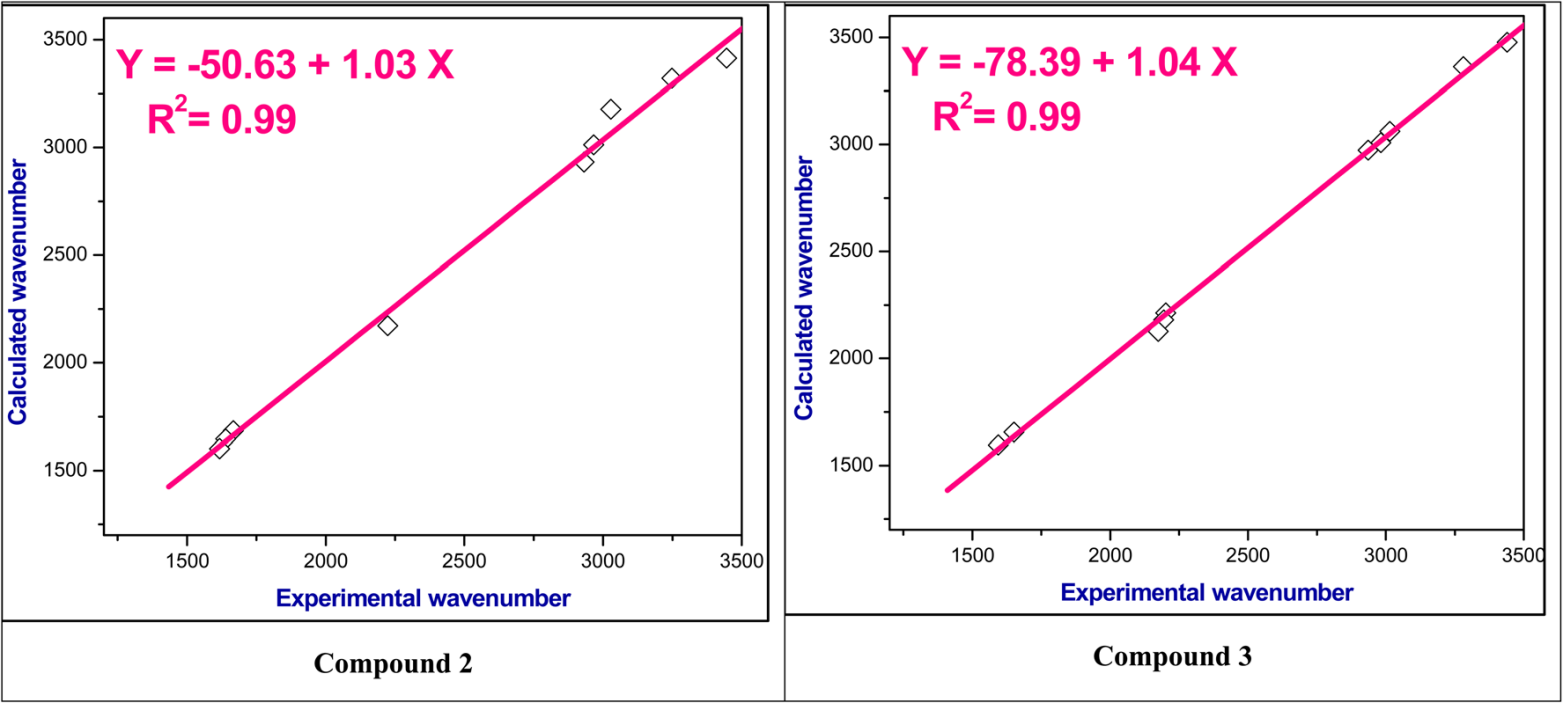
The correlation relationships of the experimental *versus* calculated IR wavenumbers of compounds 2 and 3.

For the studied compounds 2–9, the stretching modes of C=C in the benzene rings were observed in the range *ν* 1566–1617 cm^−1^ which falls within the theoretical spectra in the range at *ν* 1563–1602 cm^−1^. Furthermore, the actual aliphatic C–H stretching vibrations were in the range *ν* 2864–2992 cm^−1^ which consistent with the predicted value of *ν* 3100–2911 cm^−1^. It was found that the aromatic C–H stretching modes had values in the range at *ν* 3014–3084 cm^−1^, whereas the theoretical values found in the range at *ν* 3061–3178 cm^−1^. Fig. 3 and S6–S8† showed a relationship between the wavenumbers derived from experimental observations and those computed theoretically for these functional groups; and exhibit a correlation coefficient (*R*^2^) of 0.99.

#### 3.2.4. Molecular electrostatic potential (MEP)

**Fig. 4:**
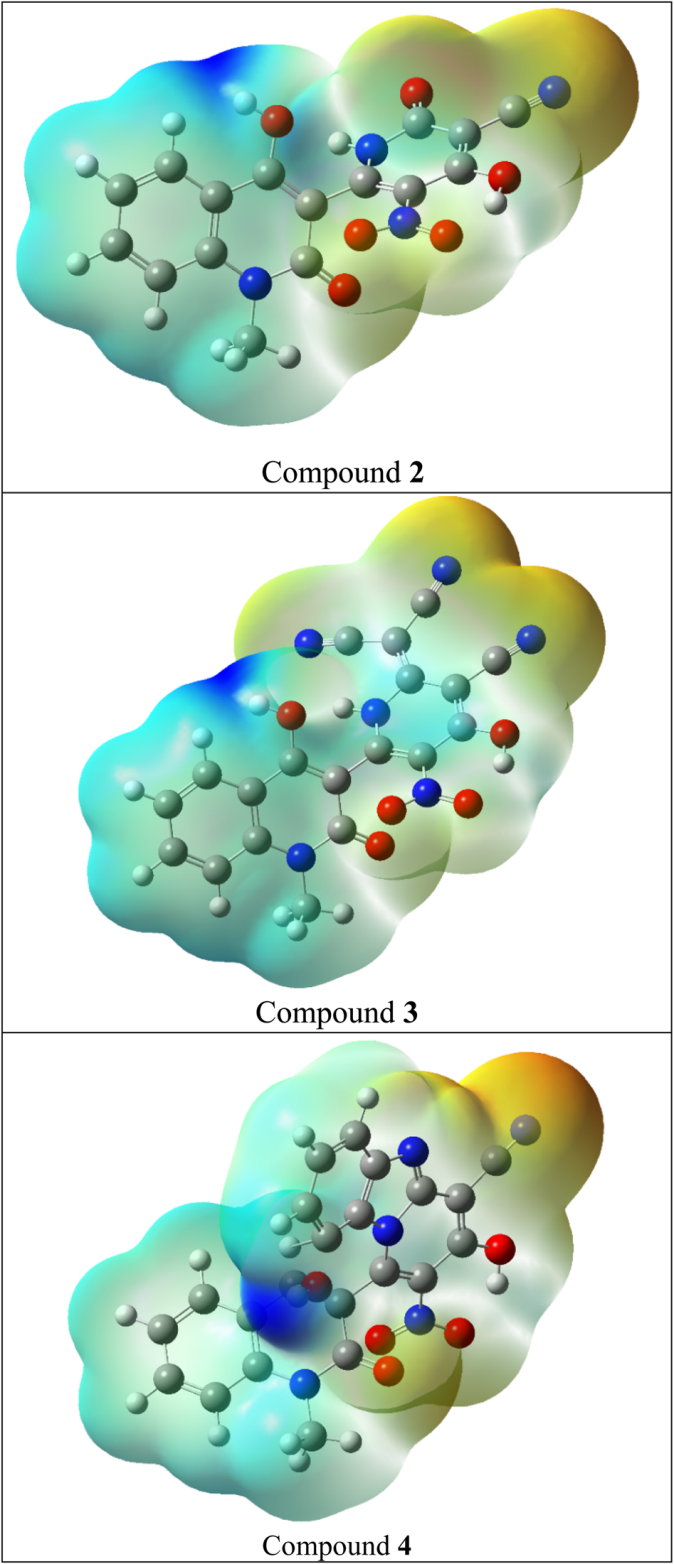
Molecular electrostatic potential of compounds 2–4.

Molecular electrostatic potential (MEP) mapping provides information on molecule's size, hydrogen bonding interactions and net charge distributions (generated by electrons and nuclei). Thus, it predicts the locations of electrophilic attack (electron-rich areas with the highest electronegativity) and nucleophilic attacks (electron-deficient areas with the highest positive electrostatic potential).^66,67^ The electrostatic potential of the molecule was computed by DFT at the B3LYP/6-311G++(d,p) basis set.^68^Fig. 4 and S9† show the MEP mapping for the prepared compounds.

The MEP map displays various electrostatic potentials in color. The electrostatic potential in the MEP map's color scheme is arranged as follows: blue > green > yellow > orange > red. As a result, the blue regions on the map symbolized areas with the strongest attraction (lower electron density), while the red regions showed areas with the strongest repulsion (higher electron density).^69^ Compounds' strong electron-attracting capabilities are indicated by the deep blue representation of their nucleophilic attack sites, which are primarily found on hydrogen and carbon atoms.

On the other hand, areas of significant electron repulsion are shown by the deep yellow and red coloration of the electrophilic attack sites, which are mostly connected to the nitrogen, sulphur and oxygen atoms. Where, the lone pairs of electrons on oxygen, sulphur and nitrogen atoms create areas of negative electrostatic potential that confirm their nucleophilic capabilities.^70^

#### 3.2.5. Nonlinear optical (NLO) effects

The NLO properties of the organic molecules have attracted a lot of attention because of their numerous applications in photonic devices, laser technology, and telecommunications. First hyperpolarizability (*β*_tot_) and associated characteristics including dipole moments (*μ*), polarizability (*α*), and anisotropy of polarizability (Δ*α*) are computed in order to find NLO qualities.^71^Eqn (10)–(13) are used to calculate these parameters as follow^72^

10 *μ* = (*μ*_*x*_^2^ + *μ*_*y*_^2^ + *μ*_*z*_^2^)^1/2^

11 *α* = (*α*_*xx*_ + *α*_*yy*_ + *α*_*zz*_)/3

12 Δ*α* = (2)^−0.5^[(*α*_*xx*_ − *α*_*yy*_)^2^ + (*α*_*yy*_ − *α*_*zz*_)^2^ + (*α*_*zz*_ − *α*_*xx*_)^2^ + 6(*α*_*yz*_)^2^ + 6(*α*_*xy*_)^2^ + 6(*α*_*xz*_)^2^]^0.5^

13 *β*_tot_ = [(*β*_*xxx*_ + *β*_*xyy*_ + *β*_*xzz*_)^2^ + (*β*_*yyy*_ + *β*_*yzz*_ + *β*_*yxx*_)^2^ + (*β*_*zzz*_ + *β*_*zxx*_ + *β*_*zyy*_)^2^]^0.5^

Table 7 displays the outcomes of these computations. The dipole moment (*μ*) of products 1–9 was calculated to fall between 5.59 and 15.55 D. Furthermore, values of first hyperpolarizability of these compounds are 3–18 times higher than urea.^73^ Consequently, these compounds are the most promising and effective NLO materials, suitable materials for NLO applications and attractive objects for additional research into nonlinear optical properties.

**Table 7:** The dipole moment (*μ*), mean polarizability (*α*), anisotropy of the polarizability (Δ*α*) and first-order hyperpolarizability (*β*) for compounds 1–9

Compound no.	*μ*_*x*_	*μ*_*y*_	*μ*_*z*_	*μ*_total_	〈*α*〉 (au)	〈*α*〉 (esu) ×10^−23^	Δ*α* (au)	Δ*α* (esu) ×10^−24^	*β*_total_ (au)	*β*_total_ (esu) ×10^−30^
1	9.14	0.21	−0.001	9.15	−123.51	−1.83	16.85	2.50	203.50	1.76
2	−14.50	−1.08	−1.59	14.63	−158.14	−2.34	40.25	5.97	605.64	5.23
3	15.45	−0.07	−1.73	15.55	−186.52	−2.76	48.38	7.17	816.91	7.06
4	−11.80	4.68	0.19	12.70	−188.40	−2.79	27.34	4.05	553.71	4.78
5	7.32	−5.94	−0.52	9.44	−149.66	−2.22	19.33	2.86	299.15	2.58
6	4.28	−3.56	0.52	5.59	−146.33	−2.17	28.66	4.25	147.27	1.27
7	−5.27	−5.23	−0.75	7.46	−161.68	−2.40	45.35	6.72	244.71	2.11
8	5.75	5.05	−0.91	7.71	−169.19	−2.51	45.09	6.68	316.89	2.74
9	−3.96	−6.28	−0.81	7.47	−171.01	−2.53	52.35	7.76	188.75	1.63

### 3.3. Biological evaluation

#### 3.3.1. Antitumor assay

**Fig. 5:**
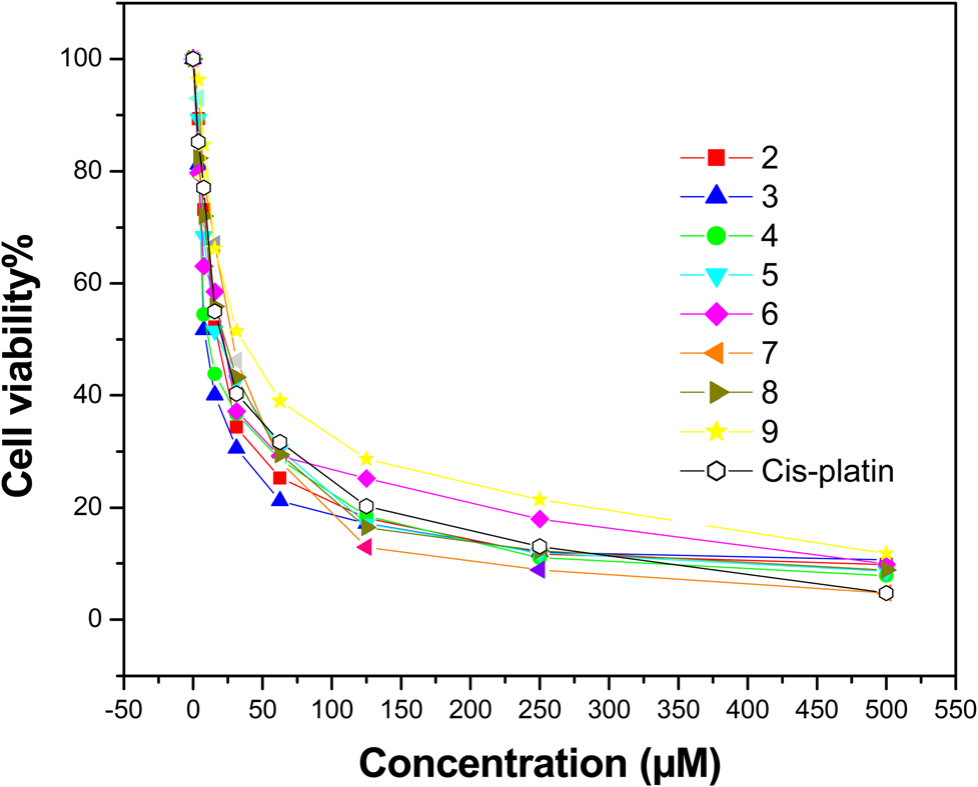
Relation between cell viability and concentration of all synthesized compounds on the proliferation of (HepG2) cell line (cisplatin as a standard drug).

Using MTT colorimetric method, the newly synthesized compounds 2–9 were screened for their anticancer activity against hepatocellular carcinoma (HepG-2) cell lines for 24 hours.^38–40^ The cytotoxicity of cisplatin was also assessed for comparative purposes. The obtained results illustrated that all synthesized compounds displayed a growth inhibition activity on the tested cell line (Fig. 5).

**Fig. 6:**
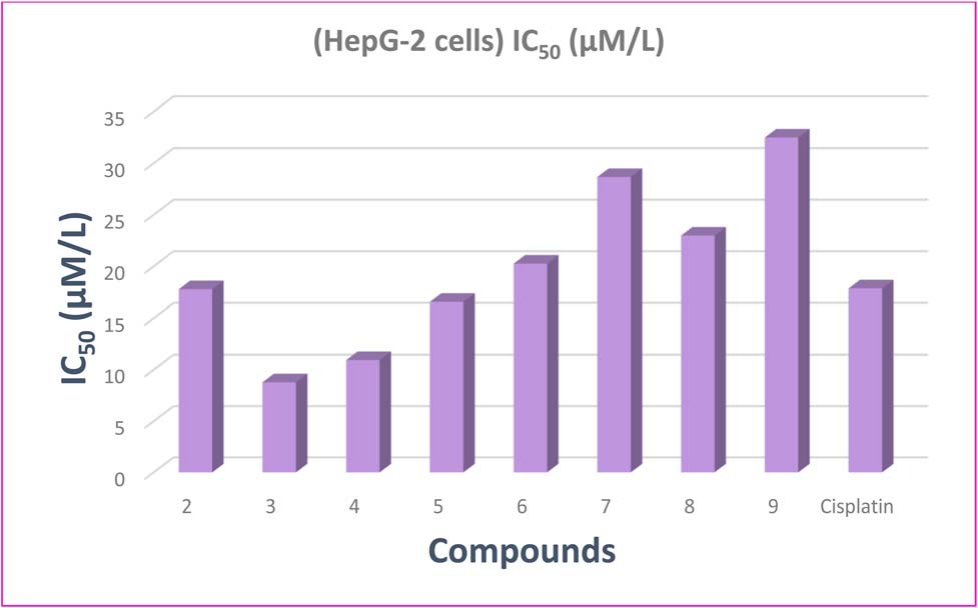
Virtual IC_50_ values of the studied compounds and cisplatin as a standard drug against HepG-2 cell lines.

The results are provided as IC_50_ values and compared with that of cisplatin. The IC_50_ values are summarized in Fig. 6 and Table 8. The target compounds 2–9 showed high to moderate antiproliferative efficacy against hepatocellular carcinoma (HepG-2) cell. Interestingly, compound 3 with IC_50_ 8.79 μM L^−1^ has the strongest potency effect against HepG-2, as well as is more effective than cisplatin with IC_50_ 17.86 μM L^−1^. Additionally, compounds 2, 4 and 5 exerted more potent effect than cisplatin as a reference with IC_50_ values 17.78 ± 0.83, 10.95 ± 0.76 and 16.56 ± 0.80 μM L^−1^, respectively. Finally, compounds 6–9 demonstrated moderate inhibitory effect compared to the positive drug. The investigation into the structural activity relationship (SAR) of the designed compounds focused on their efficacy against the HepG-2 cell line revealed that: high anticancer activity of compounds 2 and 3 may attribute to the presence of pyridine moiety. By substituting oxygen atom of the pyridine ring in compound 2 by propanedinitrile in compound 3 led to more potent activity. Moreover, compound 4 with IC_50_ = 10.95 μM L^−1^ has stronger potency against HepG-2 than cisplatin. This may be due to fusion of pyridine moiety with benzimidazole ring.

**Table 8:** IC_50_ values of compounds 2–9 against hepatocellular carcinoma cells lines (HepG-2) for 24 hours

Compound no.	(HepG-2 cells) IC_50_ (μM L^−1^)
2	17.78 ± 0.83
3	8.79 ± 0.68
4	10.95 ± 0.76
5	16.56 ± 0.80
6	20.26 ± 0.96
7	28.67 ± 1.12
8	22.98 ± 0.99
9	32.49 ± 1.23
Cisplatin	17.86 ± 0.92

Additionally, compounds 5 and 6 have IC_50_ of 16.56 ± 0.80 and 20.26 ± 0.96 μM L^−1^, respectively. This may be attribute to presence of pyrazolo[3,4-*b*]pyridine moieties. Where, compound 5 has similar potency to cisplatin, while compound 6 has moderate activity. Replacing C–Me at position 3 in compound 6 with C=O in compound 5 led to increasing activity against the HepG-2 cell lines. Moreover, pyrido[2,3-*d*]pyrimidines 7–9, in which the pyridine ring fused with pyrimidine moiety, showed moderated potency relative to cisplatin (IC_50_ = 17.86 ± 0.92 μM L^−1^), with IC_50_ values ranging from 22.98 ± 0.99 to 32.49 ± 1.23 μM L^−1^.

**Fig. 7:**
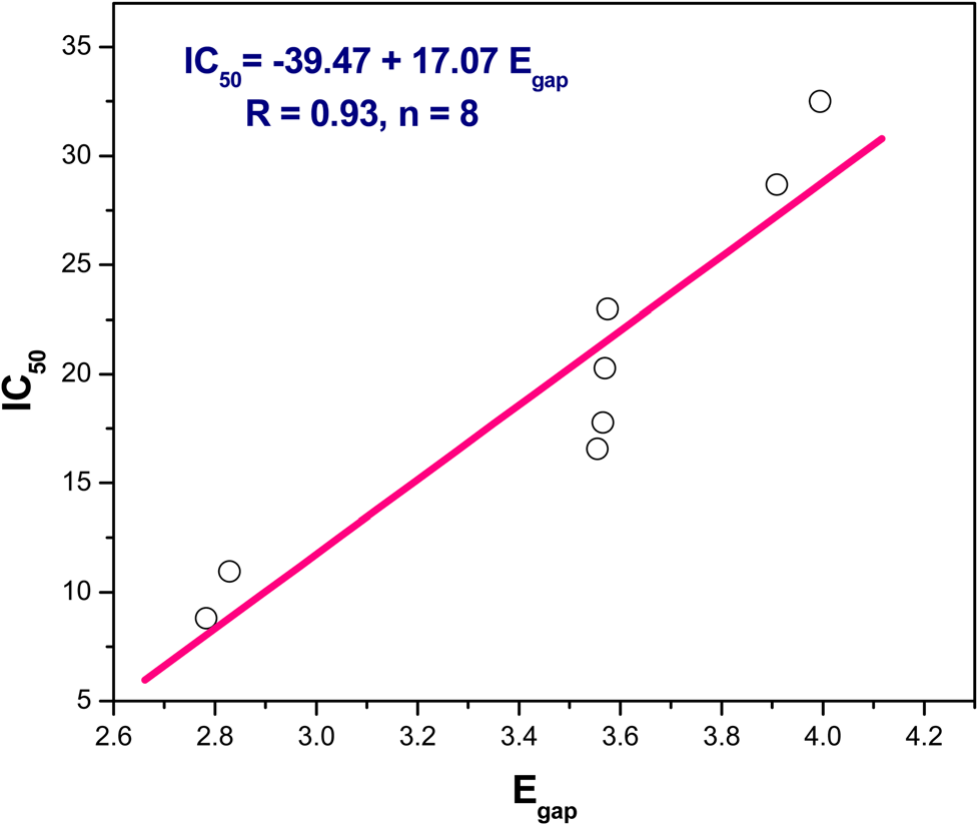
The linear correlation between *E*_gap_*versus* IC_50_.

**Fig. 8:**
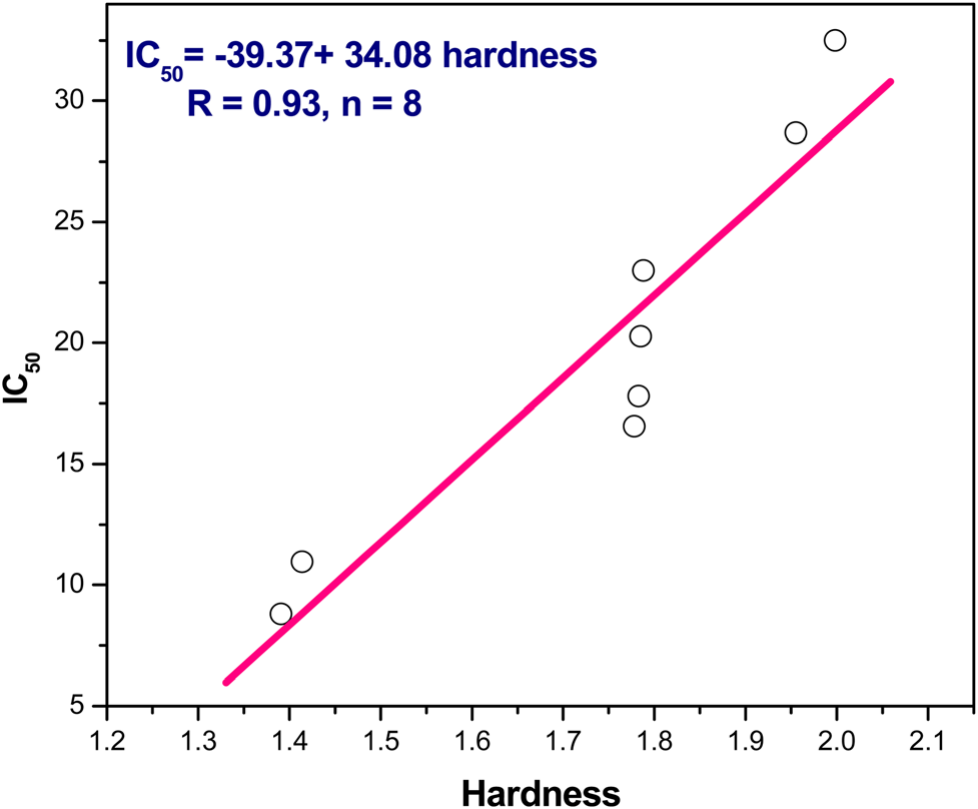
The linear correlation between hardness *versus* IC_50_.

**Fig. 9:**
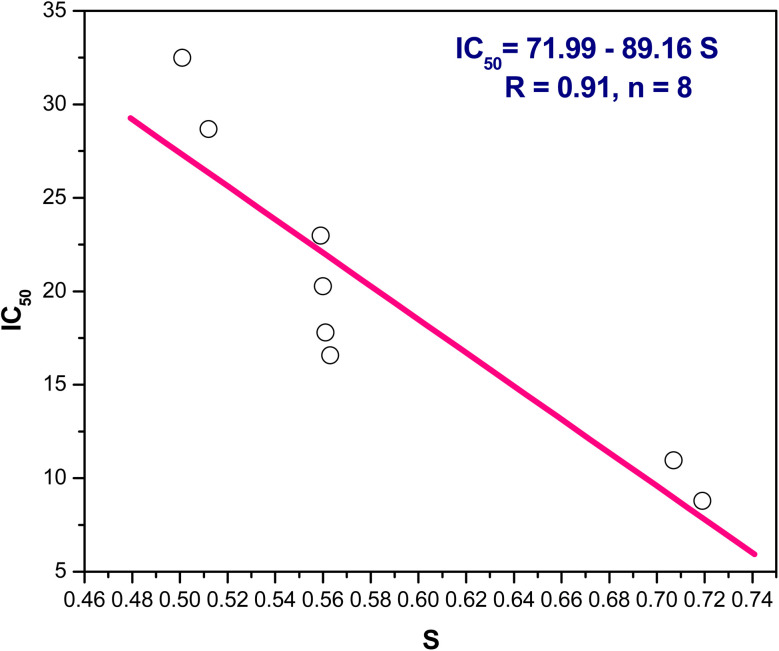
The linear correlation between softness (*S*) *versus* IC_50_.

Linear regression analysis was performed using the experimental inhibitory activity (IC_50_) as the dependent variable and DFT-based global reactivity descriptors as the independent variables for the gas phase (Table 1). From the linear regression, the following are the main interest points:

(1) The positive slope of linear correlation IC_50_ = −39.47 + 17.07 Δ*E*/eV, *r* = 0.93, *n* = 8, indicated lowering energy gap (Δ*E*/eV) of the present compounds led to decrease the half-maximal inhibitory concentration (IC_50_) and therefore enhance the anticancer activity (Fig. 7).

(2) In addition, the positive slope of IC_50_ and hardness, IC_50_ = −39.37 + 34.08 *η*/eV, *r* = 0.93, *n* = 8, indicating IC_50_ falls, as well increasing inhibitory action, with decreasing hardness (Fig. 8). Additionally, IC_50_ and softness have an inverse relationship where the softness *versus* IC_50_ showed a negative slope as follows IC_50_ = 71.99–89.16 *S*/eV, *r* = 0.91, *n* = 8 points (Fig. 9).

From the above results we can conclude that, lowering the energy gap indicates more susceptibility to accept electron from donor species. The molecule with a small energy gap which is a soft molecule was more polarized and reactive than hard ones due to its easily offering of the electrons to an acceptor. The energy gap of compounds 3 and 4 are smaller than other compounds indicating the ease of charge transfer and therefore enhanced the biological activity.^74^

#### 3.3.2. Drug similarity and *in silico* ADME anticipation

SwissADME was used to perform a computational ADME analysis on the compounds in order to determine their physicochemical and drug-like characteristics.^75,76^ The current compounds adhere to Lipinski's rule, which states that *M* log *P* should be less than 5, hydrogen bond donor (HBD) should not be greater than 5, hydrogen bond acceptor (HBA) should not be greater than 10, and MW should be less than 500 amu. Molecular weight of compounds is between 290 and 425 g mol^−1^, which is the permissible range. Compounds have an HBA between 6 and 8 and HBD between 1 and 4, both of which are within acceptable bounds. Moreover, the estimated values of the log octanol/water partition coefficient (*M* log *P*) range from −1.48 to 1.06 (Table 9). Additionally, Veber's rule outlines the following restrictions on drug likeness: (a) the total polar surface area (TPSA) is associated with drug bioavailability and should not exceed 140 Å; the values for these compounds fall between 112.66 and −139.83 Å. (b) The number of rotatable bonds that is less than 10 and the produced compounds 1–2's rotatable bond count (Table 9). Additionally, the Ghose filter covers the following ranges: MW ranges from 160 to 480, MR ranges from 40 to 130, *W* log *P* ranges from −0.4 to 5.6, and the total number of atoms ranges from 20 to 70 (Table 9). Additionally, all compounds exhibit favorable qualities for oral bioavailability by adhering to Lipinski's, Ghose's, and Veber's criteria.

**Table 9:** Estimations of ADMET and physicochemical properties of compounds 1–9

Compound no.	MW	*M* log *P*	HBA	HBD	TPSA	nRotB	*W* log *P*	MR	Lipinski violations	Ghose violations	Veber violations
1	290.23	−0.51	6	1	112.66	1	0.22	74.24	0	0	0
2	354.27	−0.49	7	3	134.93	2	1.08	93.11	0	0	0
3	402.32	−1.48	8	3	135.44	2	1.2	105.51	0	0	0
4	427.37	1.06	7	2	131.37	2	3.2	118.75	0	0	0
5	369.29	0.17	7	4	139.82	2	1.09	98.05	0	0	0
6	367.32	0.43	7	3	133.85	2	2.1	100.19	0	0	0
7	397.3	−0.04	8	4	132.89	2	0.45	104.32	0	0	0
8	413.36	−0.1	7	4	131.91	2	1.82	108.88	0	0	0
9	425.35	0.02	8	2	135.17	2	0.47	114.12	0	0	0

#### 3.3.3. Molecular docking studies

Docking simulation is a highly effective technique extensively utilized in drug discovery and molecular biology to predict interactions between small molecules and target proteins. It has been employed in numerous studies to validate the biological activities of compounds, particularly in areas such as DNA binding,^77^ anticancer,^78^ and antibacterial^59^ and insecticidal^79^ activities. These instances underscore the versatility and efficacy of docking simulations in revealing the biological functions of various compounds, thereby playing a crucial role in the progress of drug discovery and development.^59,77–79^

Initially, docking methodology had been validated by re-docking and superimposition of the native ligand, *N*-[4-(acridin-9-ylamino)-3-methoxyphenyl] methanesulfonamide, which was co-crystallized with the 4G0U active site. The binding pattern of the co-crystallized ligand was successfully reproduced during the re-docking validation stage, yielding a binding energy of −9.74 kcal mol^−1^. The superimposition between the re-docked ligand and the native co-crystallized ligand showed a close alignment with the re-docked and native co-crystallized ligand, further confirming the accuracy of the docking methodology, Fig. S30.†

**Fig. 10:**
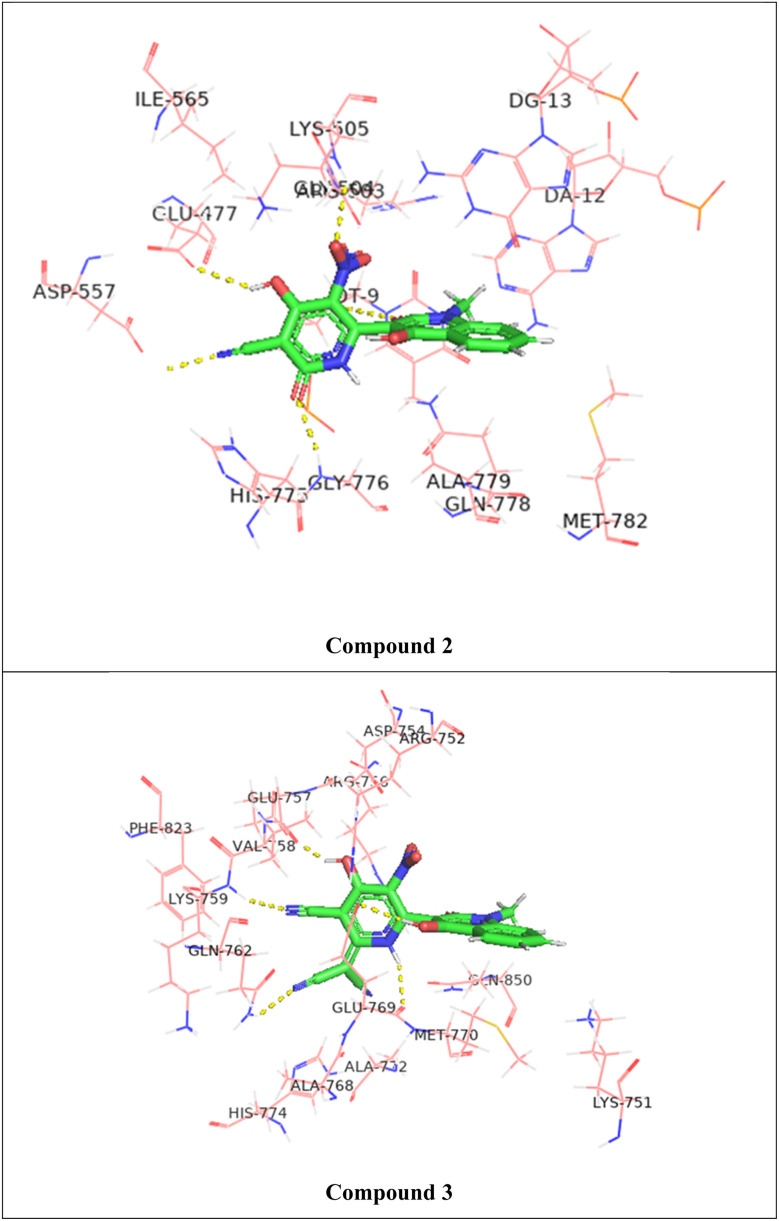
3D representation of the hydrogen bonding between the studied compounds (2 and 3) and the amino acids residues of the target protein (PDB ID: 4G0U).

The molecular docking of the studied compounds against the crystal structure of the Human topoisomerase II beta in complex with DNA and amacrine (PDB ID: 4G0U) was performed and the best binding mode with docked compounds are shown in Fig. 10 and S31† while the interactions with amino acids residues were shown in Table 10. The binding energy of the standard etoposide is −8.54 kcal mol^−1^, while that of the current compounds are ranged between −7.41 and −8.44 kcal mol^−1^. The percent (%) of the compounds' binding energy to that of the standard etoposide (−8.54 kcal mol^−1^) had been calculated and reported in Table 10.

**Table 10:** Hydrogen bonds between the studied products 2–9 and the amino acids residues of the target protein (PDB ID: 4G0U)

Compound	Ligand	Receptor	Distance	Binding energy (kcal mol^−1^)	% (standard, etoposide)
2	O 25	LYS 505	3.36	−8.23	96.37
	H 35	GLU 477	3.14		
	O 22	GLY 776	3.04		
	O 22	DT 9	3.11		
	O 13	DT 9	3.15		
3	H 39	GLU 757	3.03	−8.44	98.83
	N 24	LYS 759	2.76		
	N 29	GLN 762	3.14		
	O 11	GLU 769	3.56		
	H 40	GLU 769	3.47		
4	O 13	ARG 729	3.1	−8.13	95.20
	O 31	HIS 775	3.46		
	O 32	HIS 775	3.58		
	O 14	GLU 477	3.48		
	N 22	LYS 505	3.24		
5	O 11	GLN 850	2.91	−8.09	94.73
	H 38	ALA 768	2.89		
	O 27	LYS 759	3.25		
	H 37	TYR 773	3.65		
6	O 11	GLN 762	3.07	−7.41	86.77
	O 26	LYS 759	3.06		
	O 36	GLU 769	3.22		
	H 37	GLU 769	3.18		
7	O 13	GLN 762	2.76	−7.75	90.75
	H 38	GLU 769	2.91		
	H 38	GLN 850	3.27		
	O 29	GLN 850	3.11		
	O 29	GLU 769	3.24		
	O 28	GLU 757	3.38		
	H 40	GLU 757	3.18		
8	O 11	DC 14	3.35	−8.02	93.91
	O 23	GLN 778	3.17		
	O 20	LYS 505	4.47		
	O 14	DC 13	3.79		
9	O 28	LYS 505	3.51	−7.74	90.63
	N 16	DC 13	3.21		
	O 13	GLN 778	3.52		
	O 14	GLN 778	3.44		

Compound 2 exhibits a binding affinity of −8.23 kcal mol^−1^, forming hydrogen bonds with several residues. The oxygen atom at position 25 interacts with LYS 505 at a distance of 3.36 Å. Additionally, compound 2 forms hydrogen bonds with GLU 477 (3.14 Å), GLY 776 (3.04 Å), and two bonds with the DT9 residue (3.11 Å and 3.15 Å).

Compound 3 shows a slightly higher binding affinity of −8.44 kcal mol^−1^. This compound forms hydrogen bonds with GLU 757 (3.03 Å), LYS 759 (2.76 Å), GLN 762 (3.14 Å), and two bonds with GLU 769 (3.56 Å and 3.47 Å).

Compound 4 demonstrates a binding affinity of −8.13 kcal mol^−1^, with hydrogen bonds formed between O13 and ARG 729 (3.1 Å), O31 and HIS 775 (3.46 Å), O32 and HIS 775 (3.58 Å), O14 and GLU 477 (3.48 Å), and N22 and LYS 505 (3.24 Å).

Compound 5 has a binding affinity of −8.09 kcal mol^−1^ and forms hydrogen bonds with GLN 850 (2.91 Å), ALA 768 (2.89 Å), LYS 759 (3.25 Å), and TYR 773 (3.65 Å).

Compound 6 exhibits a lower binding affinity of −7.41 kcal mol^−1^, with hydrogen bonds formed with GLN 762 (3.07 Å), LYS 759 (3.06 Å), GLU 769 (3.22 Å), and another bond with GLU 769 (3.18 Å).

Compound 7 shows a binding affinity of −7.75 kcal mol^−1^, with interactions with GLN 762 (2.76 Å), GLU 769 (2.91 Å), GLN 850 (3.27 Å), GLU 769 (3.24 Å), GLU 757 (3.38 Å), and another bond with GLU 757 (3.18 Å).

Compound 8 has a binding affinity of −8.02 kcal mol^−1^, forming hydrogen bonds with DC 14 (3.35 Å), GLN 778 (3.17 Å), LYS 505 (4.47 Å), and DC 13 (3.79 Å).

Compound 9 exhibits a binding affinity of −7.74 kcal mol^−1^, with hydrogen bonds formed with LYS 505 (3.51 Å), DC 13 (3.21 Å), and two bonds with GLN 778 (3.52 Å and 3.44 Å).

In general, compounds 2–4 show the highest binding affinities, indicating their stronger interactions with the target protein. The order of binding affinities, from highest to lowest, is as follows: compound 3 > compound 2 > compound 4 > compound 5 > compound 8 > compound 7 > compound 9 > compound 6.

Compound 3, with the highest binding affinity, demonstrates the most favorable interactions, particularly with residues such as LYS 759 and GLU 769. Conversely, compound 6, with the lowest binding affinity, shows weaker interactions, suggesting it may be less effective in binding to the target protein.

## 4. Conclusion

• A diversity of 4-hydroxy-1-methylquinolin-2(1*H*)-one tethered pyridines, pyrido[1,2-*a*]benzimidazole, pyrazolo[3,4-*b*]pyridines and pyrido[2,3-*d*]pyrimidines were efficiently synthesized from recyclization reactions of the starting substrate 1 with some carbon nucleophilic reagents.

• In quantum chemical calculations, the B3LYP/6-311++G (d,p) basis set was utilized to optimize the prepared compounds. From FMO, compound 3 displayed lower energy gap and lower hardness which coupled with higher softness; suggesting higher reactivity and reduced kinetic stability compared to other compounds.

• A good agreement was found for both computational and experimental infrared (IR) and nuclear magnetic resonance (NMR) spectra, where the correlation coefficient (*R*^2^) is found in the range 0.98–0.99 for ^1^H NMR spectra and 0.99 for IR spectra.

• According to *in silico* ADME investigations, these compounds showed promise drug-likeness without violating Lipinski's, Ghose's, or Veber's criteria.

• The studied molecules were evaluated for hepatocellular carcinoma (HepG-2) cell lines revealing that, compound 3 demonstrated the most anticancer potential (IC_50_ = 8.79 μM L^−1^). Moreover, the binding energy computations have confirmed the experimental data in anticancer evaluation.

• MEP analysis identified the most favorable sites for nucleophilic and electrophilic attacks. The prepared compounds have a first-order hyperpolarizability greater that urea by about 3–18 times. Thus, these compounds may have future applications in developing novel NLO materials.

## Data availability

Data are available upon request from the authors.

## Conflicts of interest

The authors declare that they have no known competing financial interests or personal relationships that could have appeared to influence the work reported in this paper.

## Supplementary Material

RA-015-D5RA00325C-s001
