# Supplementary material for: Synthetic approaches for novel 3-heteroaryl-4-hydroxy-1-methylquinoline-2(1H)one: spectroscopic characterization, molecular docking and DFT investigations
Source: RSC Adv. 2025 Feb 28;15(9):6718–36. doi: 10.1039/d5ra00325c (PMC11869377; doi:10.1039/d5ra00325c)
Supplement: RA-015-D5RA00325C-s001 [file RA-015-D5RA00325C-s001.pdf]

Supplementary Information (SI) for RSC Advances.
This journal is © The Royal Society of Chemistry 2025

Compound	1	5	6
Optimized Structures	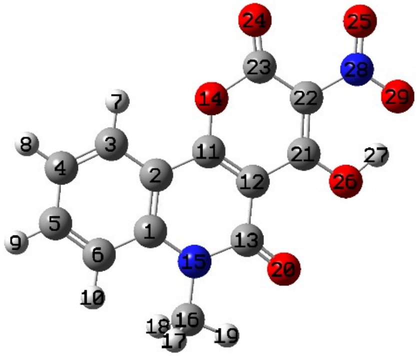	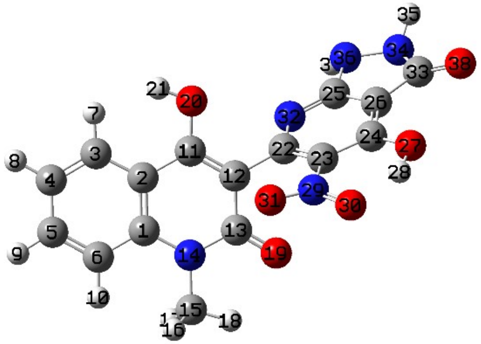	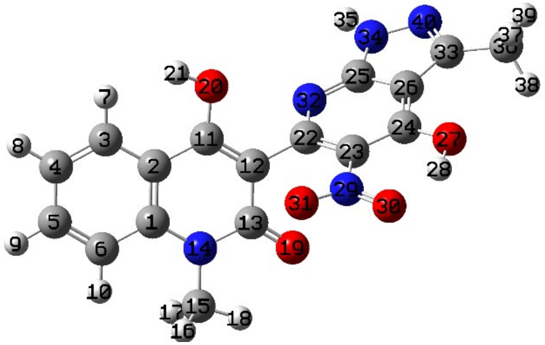
LUMO	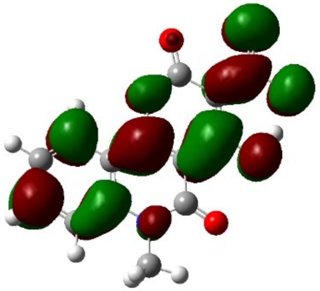 E_{LUMO} = -3.42	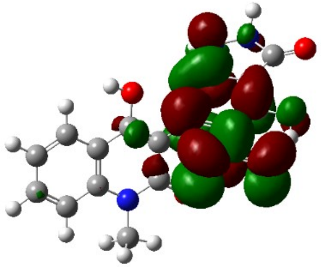 E_{LUMO} = -3.01	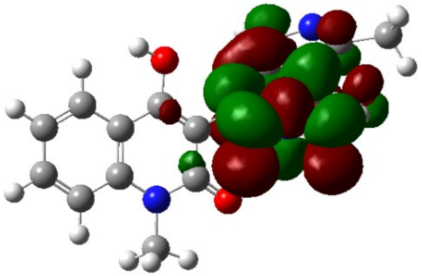 E_{LUMO}= -2.82
HOMO	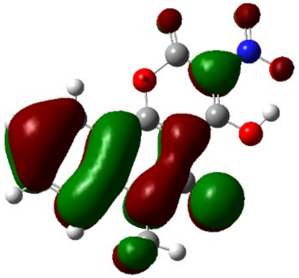 E_{HOMO}= -7.04	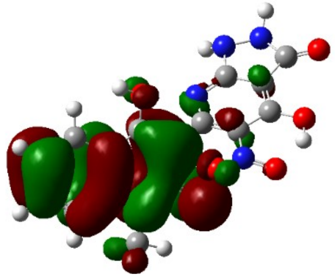 E_{HOMO}= -6.56	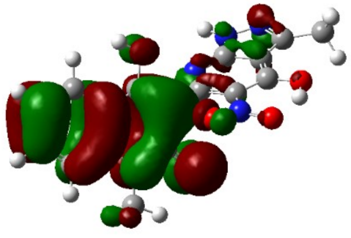 E_{HOMO} = -6.39

Fig. S1. Molecular modeling and the electron density of HOMO and LUMO of compounds **1**, **5** and **6**

Compound	7	8	9
**Optimized Structures**	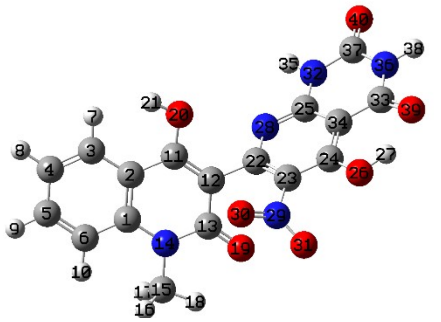	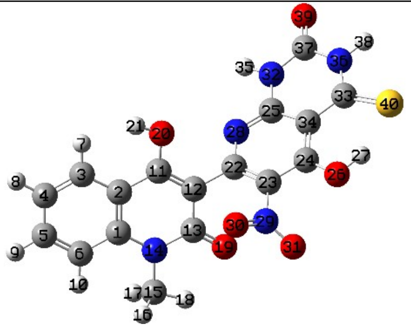	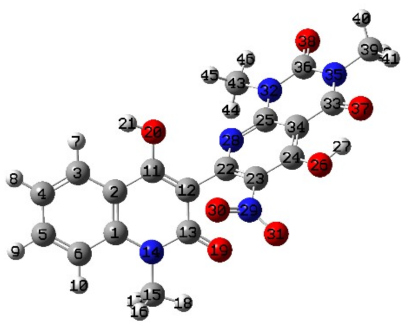
**LUMO**	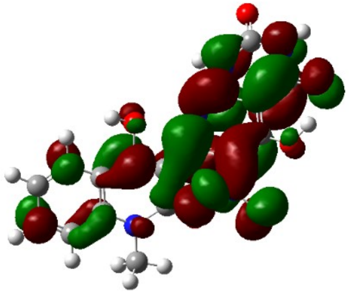 E_{LUMO} = -2.72	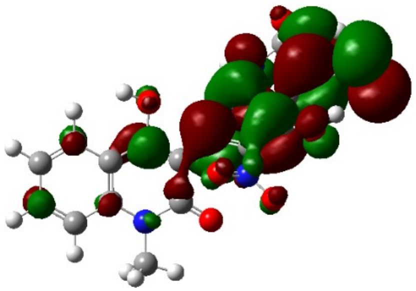 E_{LUMO} = -3.07	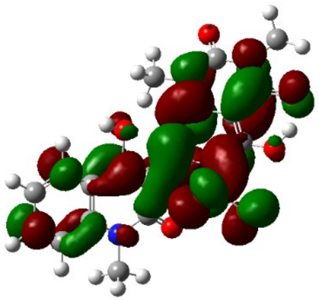 E_{LUMO}= -2.55
**HOMO**	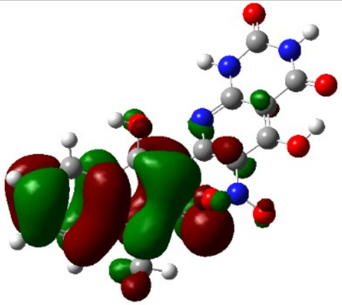 E_{HOMO}= -6.63	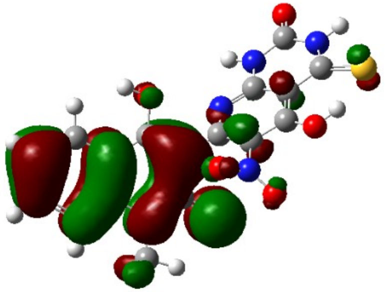 E_{HOMO}= -6.65	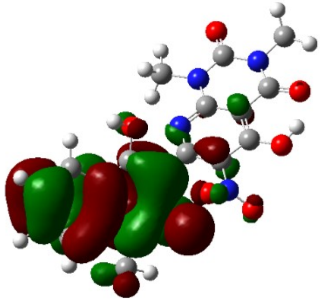 E_{HOMO} = -6.55

Fig. S2. Molecular modeling and the electron density of HOMO and LUMO of compounds **7-9**

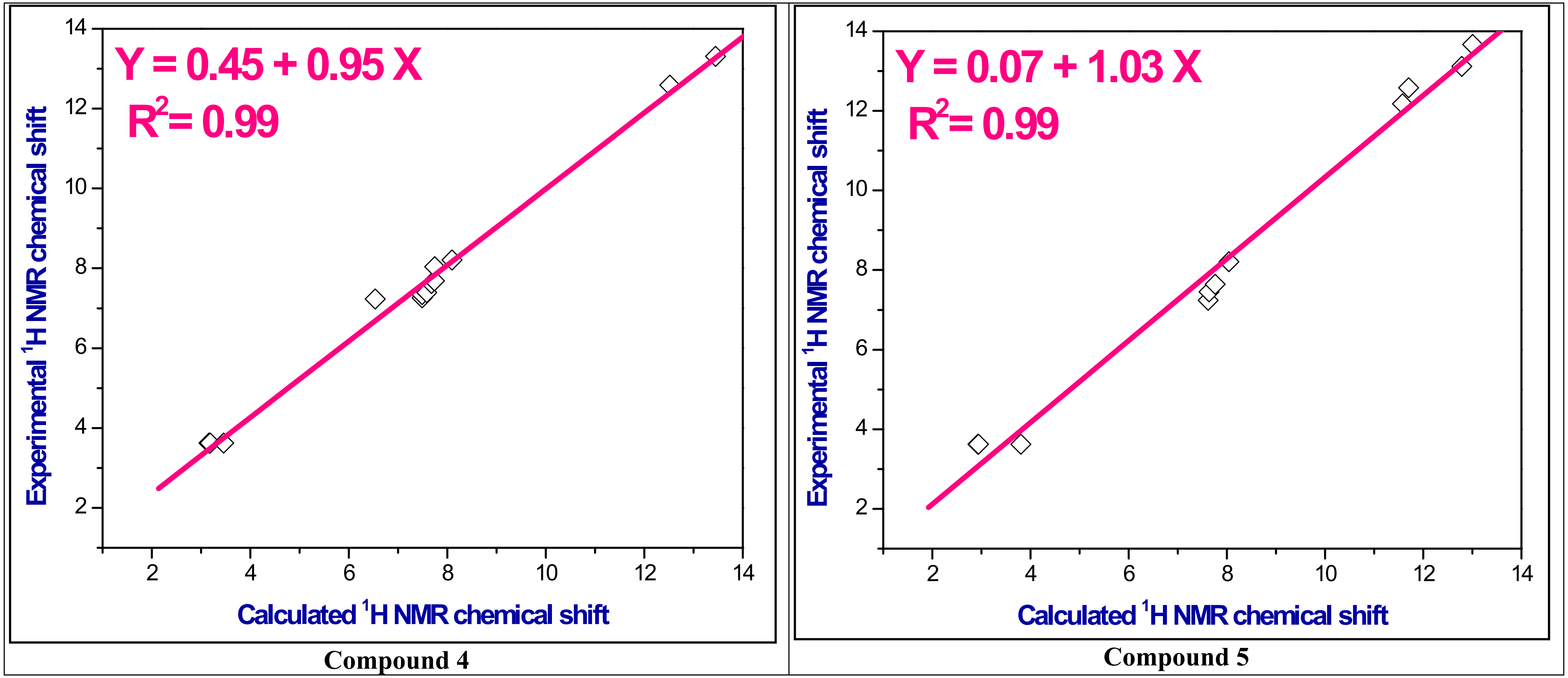

Fig. S3. The correlation relationships of the experimental *versus* calculated ^{1}H NMR chemical shifts of compounds **4** and **5**.

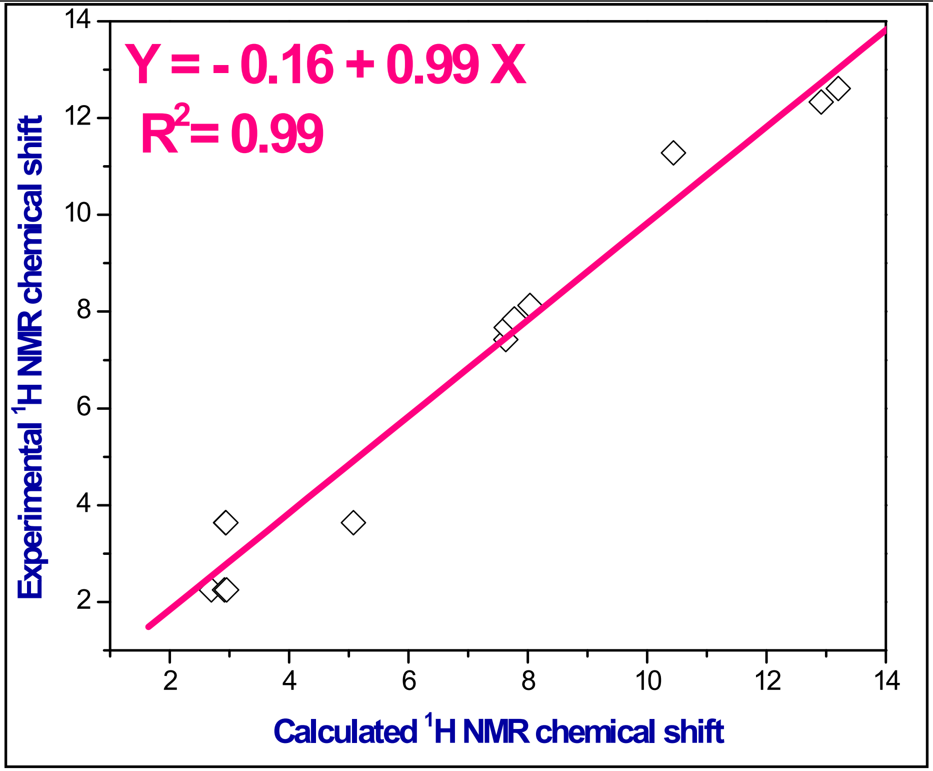

Compound 6

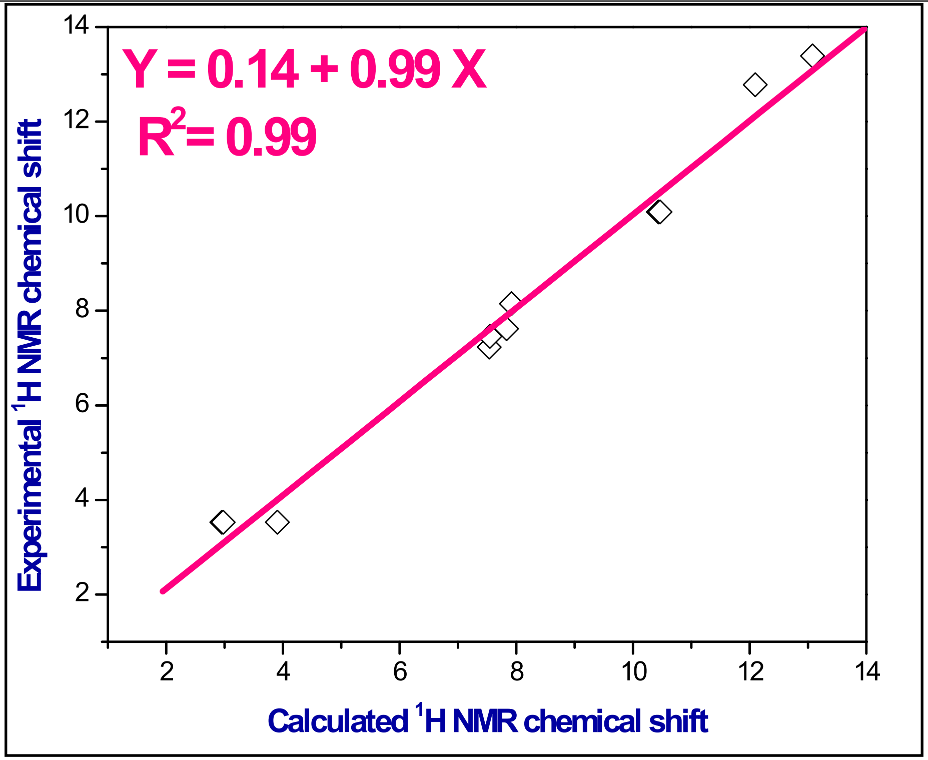

Compound 7

Fig. S4. The correlation relationships of the experimental *versus* calculated ^{1}H NMR chemical shifts of compounds **6** and **7**.

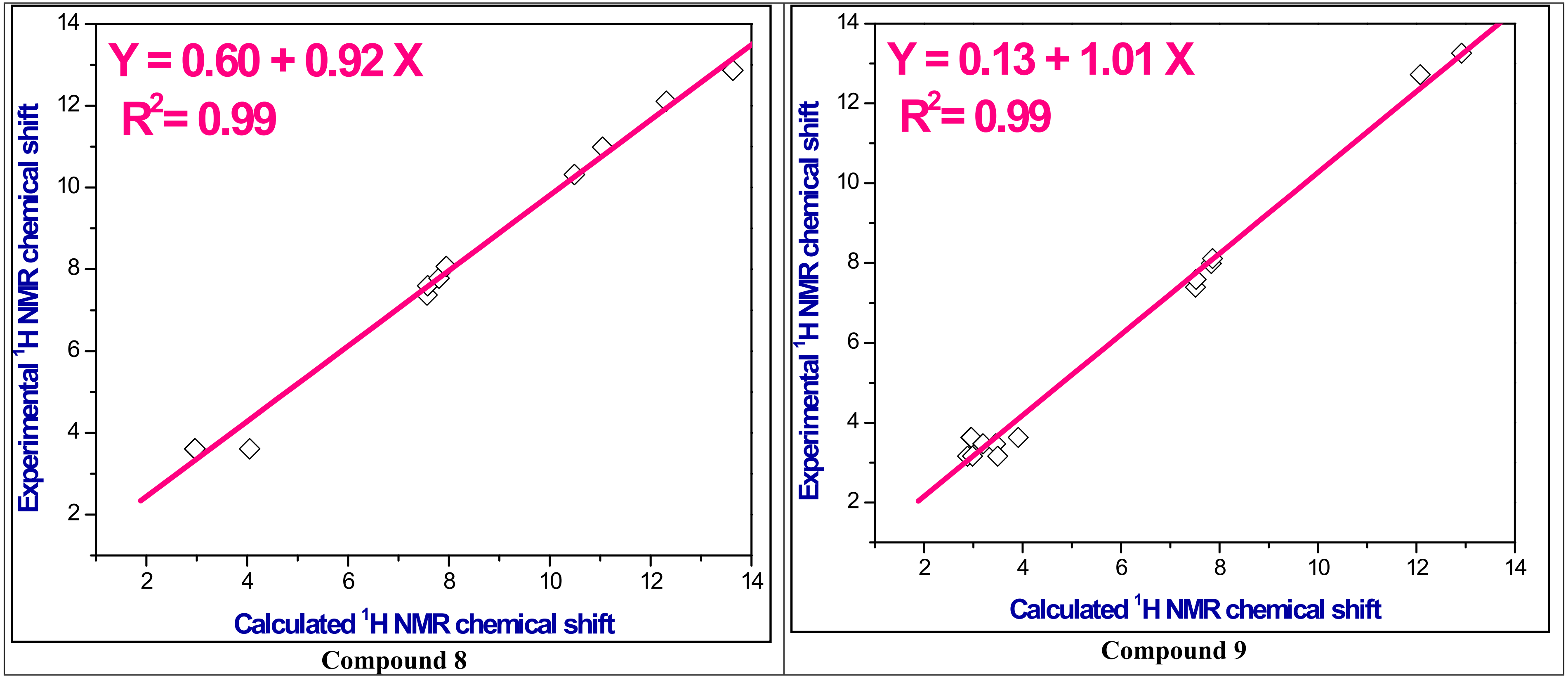

Fig. S5. The correlation relationships of the experimental *versus* calculated ^{1}H NMR chemical shifts of compounds **8** and **9**.

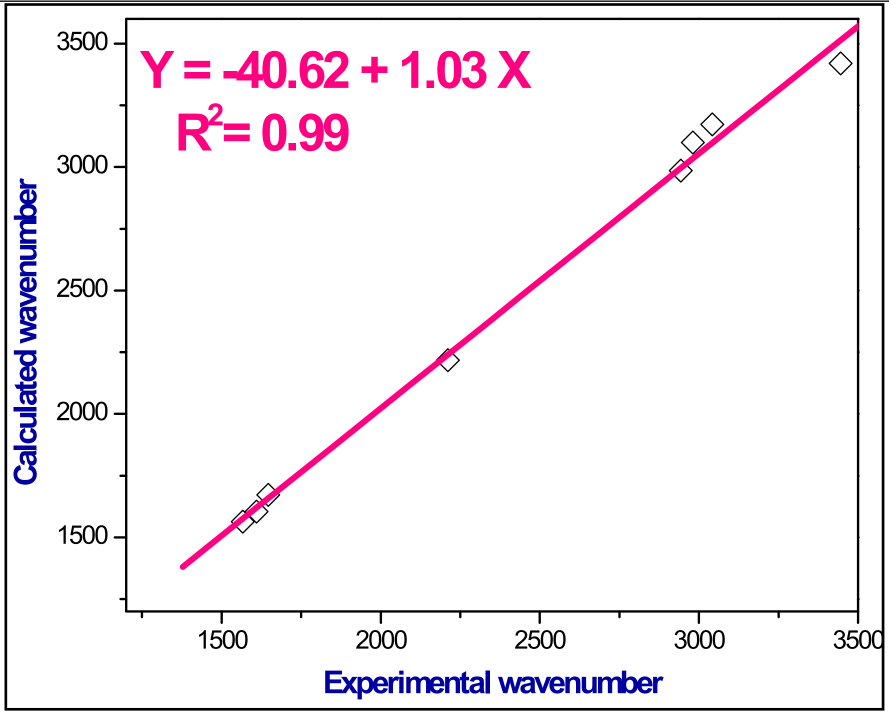

Compound 4

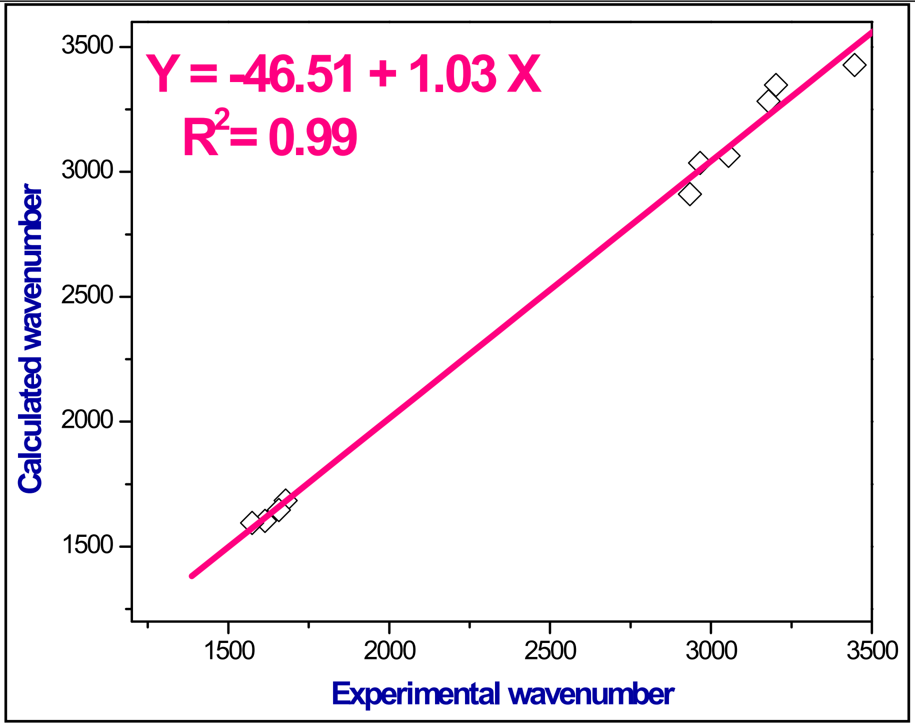

Compound 5

Fig. S6. The correlation relationships of the experimental *versus* calculated IR wavenumbers of compounds **4** and **5**.

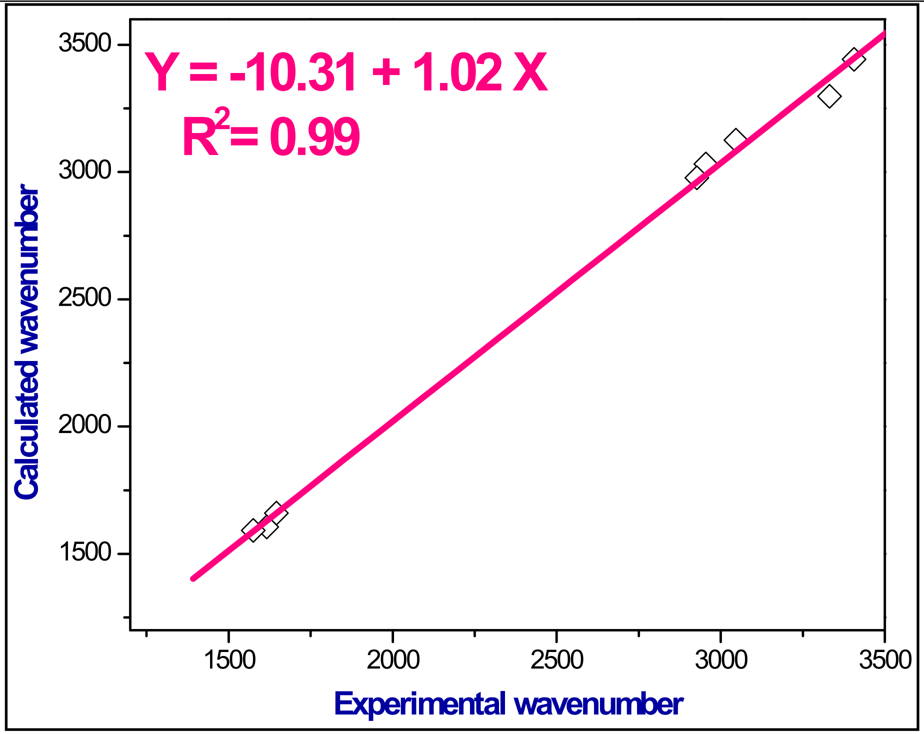

Compound 6

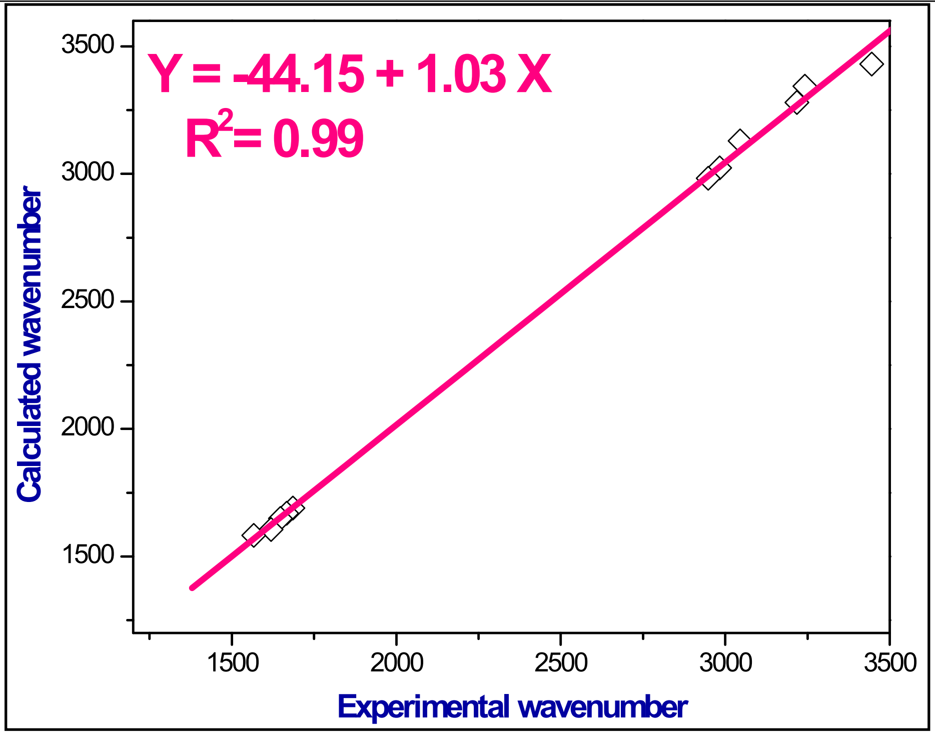

Compound 7

Fig. S7. The correlation relationships of the experimental *versus* calculated IR wavenumbers of compounds **6** and **7**.

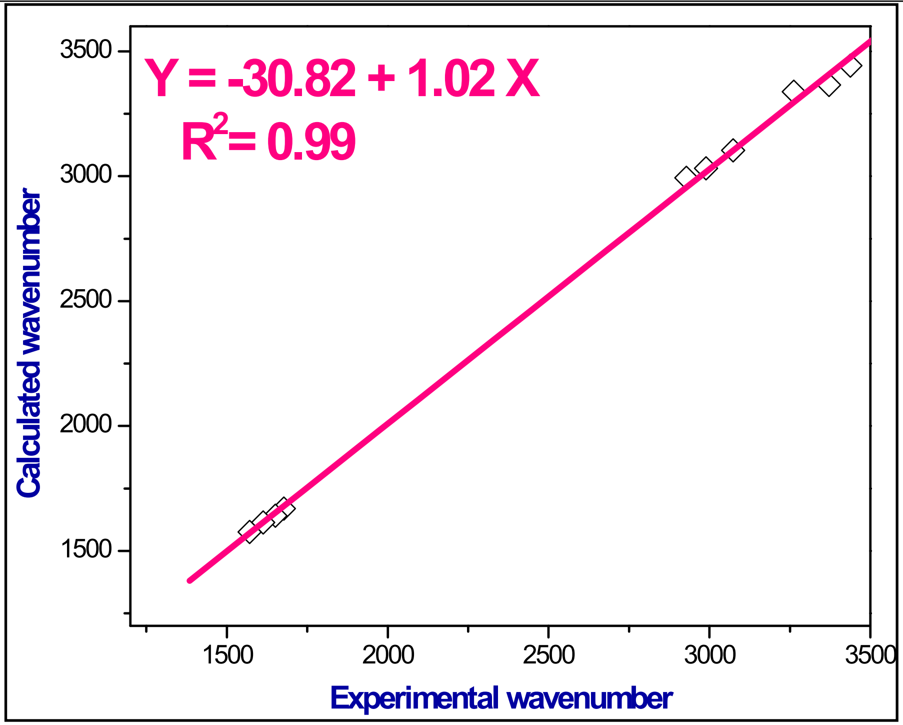

Compound 8

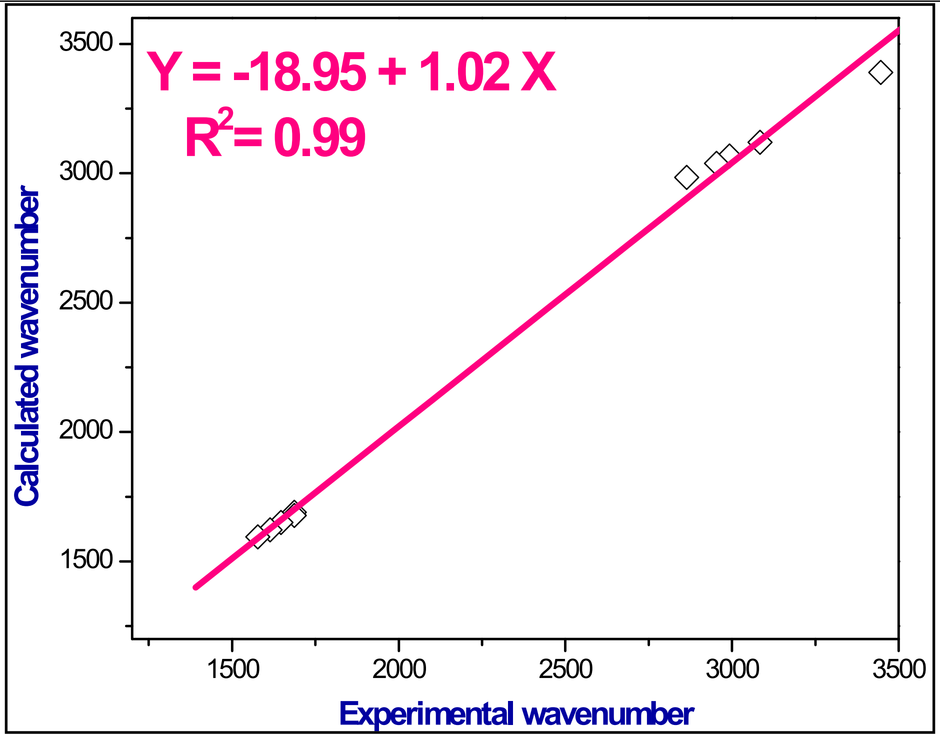

Compound 9

Fig. S8. The correlation relationships of the experimental *versus* calculated IR wavenumbers of compounds **8** and **9**.

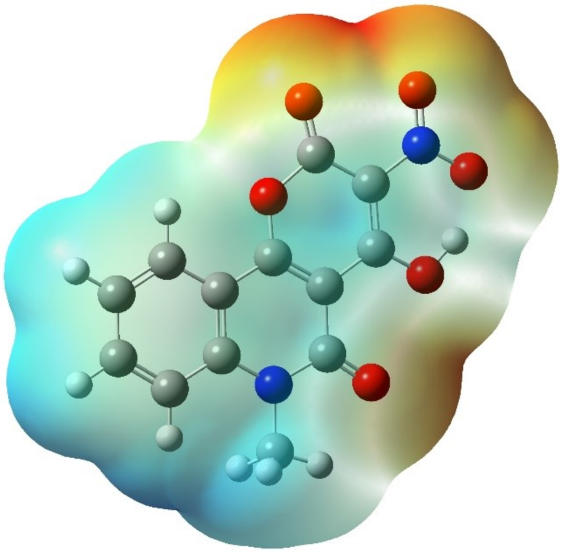

Compound **1**

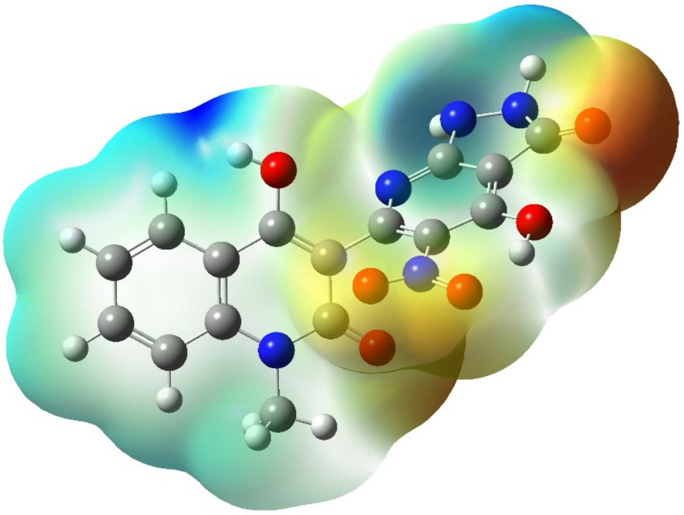

Compound **5**

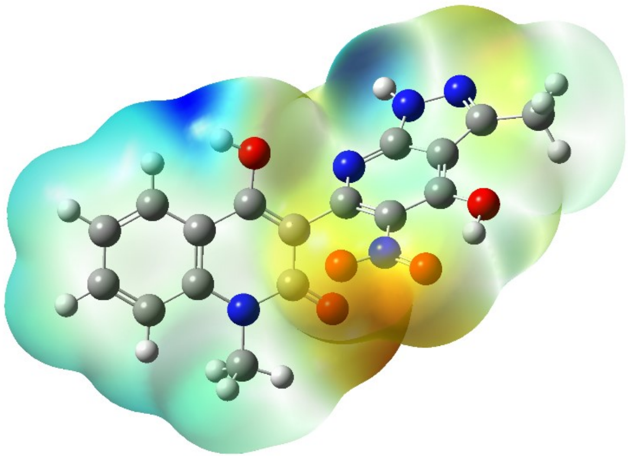

Compound **6**

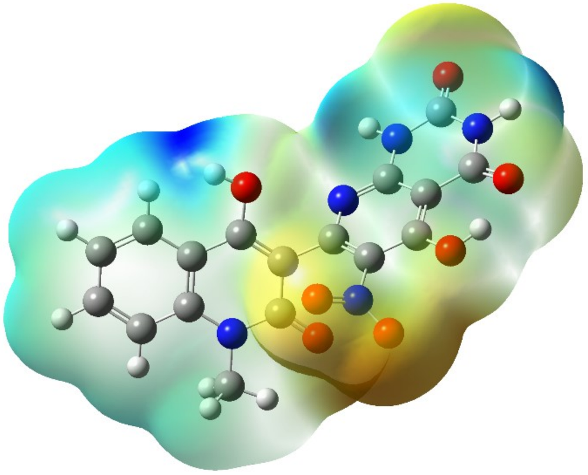

Compound **7**

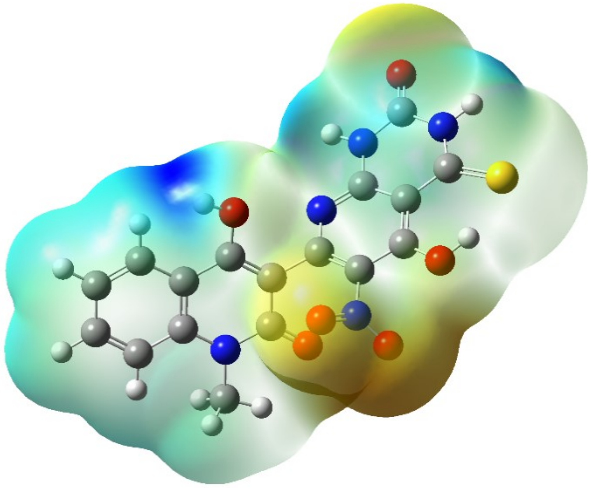

Compound **8**

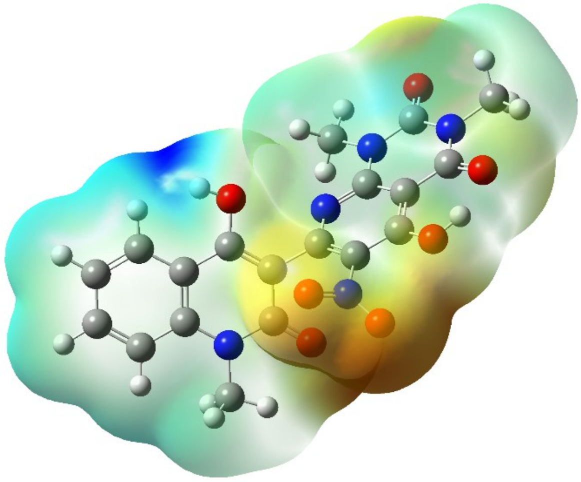

Compound **9**

Fig. S9. Molecular electrostatic potential of compounds **1**, **5-9**

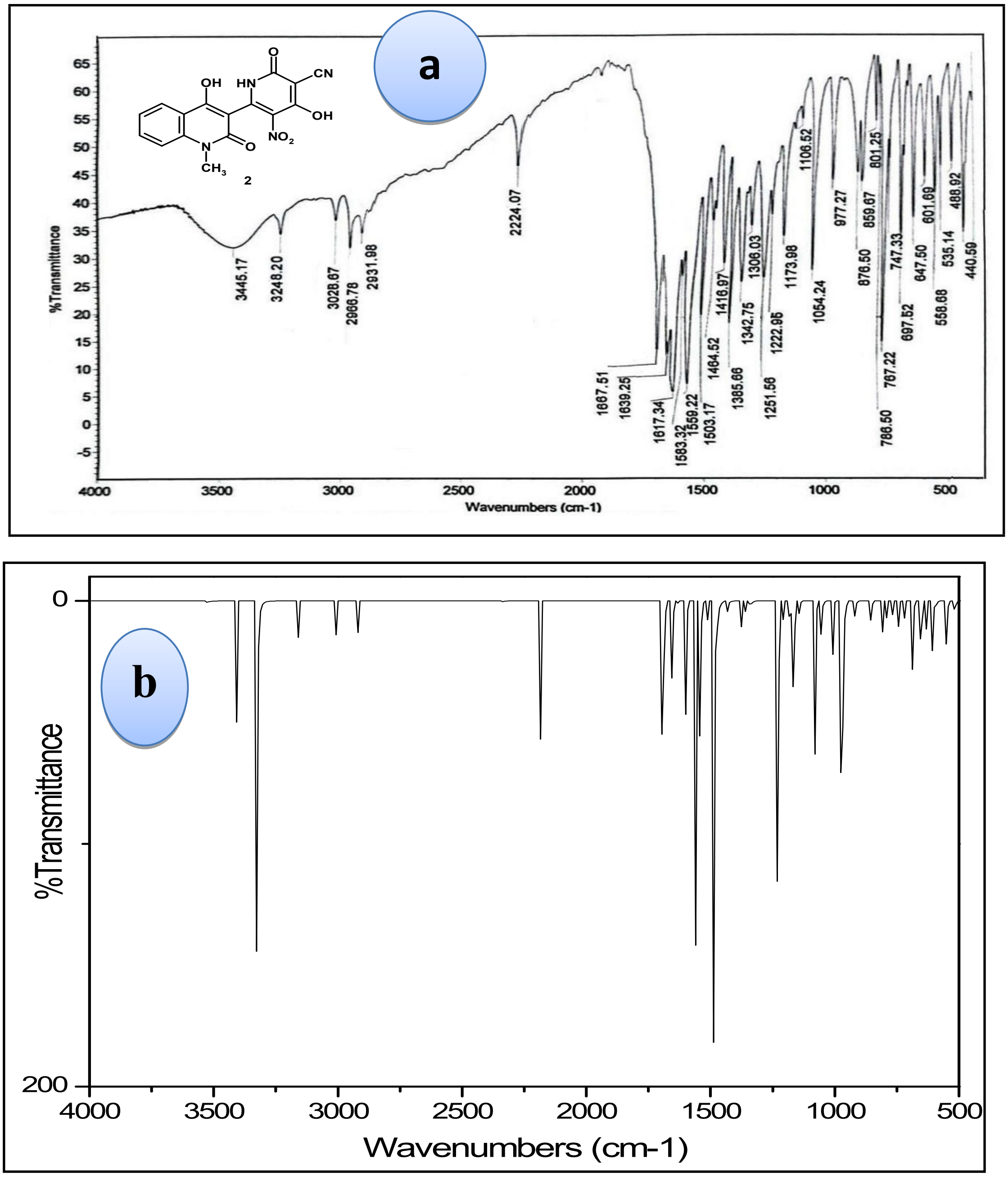

Fig. S10. (a) Experimental and (b) Calculated IR spectra of compound **2** at B3LYP/6-311++G(d,p).

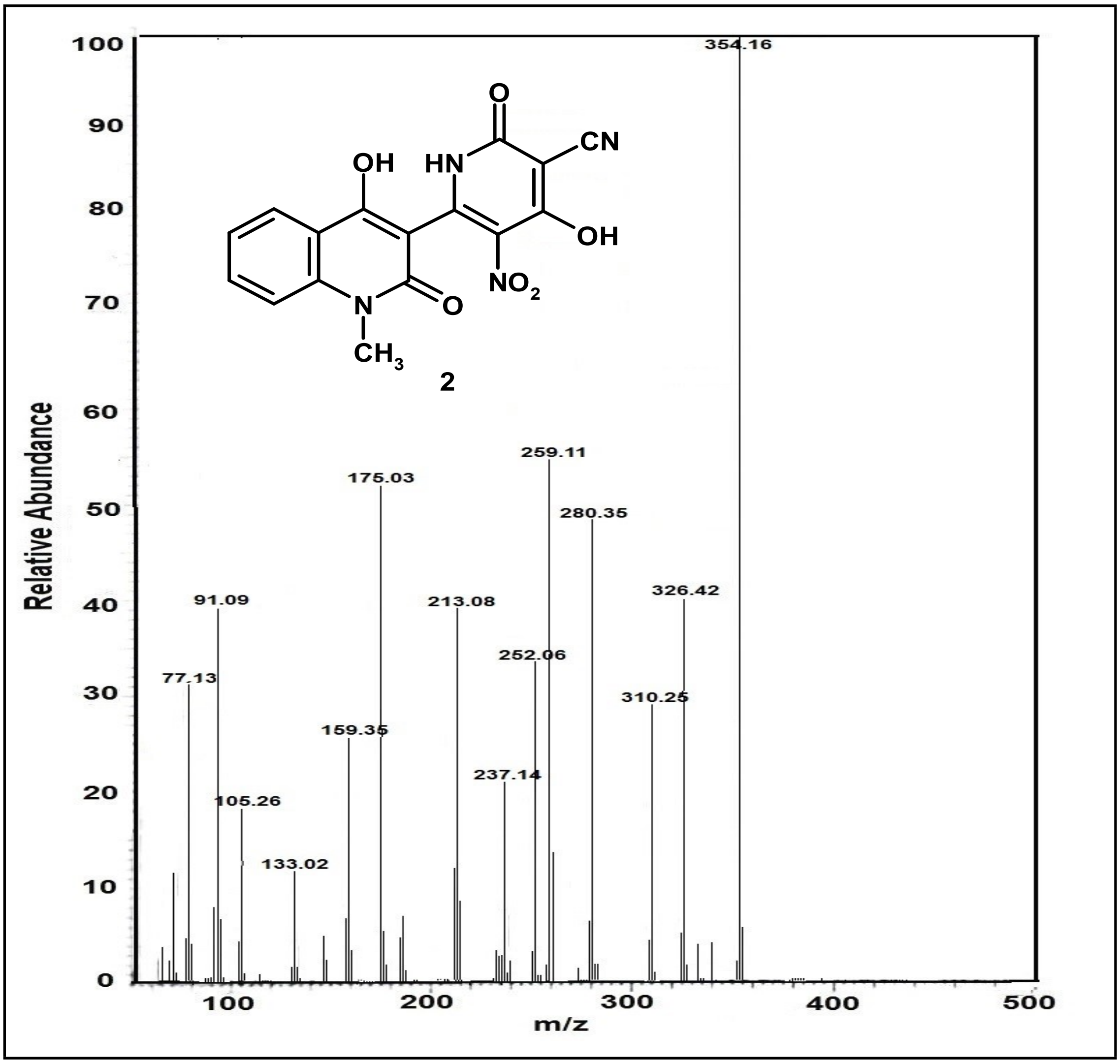

Fig. S11. Mass spectrum of compound **2**

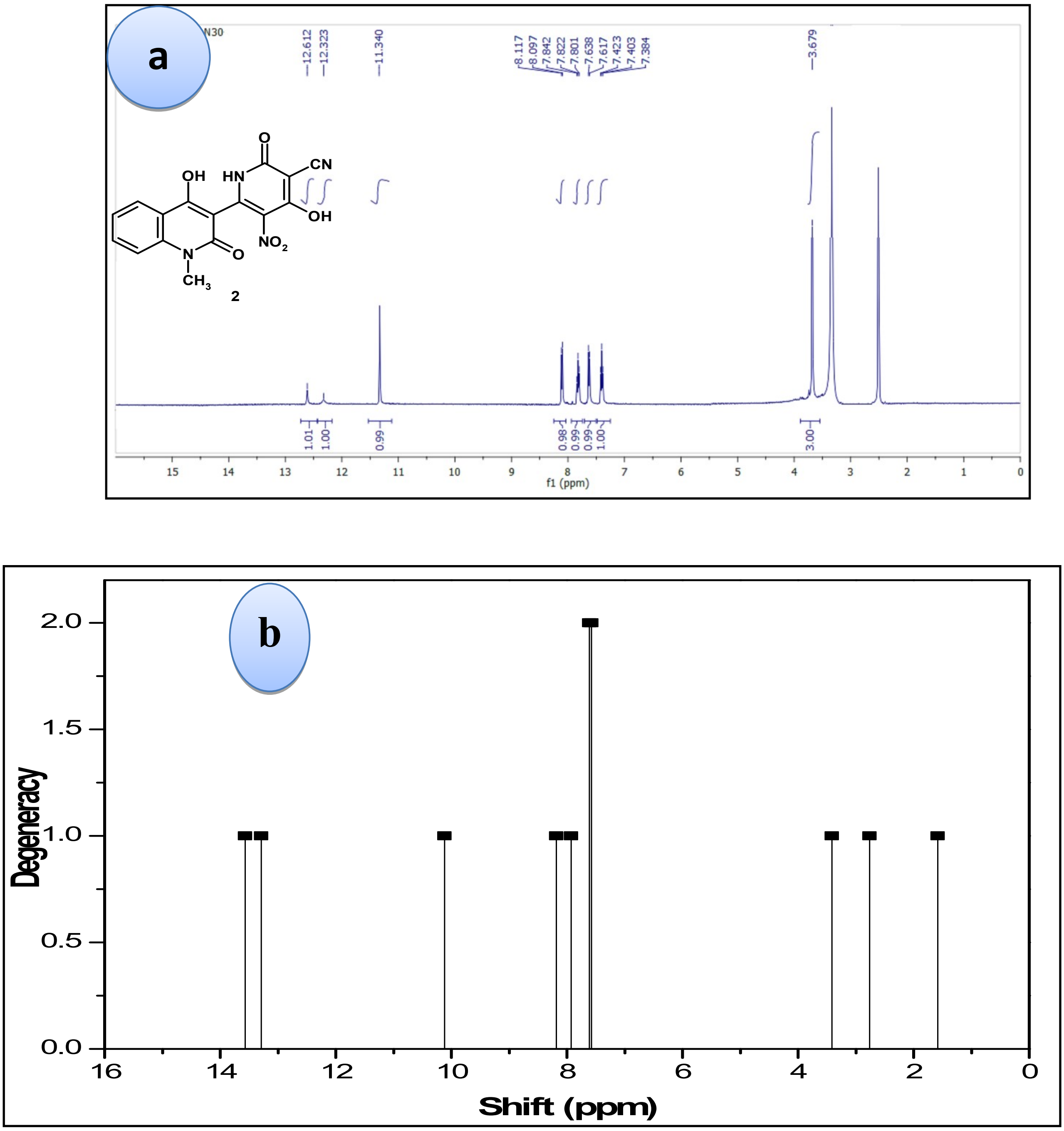

Fig. S12. (a) Experimental and (b) Calculated ^{1}H NMR spectra of compound **2** at B3LYP/6-311++G(d,p).

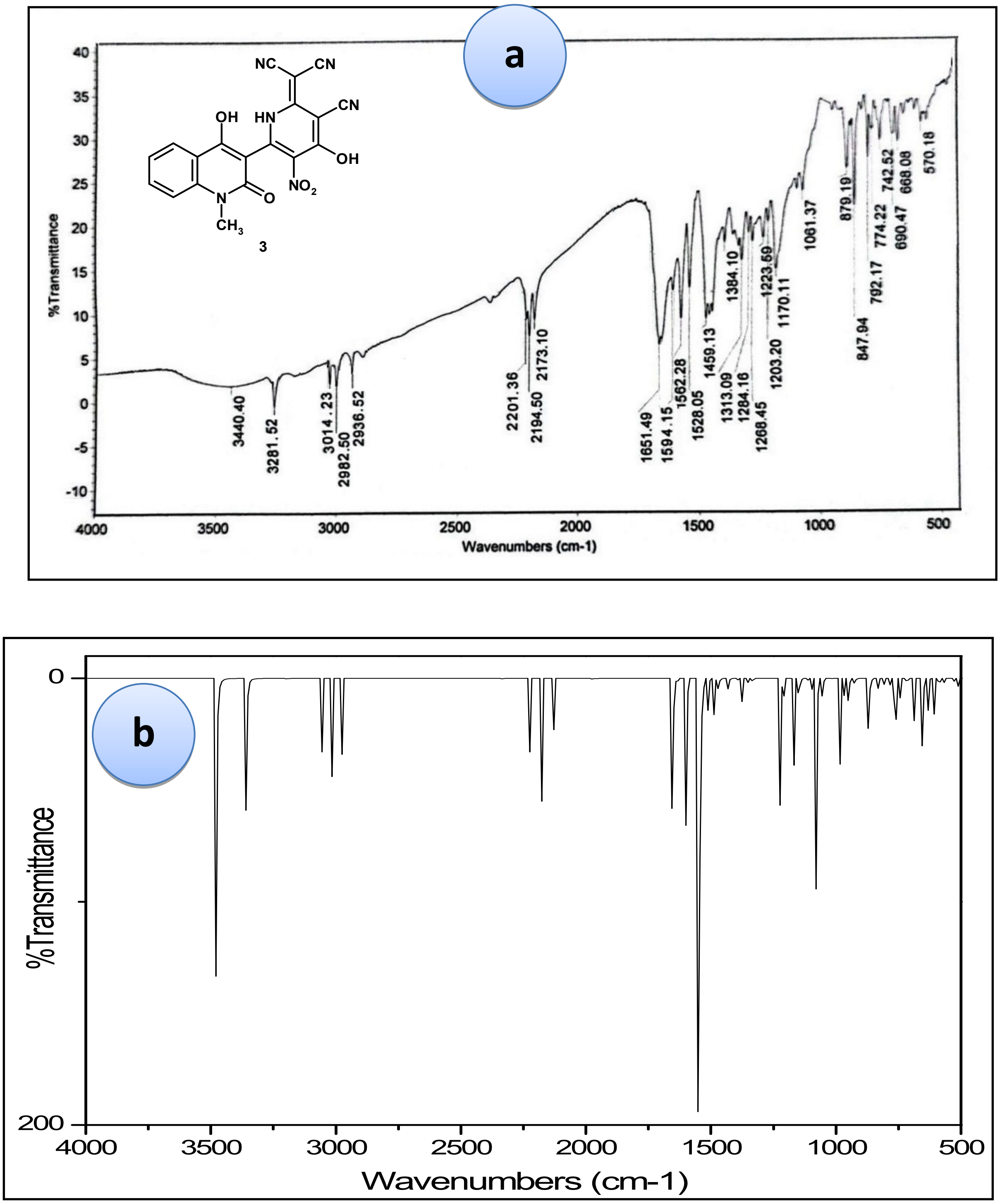

Fig. S13. (a) Experimental and (b) Calculated IR spectra of compound **3** at B3LYP/6-311++G(d,p).

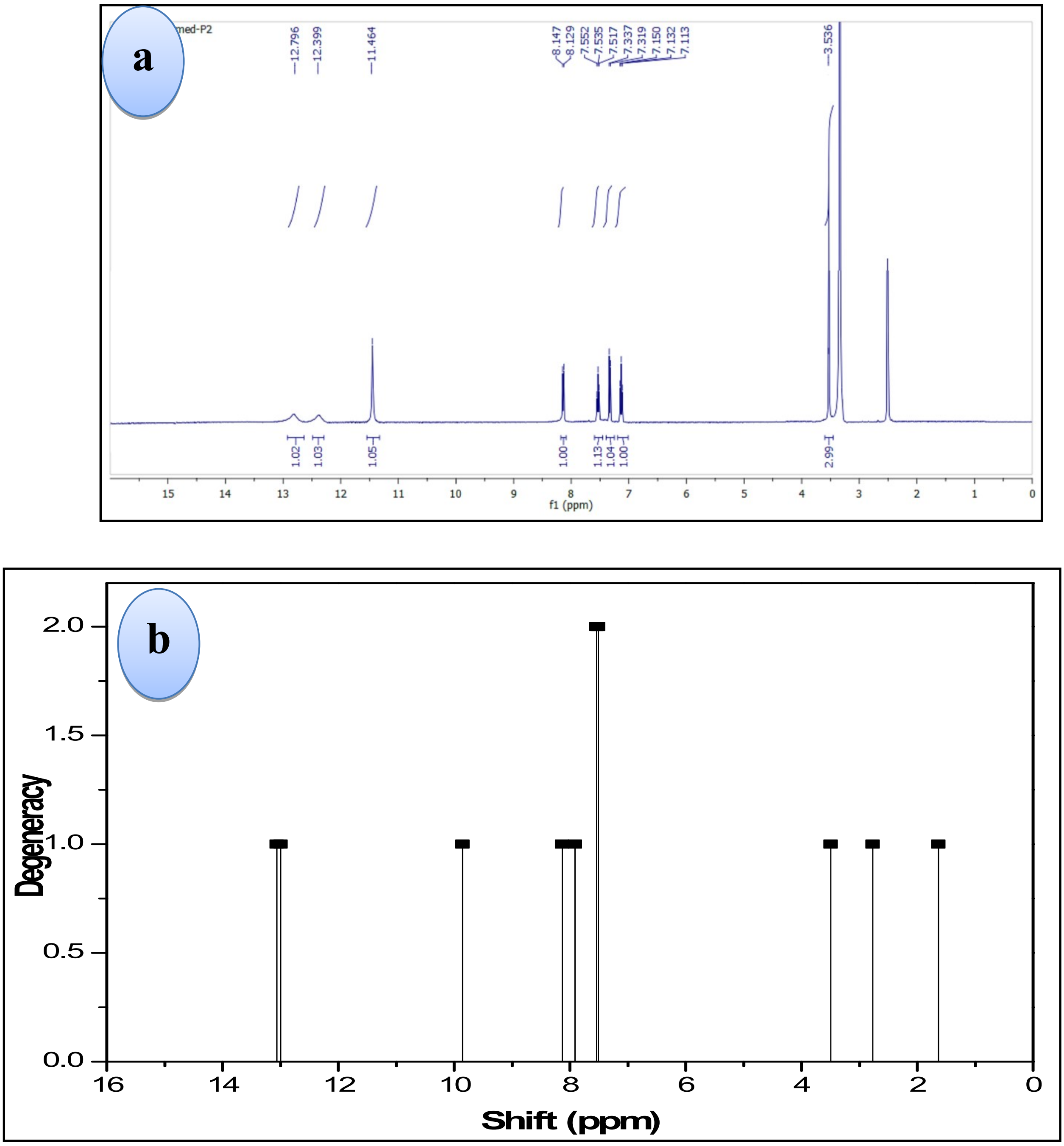

Fig. S14. (a) Experimental and (b) Calculated ^{1}H NMR spectra of compound **3** at B3LYP/6-311++G(d,p).

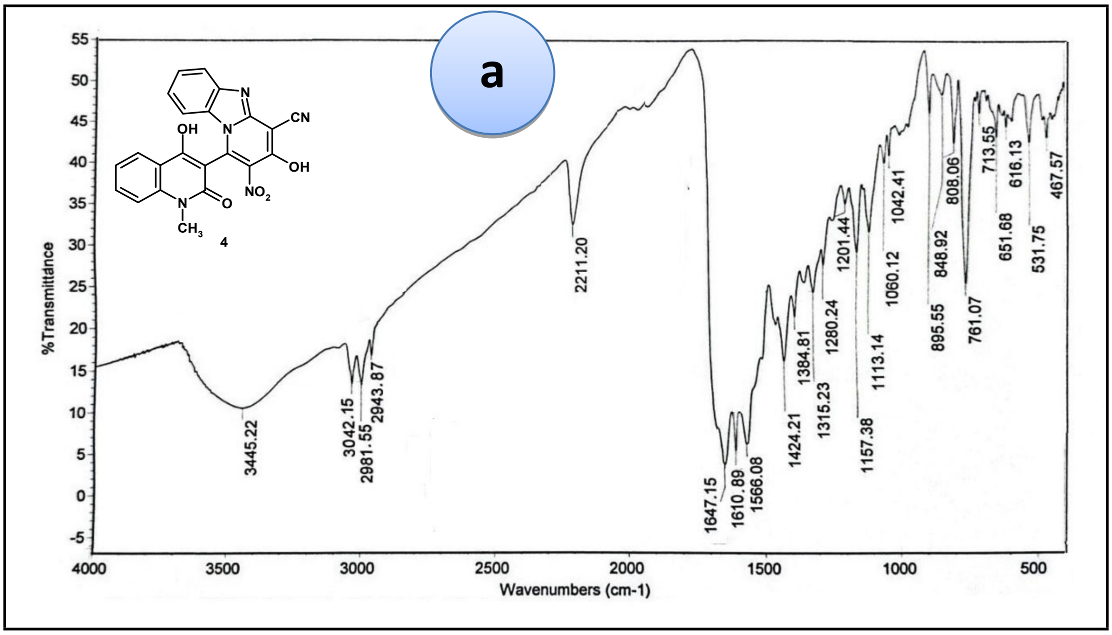

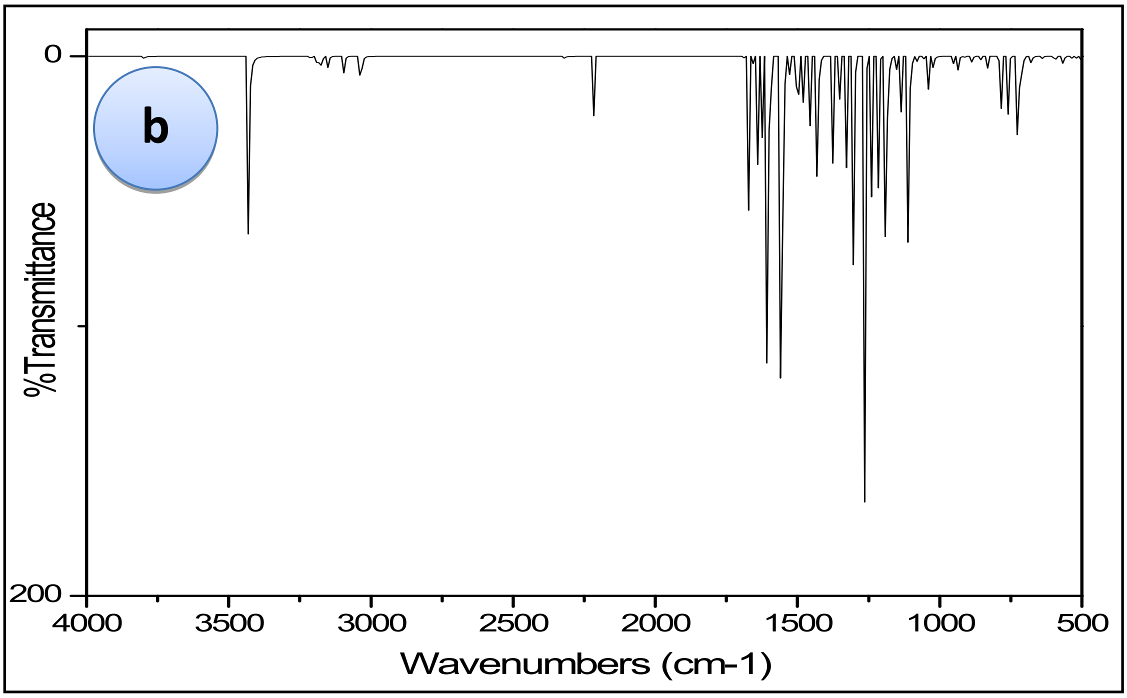

Fig. S15. (a) Experimental and (b) Calculated IR spectra of compound **4** at B3LYP/6-311++G(d,p).

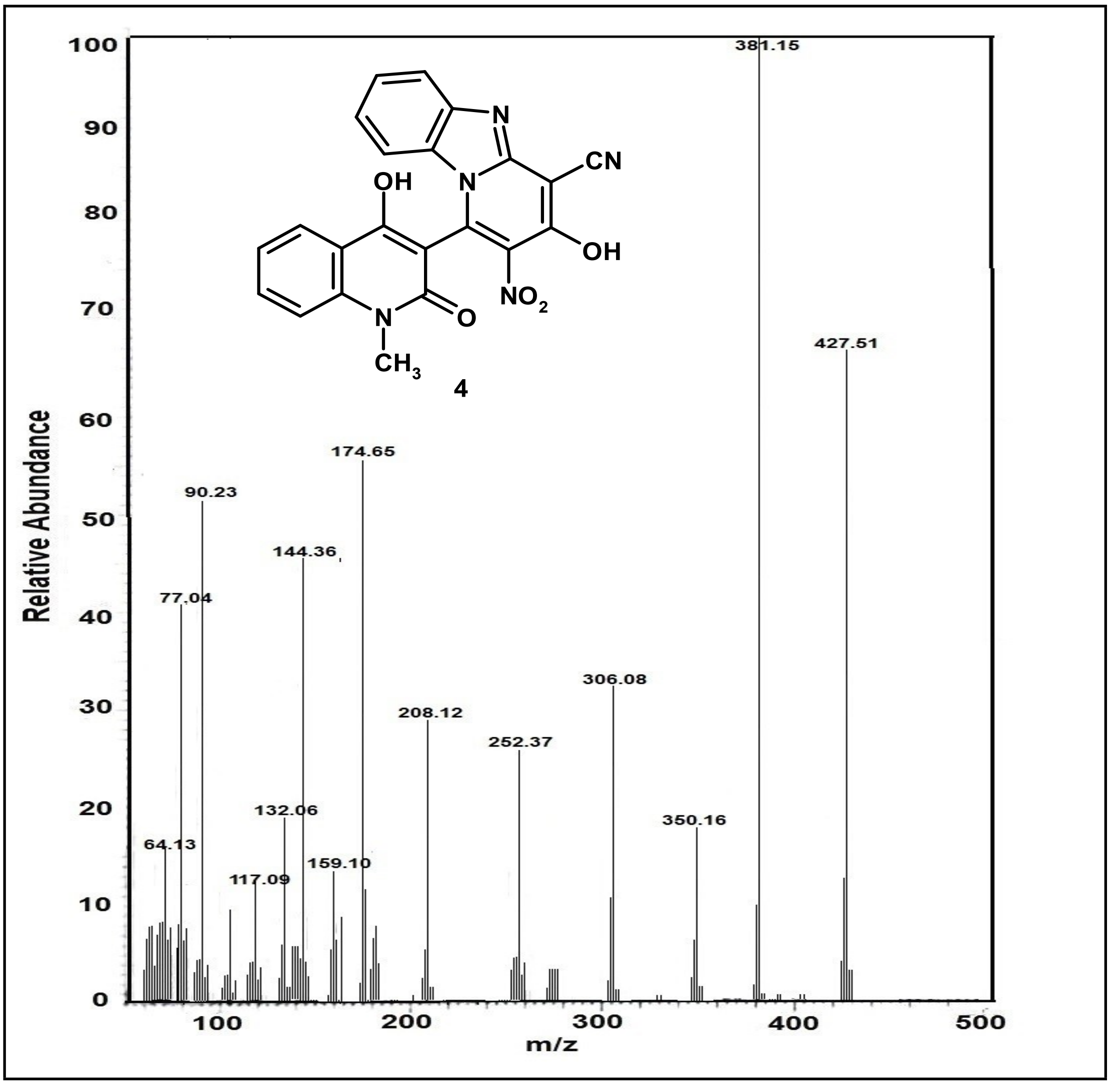

Fig. S16. Mass spectrum of compound **4**

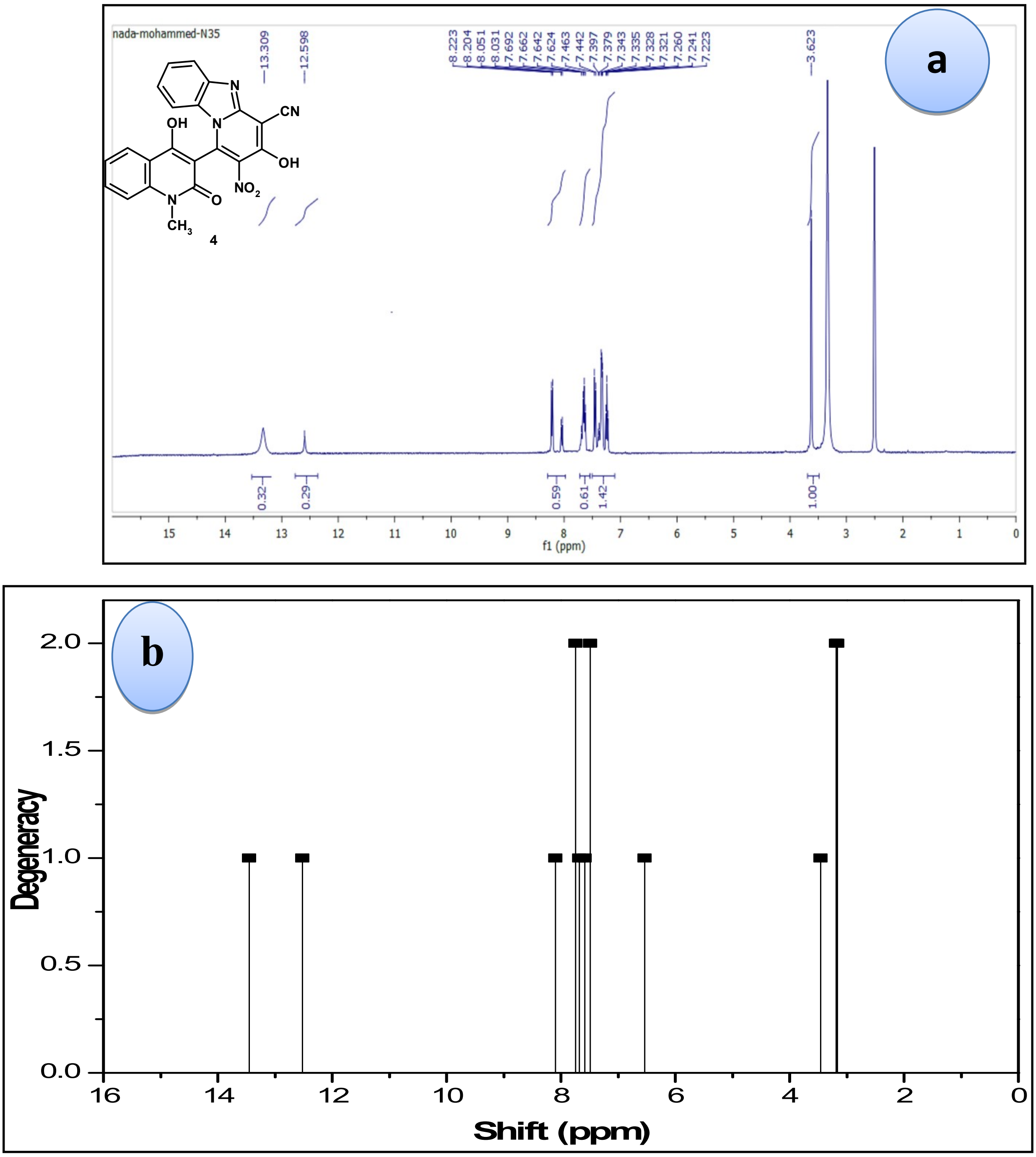

Fig. S17. (a) Experimental and (b) Calculated ^{1}H NMR spectra of compound **4** at B3LYP/6-311++G(d,p)

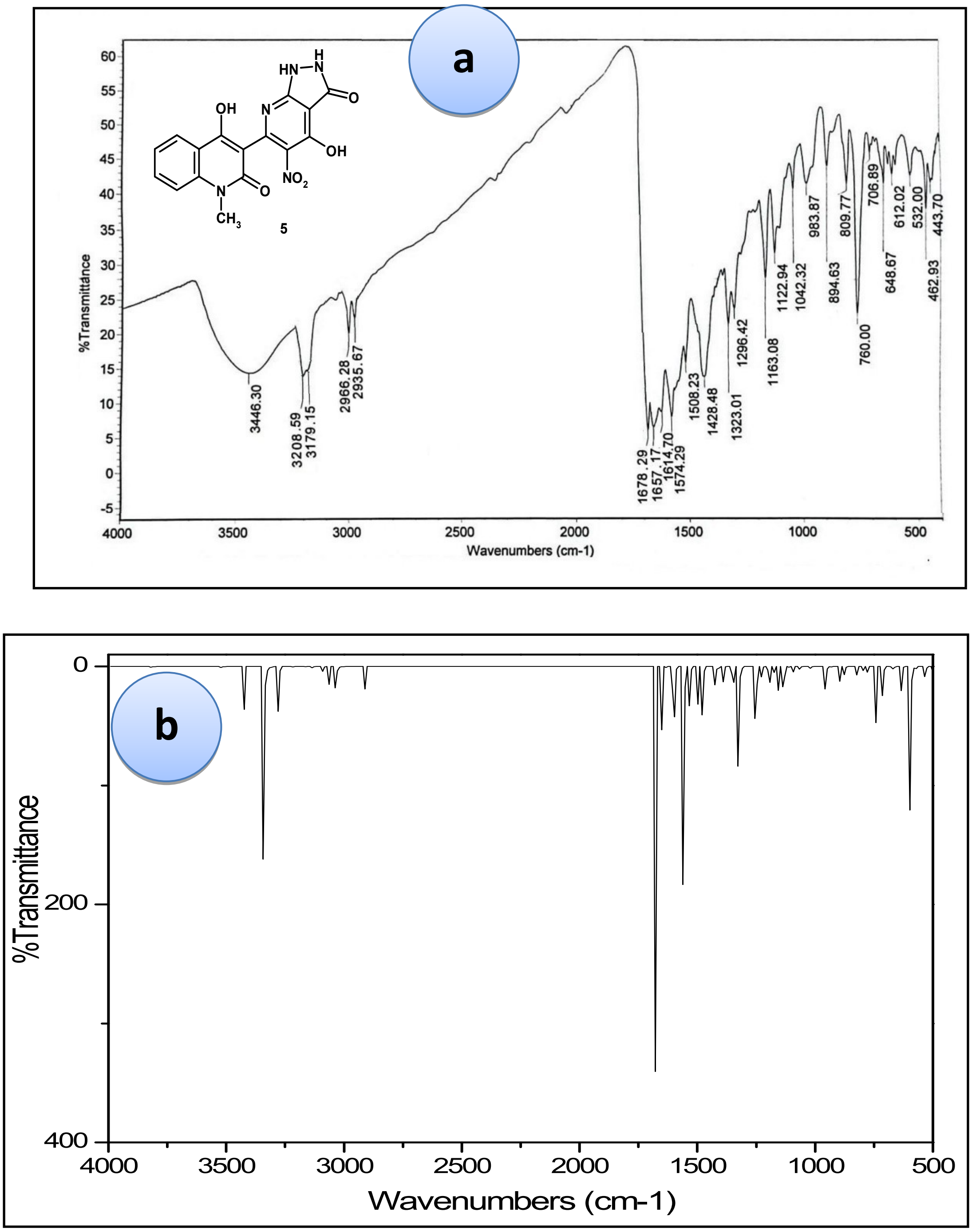

Fig. S18. (a) Experimental and (b) Calculated IR spectra of compound **5** at B3LYP/6-311++G(d,p).

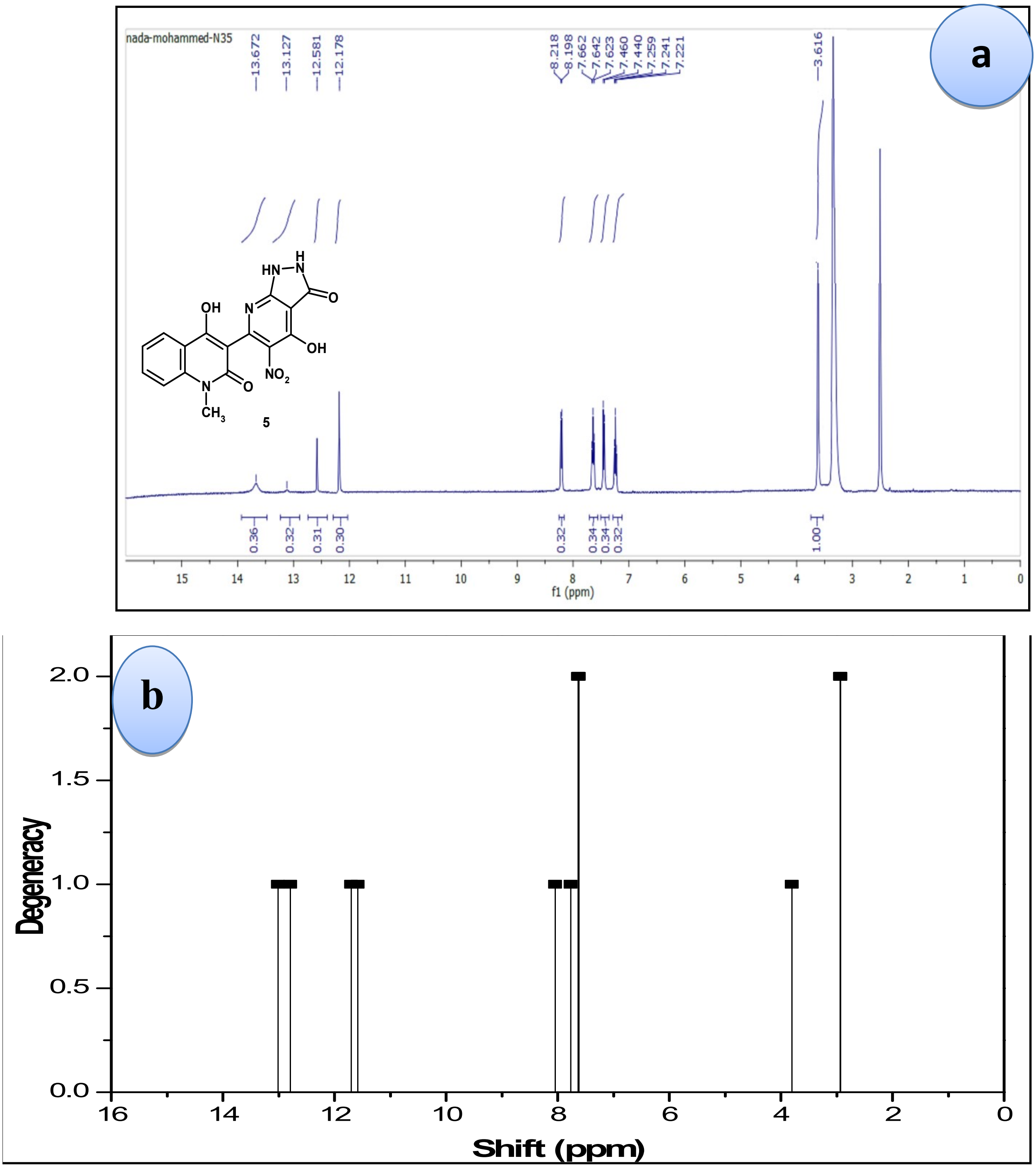

Fig. S19. (a) Experimental and (b) Calculated ^{1}H NMR spectra of compound **5** at B3LYP/6-311++G(d,p).

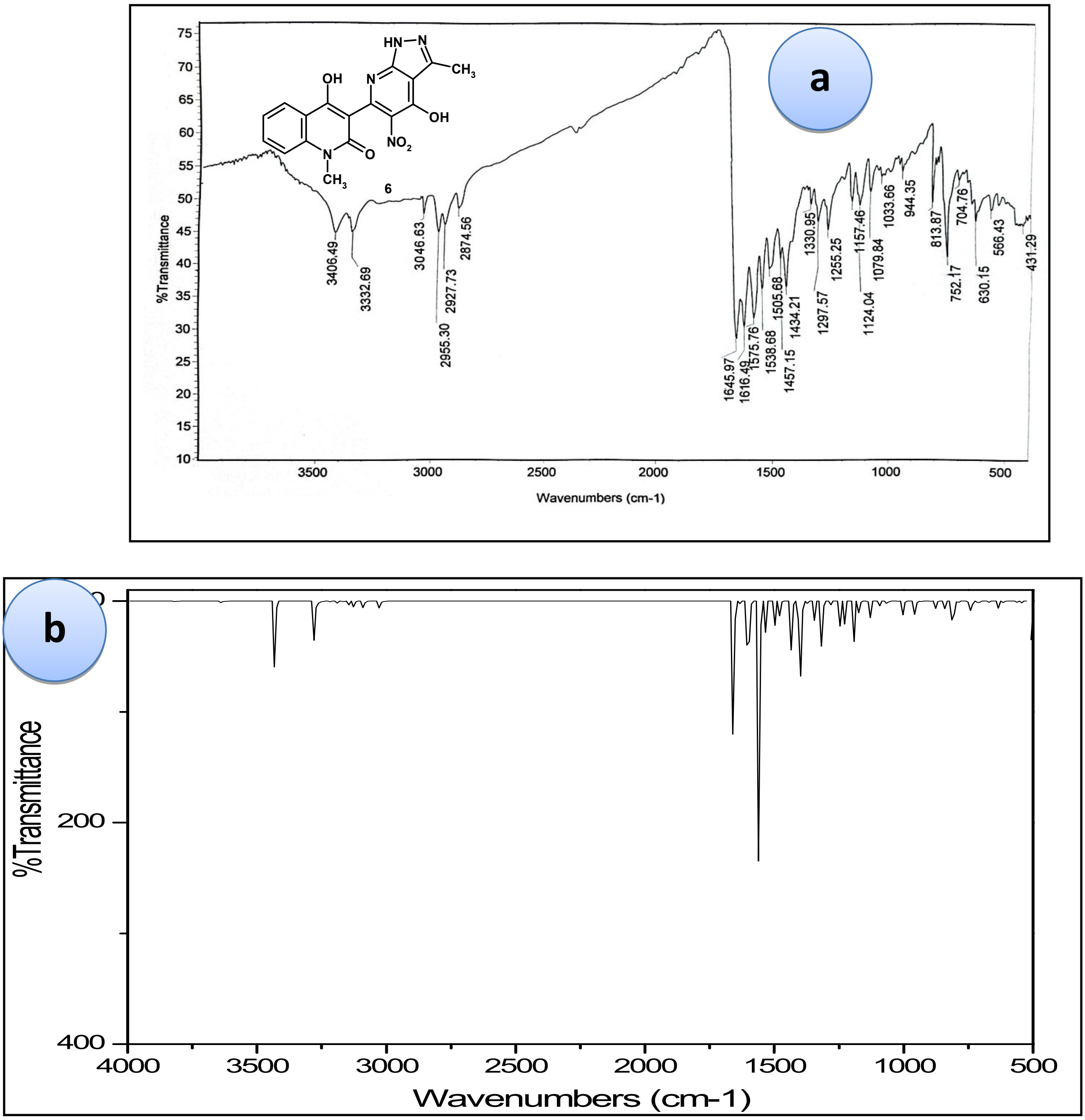

Fig. S20. (a) Experimental and (b) Calculated IR spectra of compound **6** at B3LYP/6-311++G(d,p).

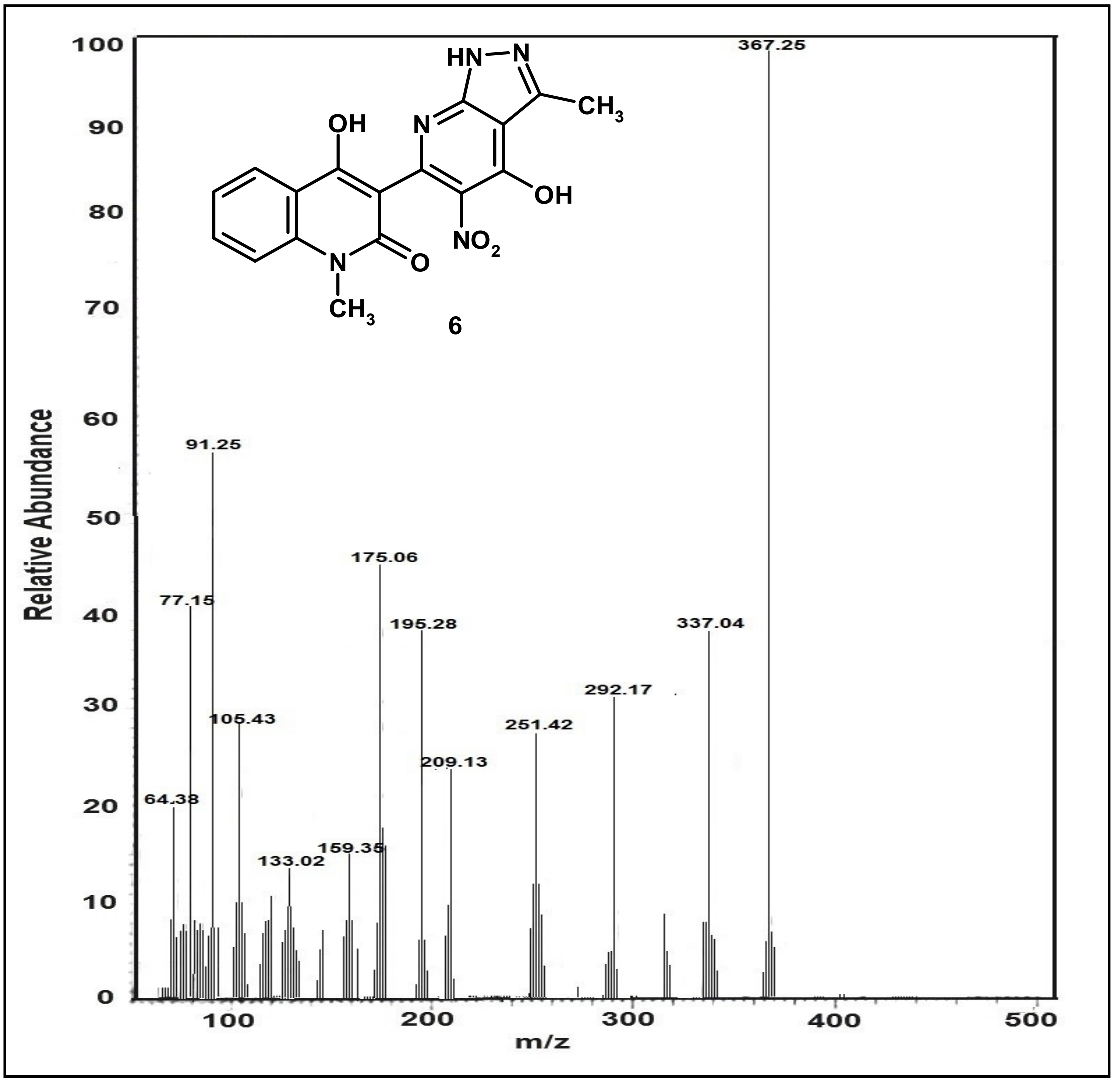

Fig. S21. Mass spectrum of compound **6**

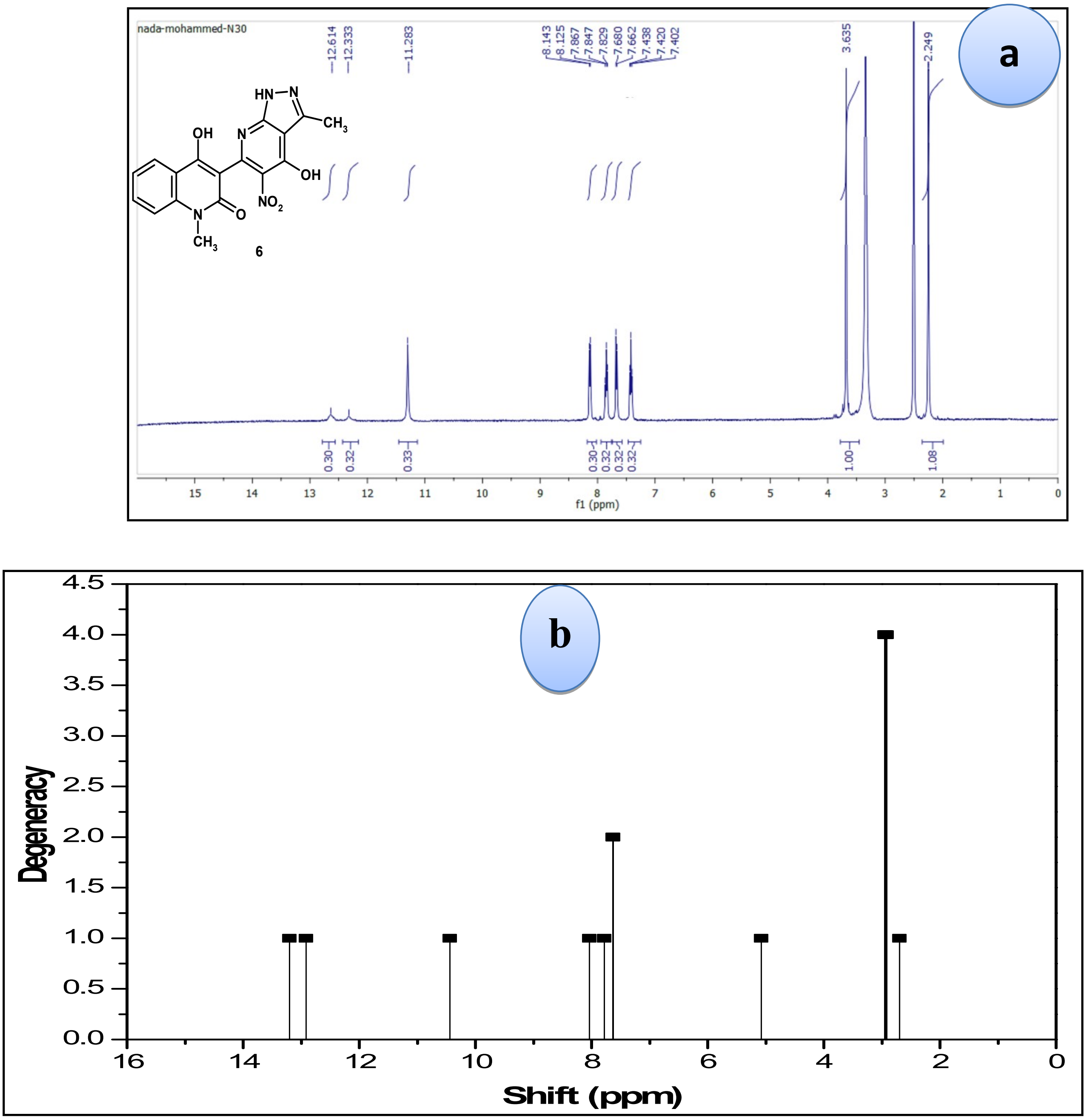

Fig. S22. (a) Experimental and (b) Calculated ^{1}H NMR spectra of compound **6** at B3LYP/6-311++G(d,p).

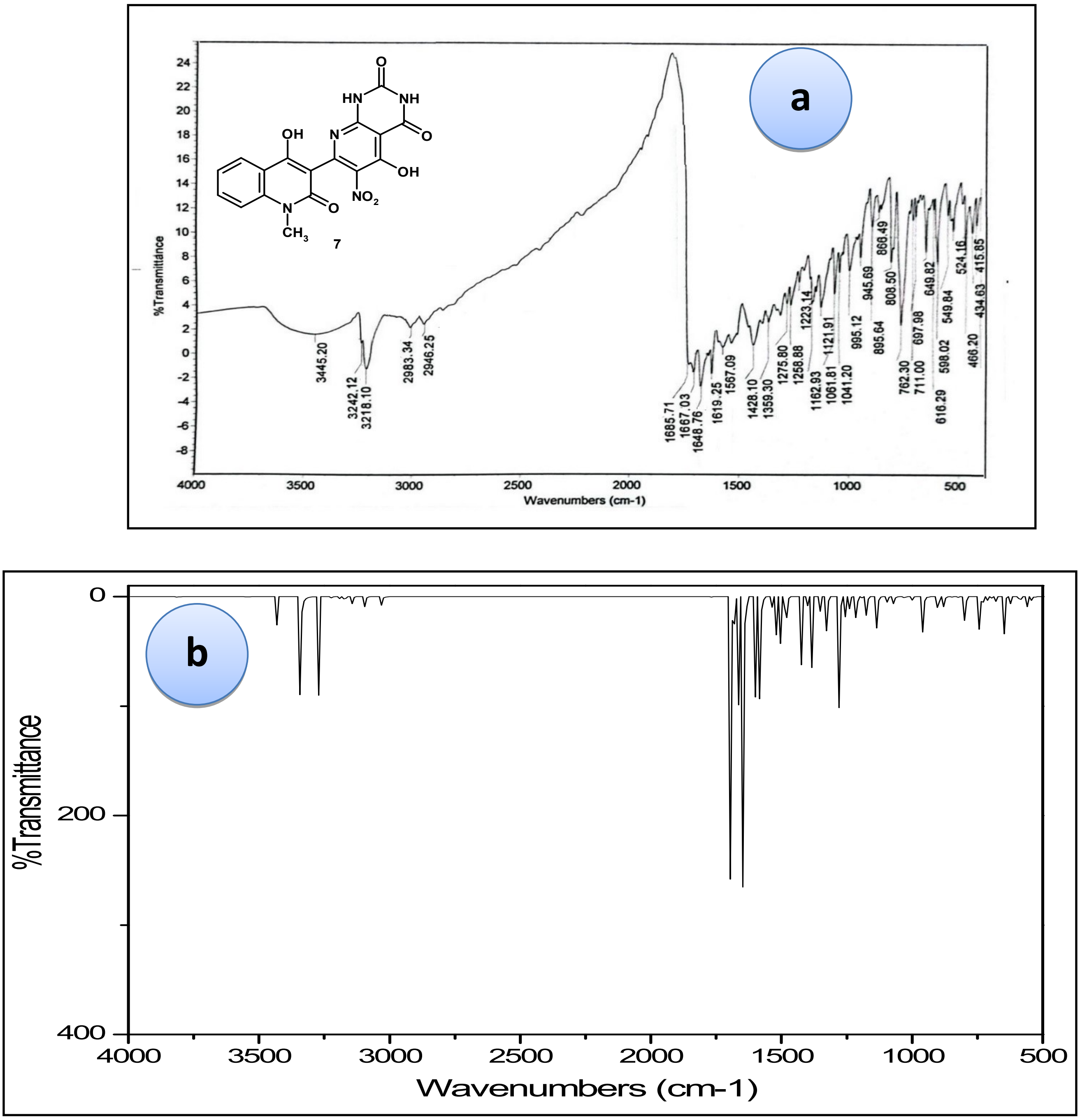

Fig. S23. (a) Experimental and (b) Calculated IR spectra of compound **7** at B3LYP/6-311++G(d,p).

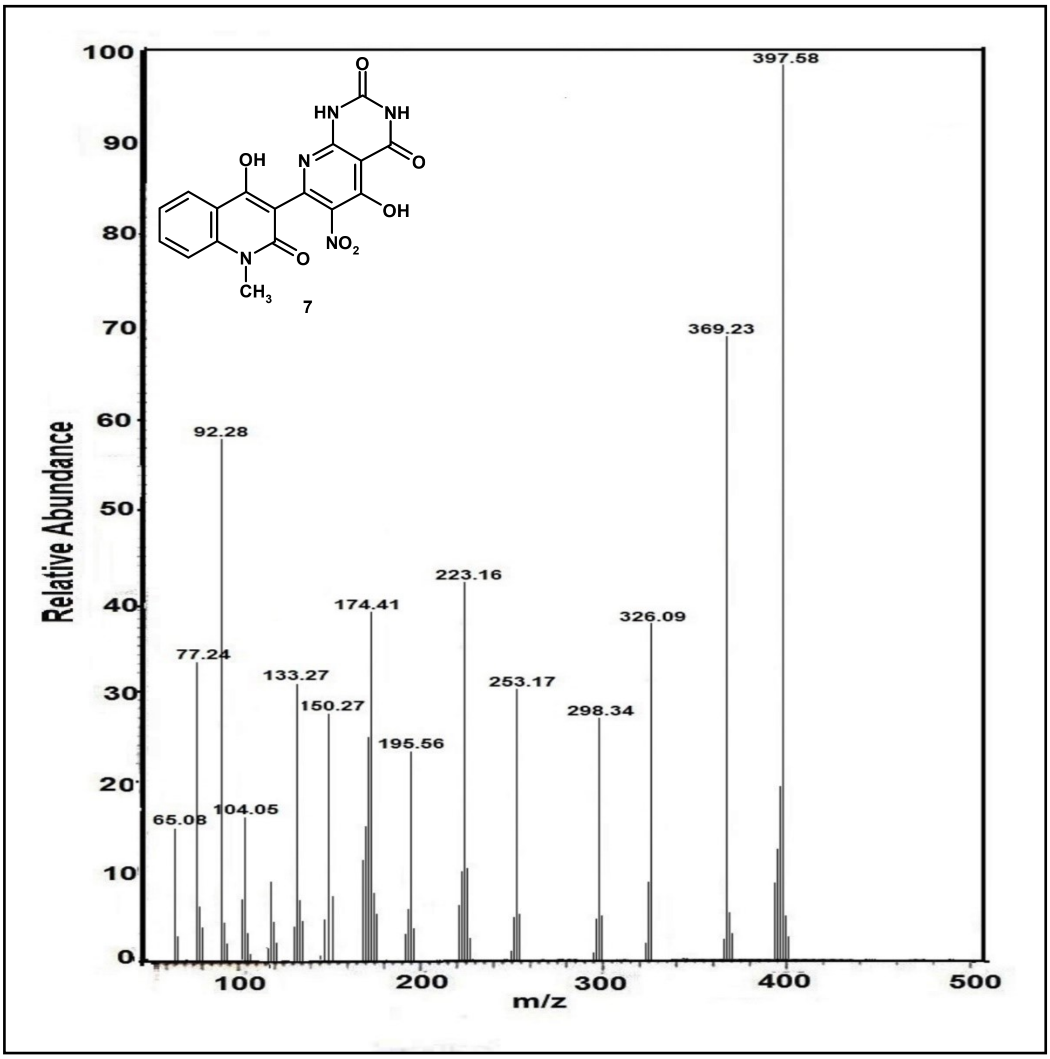

Fig. S24. Mass spectrum of compound **7**

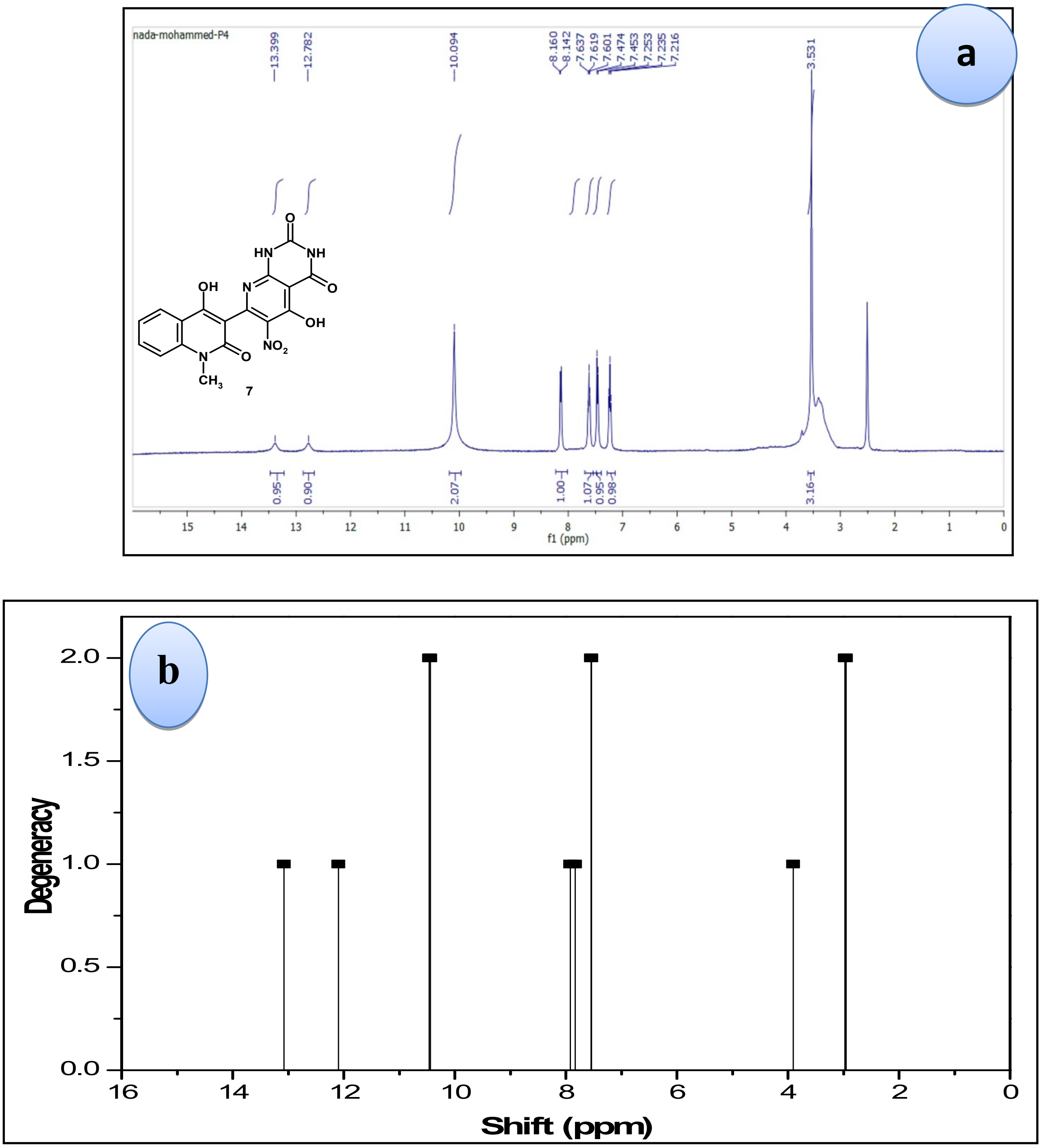

Fig. S25. (a) Experimental and (b) Calculated ^{1}H NMR spectra of compound **7** at B3LYP/6-311++G(d,p).

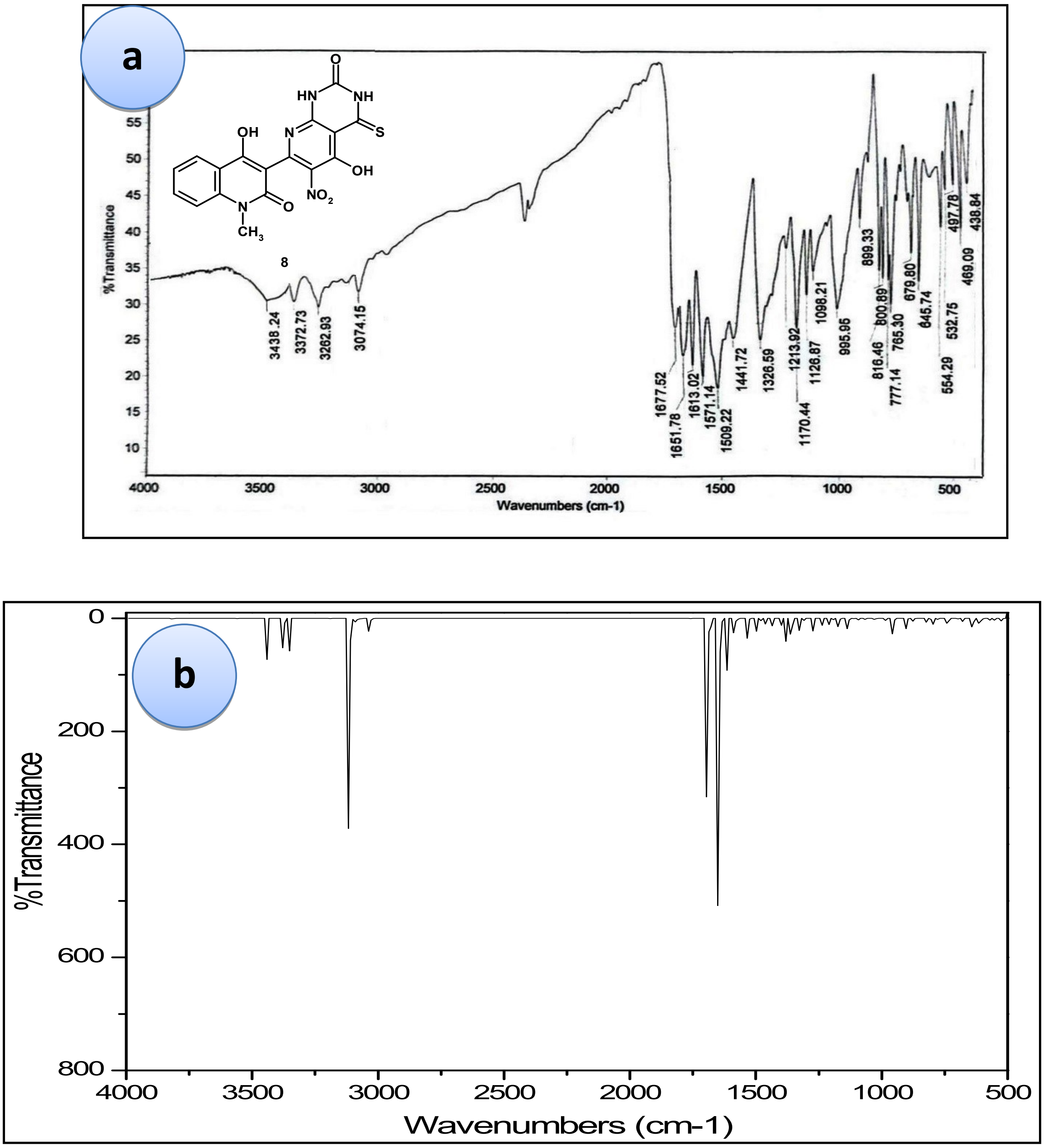

Fig. S26. (a) Experimental and (b) Calculated IR spectra of compound **8** at B3LYP/6-311++G(d,p).

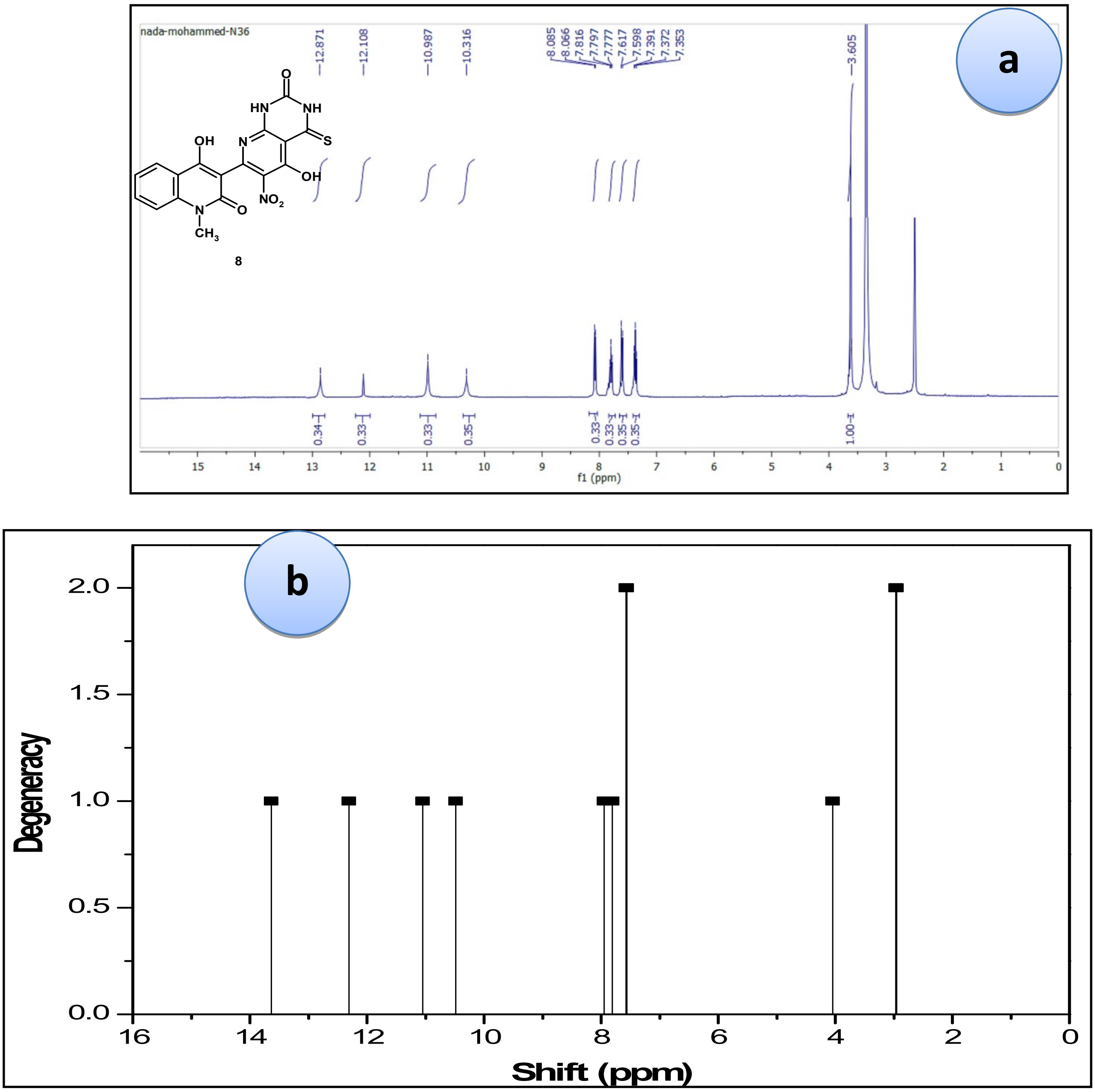

Fig. S27. (a) Experimental and (b) Calculated ^{1}H NMR spectra of compound **8** at B3LYP/6-311++G(d,p).

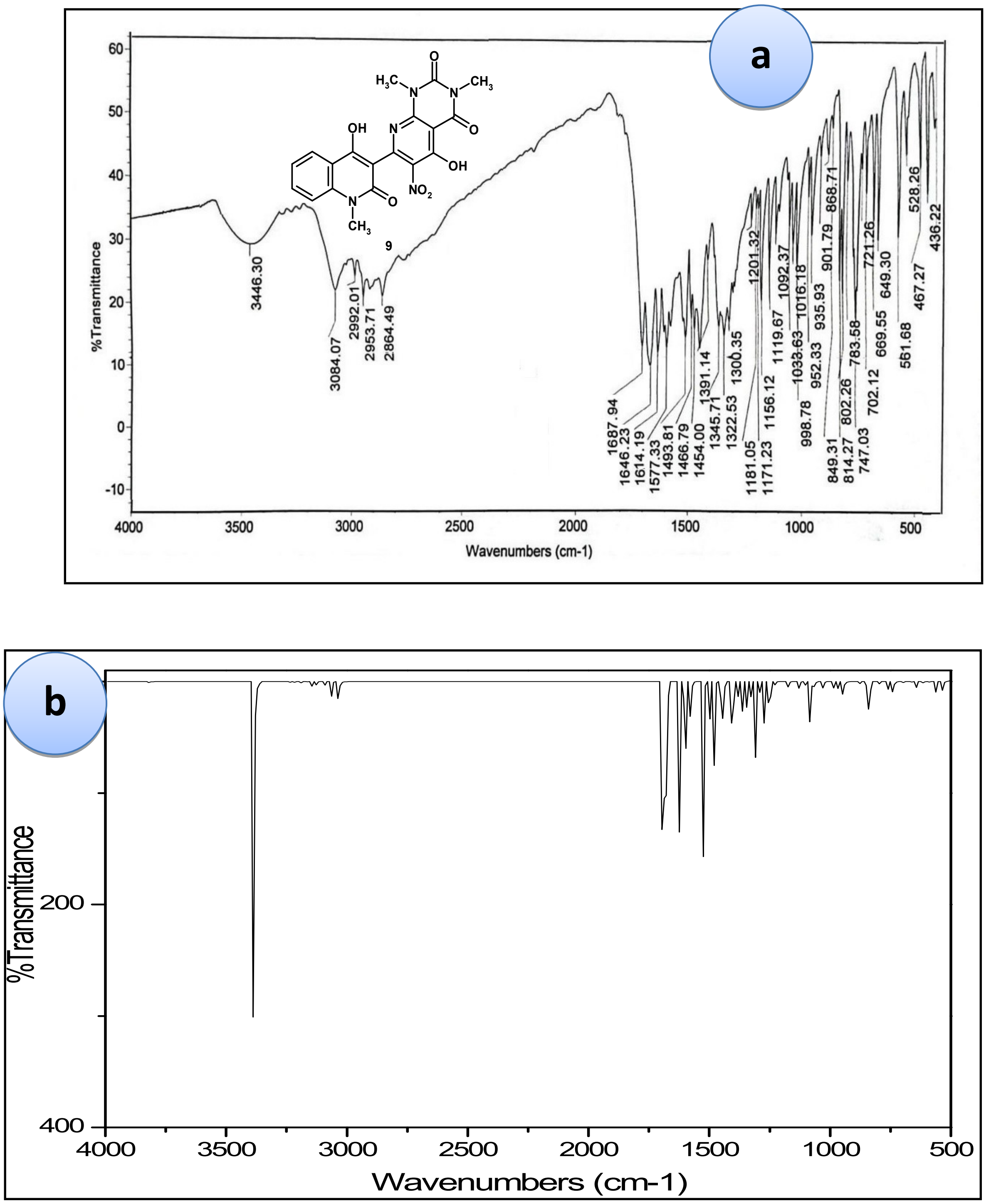

Fig. S28. (a) Experimental and (b) Calculated IR spectra of compound **9** at B3LYP/6-311++G(d,p).

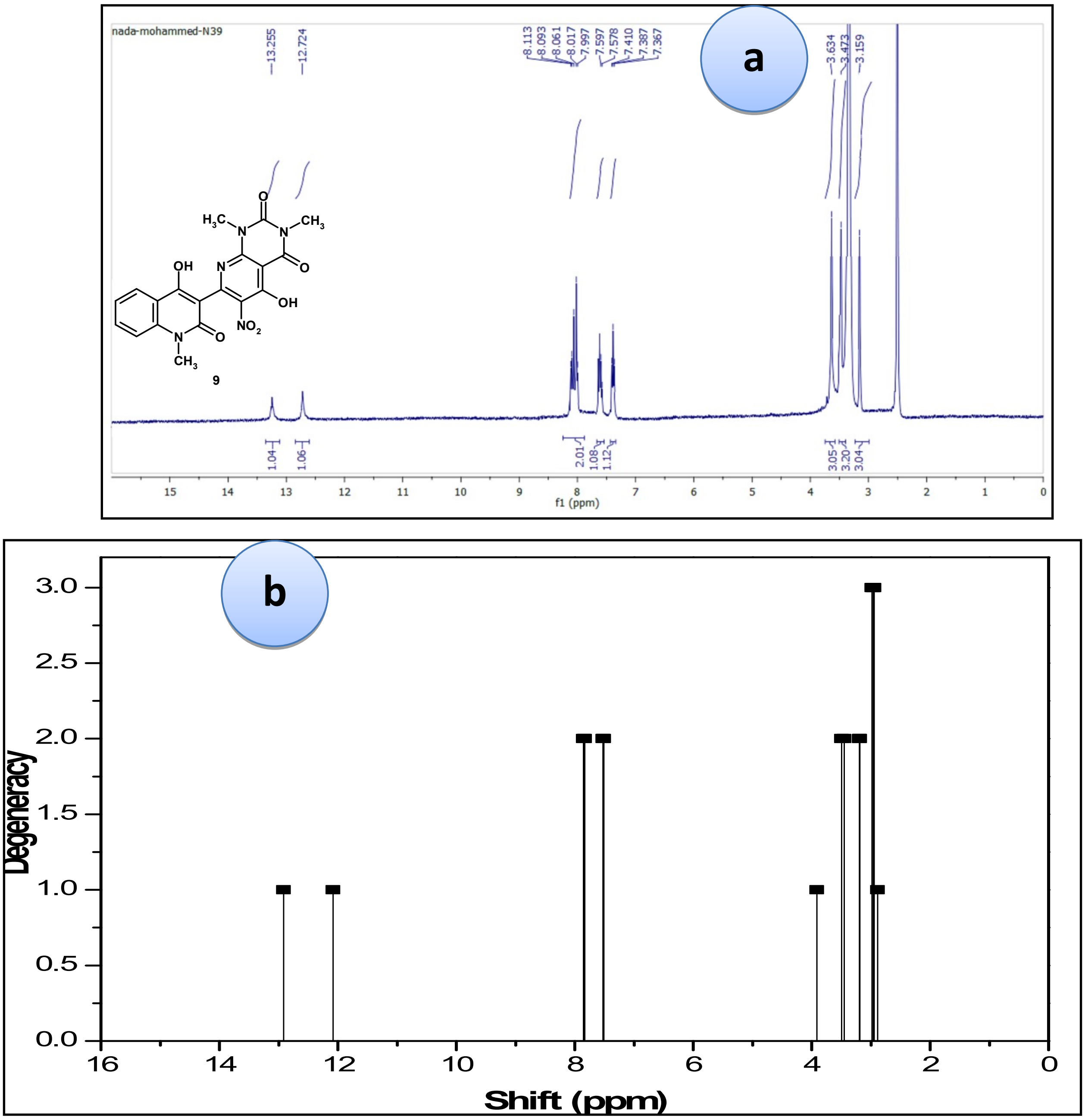

Fig. S29. (a) Experimental and (b) Calculated ^{1}H NMR spectra of compound **9** at B3LYP/6-311++G(d,p).

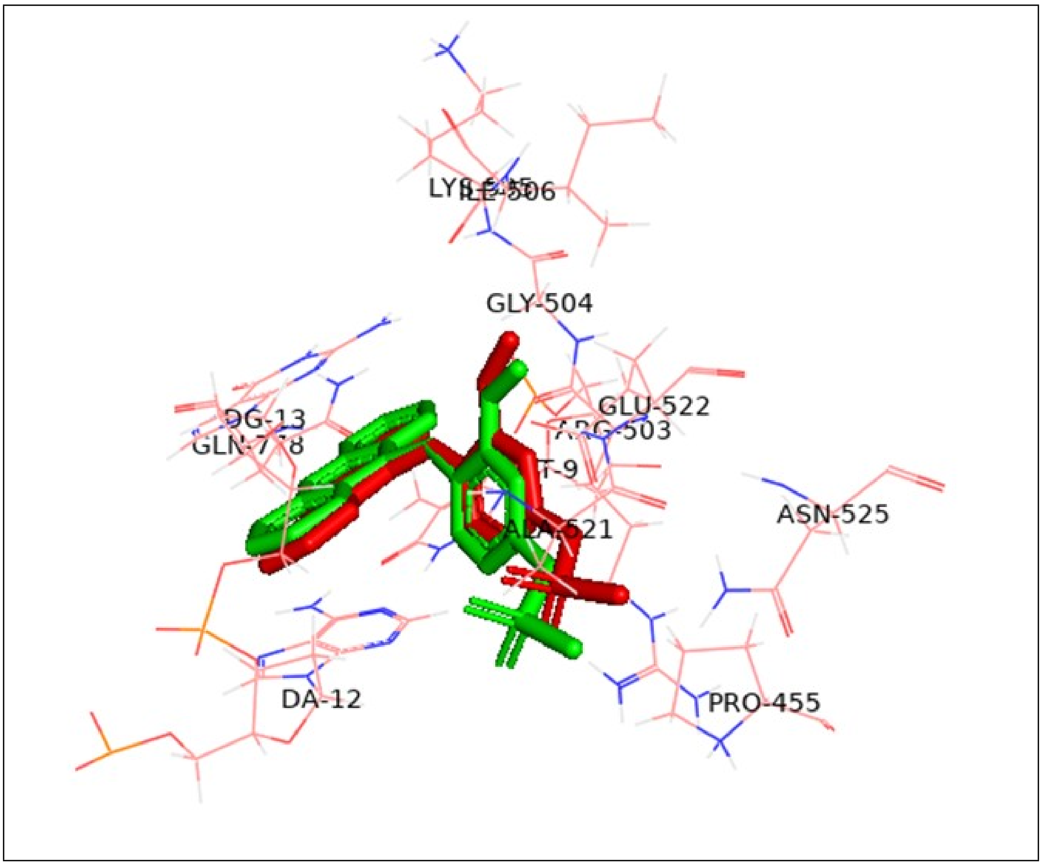

Fig. S30. 3D representation of the superimposition of the co-crystallized (green) and the docking pose (red) of the N-[4-(acridin-9-ylamino)-3-methoxyphenyl] methanesulfonamide ligand in target protein (pdb ID: 4G0U).

Compound **4**

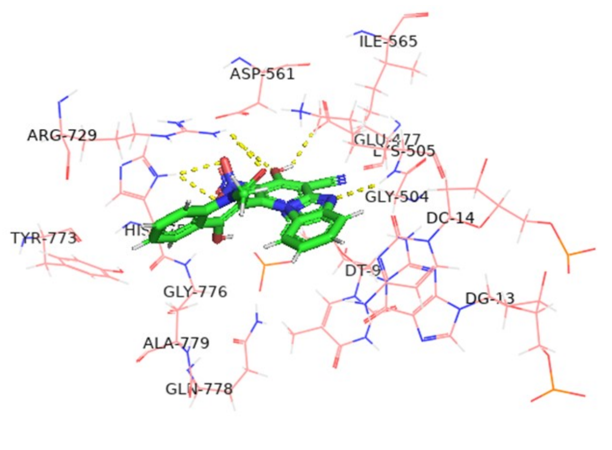

Compound **5**

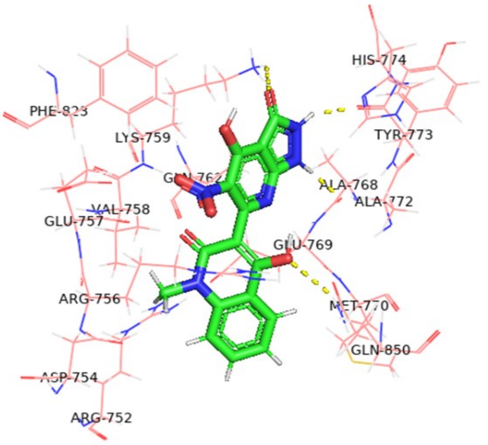

Compound **6**

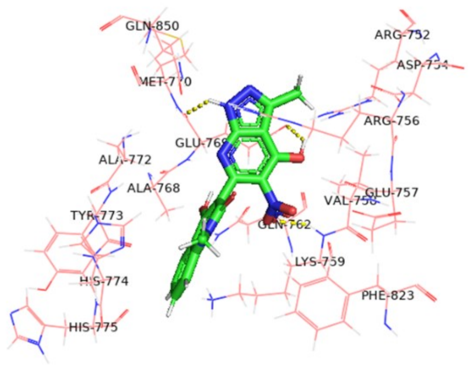

Compound **7**

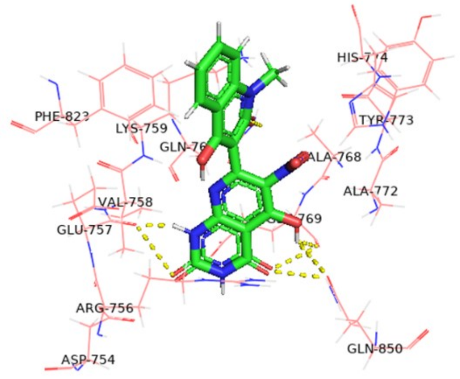

Compound 8

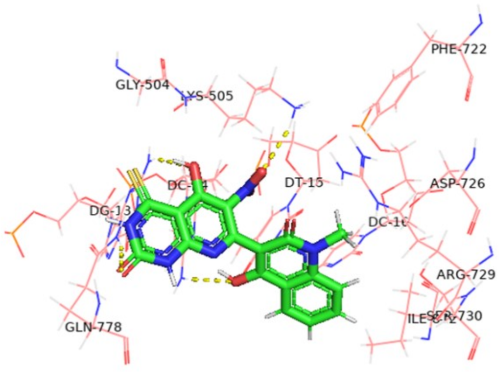

Compound 9

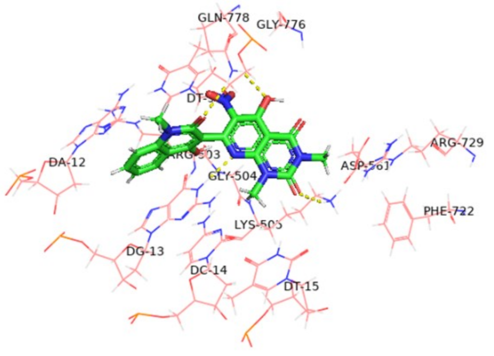

Fig. S31. 3D representation of the Hydrogen bonding between the studied compounds (**4-9**) and the amino acids residues of the target protein (PDB ID: 4G0U).
